# Structure–Activity Relationship Study of Anti-*Cryptosporidium* Benzoxaboroles Yields Enhanced Potency and Curative Efficacy

**DOI:** 10.1021/acs.jmedchem.6c01707

**Published:** 2026-07-27

**Authors:** Soumitra Guin, José E. Teixeira, Peter Miller, Ankita Sarkar, Christopher D. Huston, Marvin J. Meyers

**Affiliations:** † Department of Chemistry, 7547 Saint Louis University, Saint Louis, Missouri 63103, United States; ‡ Department of Medicine, University of Vermont Larner College of Medicine, Burlington, Vermont 05401, United States; § Institute for Drug and Biotherapeutic Innovation, Saint Louis University, St. Louis, Missouri 63103, United States

## Abstract

Cryptosporidiosis is a major cause of life-threatening diarrhea in children and chronic diarrhea in immunocompromised individuals. Our previous work identified pyrazolo­[3,4-*d*]­pyrimidine benzoxaborole (**2**) as an orally efficacious inhibitor of *Cryptosporidium* with a downside of relapse after 7 days post drug treatment. Here, we report a structure–activity relationship study around the benzoxaborole ring of compound **2**, resulting in *rac-* **11** with improved *in vitro* potency (EC_50_ = 0.025 μM). Remarkably, *rac-* **11** is noncytotoxic and displayed curative *in vivo* efficacy in *Cryptosporidium*-infected immunocompromised NSG mice. Furthermore, resolution of *rac*-**11** into its enantiomers demonstrated that one enantiomer is 2-fold more potent (*ent2*-**11**; EC_50_ = 0.011 μM), whereas the other is 400-fold less potent, highlighting the importance of the methyl group at the C3 position of benzoxaborole. The *ent2*-**11** also had reduced adverse binding to *h*PDE5 compared to **2**. Collectively, these findings advance new and effective candidates for the treatment of cryptosporidiosis.

## Graphical abstract

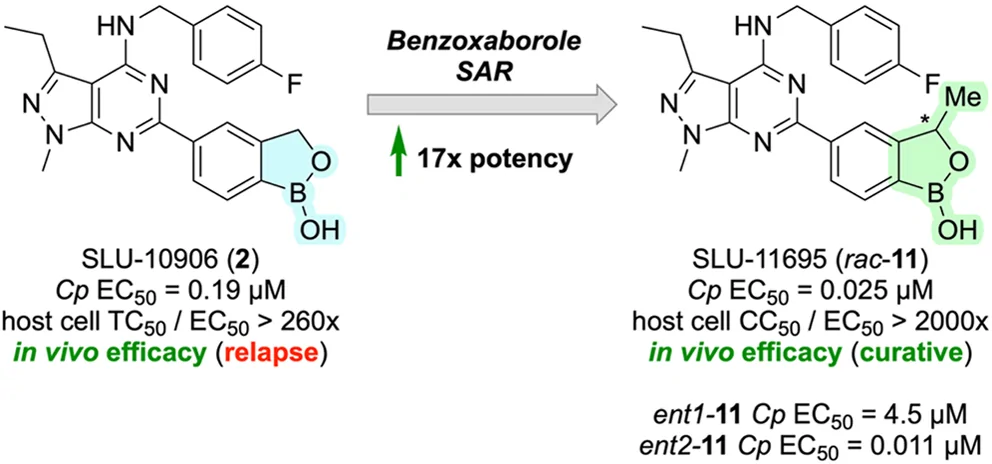

## Introduction

*Cryptosporidium* was identified more than a century ago by E.E. Tyzzer, and then in 1976, it was recognized as a significant cause of gastrointestinal illness (cryptosporidiosis) in humans with diarrhea-like symptoms.^1^ Since then, more than 100,000 deaths per year have been reported globally, which makes the situation a concerning global health burden.^2^ It is deadly, especially to malnourished children and people with a weakened immune system, such as AIDS and organ-transplant patients.^2–4^ Furthermore, cryptosporidiosis ranks among the leading causes of life-threatening diarrheal disease in livestock, resulting in substantial economic losses.^5^

Nitazoxanide (NTZ) was approved by the FDA in 2002, and it is the only approved drug for cryptosporidiosis (Figure 1). However, it poses risks for children under the age of 1 year and is ineffective for immunocompromised patients.^6^ In 2013, an epidemiological study published by Kotloff et al. spread awareness of the prevalence and burden of *Cryptosporidium* spp., triggering research efforts toward finding effective therapeutic options.^7^ Direct repurposing of the drug clofazimine failed in clinical trials.^8^ Since then, a handful of preclinical candidates have emerged, including inhibitors of CDPK1 (calcium-dependent protein kinase 1; e.g., bumped kinase inhibitors BKI-1369, BKI-1770),^9–11^ methionyl-, phenyl-, and lysyl-*t*RNA synthetases (e.g., UW2093, DDD01510706, BRD-7929),^12–15^ CPSF3 (cleavage and polyadenylation specificity factor 3; e.g., AN-7973),^16,17^ and promising compounds with unknown targets (e.g., SLU-2633)^18^ (Figure 1). The only clinical candidate in the pipeline is an inhibitor of phosphatidylinositol-4-OH kinase (PI4K), EDI048, which recently completed phase 1 clinical trials.^19,20^ Therefore, there is a pressing need to develop additional chemotypes with novel targets as candidates for the treatment of cryptosporidiosis.

**Figure 1:**
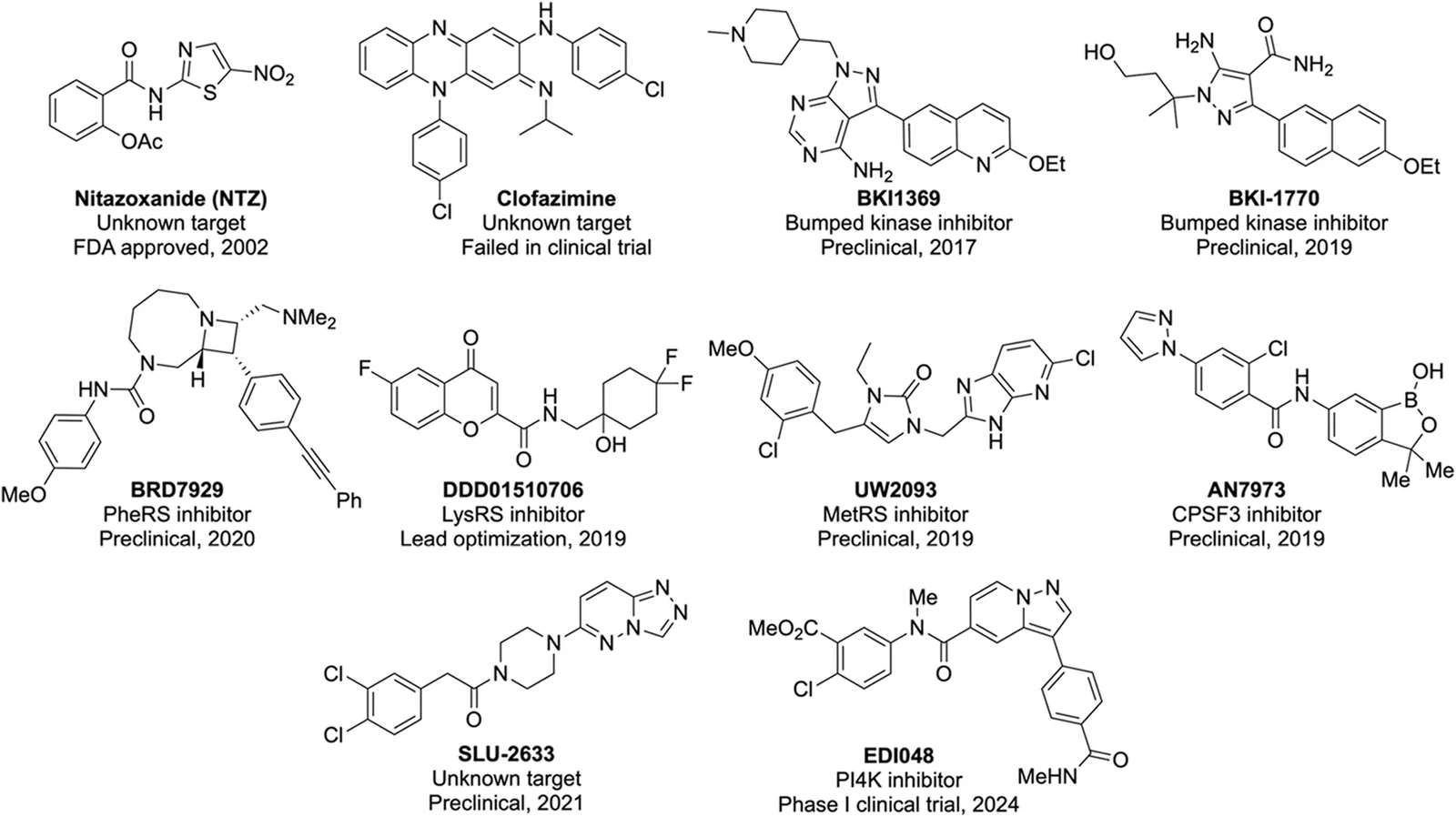
Structure of advanced inhibitors of *Cryptosporidium*.

Phosphodiesterases play a vital role in regulating internal cyclic nucleotide concentrations (i.e., cAMP and cGMP), which are key molecules in cell signaling, differentiation, and survival within the host. Interfering with phosphodiesterase (PDE) activity disrupts these essential signaling pathways. *Cryptosporidium* encodes three PDEs, only one of which (*Cp*PDE1), is expressed during asexual parasite growth.^21,22^ Importantly, structural differences between parasite and human PDEs allow for the potential to develop selective inhibitors that target the parasite enzymes while leaving human enzymes largely unaffected. Previous research from our group identified pyrazolo­[3,4-*d*]­pyrimidine SLU-2815 (**1**; Figure 2) as a potent *Cryptosporidium parvum* growth inhibitor that targets *Cp*PDE1, rapidly eliminates parasites from culture, and causes defects in the parasite’s exit from host cells, demonstrating direct and lethal action.^23^ SAR studies around the benzoic acid position led to the discovery of more potent benzoxaborole SLU-10906 (**2**),^24^ which may also target *Cp*PDE1, but that remains to be conclusively demonstrated.

**Figure 2:**
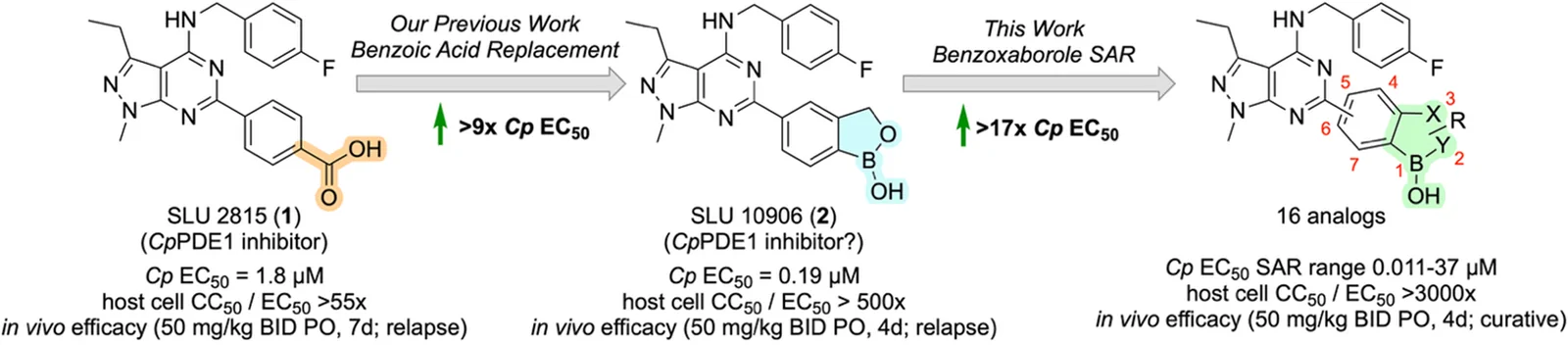
Evolution of pyrazolopyrimidine series and development of benzoxaborole SAR.

Benzoxaboroles represent a novel class of chemically versatile and pharmacologically diverse boron-containing heterocycles, offering broad therapeutic potential including recently FDA-approved tavaborole for treating onychomycosis, crisaborole for eczema, and the investigational acoziborole currently in clinical trials for trypanosomiasis (Figure 3).^25^ Because of the empty p-orbital on the boron atom, which permits it to equilibrate between its neutral trigonal planar state and a stable anionic tetrahedral borate form, the properties of benzoxaboroles are intriguing and can be game-changers during the lead optimization stage. Generally, they are stable *in vivo* and often offer enhanced bioavailability, lower toxicity, and druglike pharmacokinetic (PK) properties relative to boronic acids.

**Figure 3:**
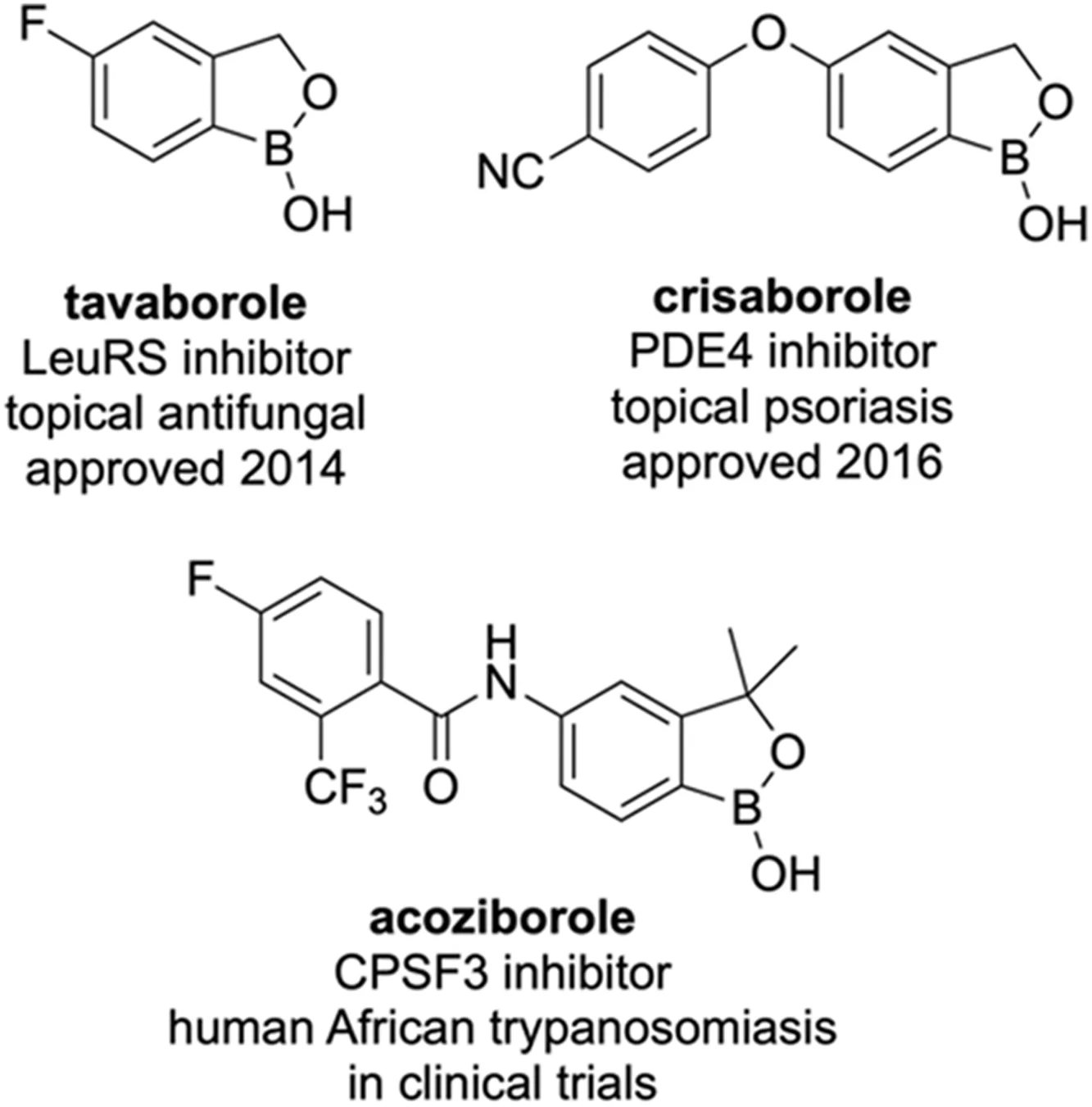
Examples of FDA-approved and clinical benzoxaboroles.

Replacement of the benzoic acid in **1** with benzoxaborole significantly improved the anti-*Cryptosporidium* potency in both *in vitro* and head-to-head *in vivo* studies.^24^ Benzoxaborole **2** is highly selective as an anti-*Cryptosporidium* agent over cytotoxicity (>500-fold) and is orally efficacious *in vivo* in a *Cryptosporidium-*infected NSG mouse model, although relapse was observed 1 week post removal of treatment.^24^ Thus, benzoxaborole **2** was selected as a promising lead for further antiparasitic drug development.

Using a model of *Cp*PDE1 derived from AlphaFold,^23^ we docked **2** in the active site with the benzoxaborole moiety in the hydrated form using the Induced Fit Docking routine in Schrödinger Maestro (version 2025-1) with no constraints, giving a docking score of −9.3 for **2**. As expected, the benzoxaborole is predicted to interact with the active site Zn and Mg ions in the *Cp*PDE active site via ionic interactions (Figure 4), in a manner consistent with that observed experimentally for a crisaborole analog AN2898 with human PDE4 (PDB code 3O0J).^26^ The pyrazolo­[3,4-*d*]­pyrimidine core is predicted to form π-π interactions with Phe935 and a hydrogen bond from the N2 position of the pyrazole with Gln932 and another π-π interaction with the pendant benzene ring of the benzoxaborole with His725. It was, however, unclear from docking studies whether the regioisomeric benzoxaborole or other substitutions would be tolerated. There does appear to be unoccupied space adjacent to the benzoxaborole ring. Thus, we hypothesized that SAR studies of the benzoxaborole ring may result in more potent analogs.

**Figure 4:**
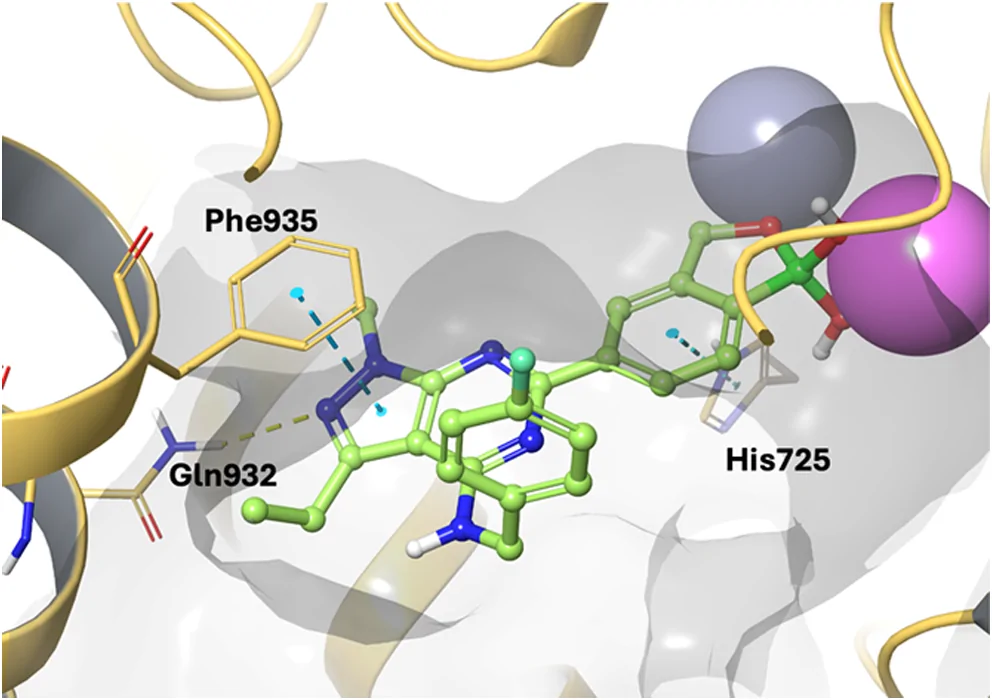
Induced fit docking pose of SLU-10906 (**2**) with the *Cp*PDE1. The model of *Cp*PDE1 was derived from AlphaFold homology model A3FQ29-F1-model_v4 and the crystal structure of *Schistosoma mansoni* PDE4 (PDB code 6EZU) as previously described.^23^

It is important to note, however, that the benzoxaborole series may have picked up a secondary target, lending caution to over-reliance on docking studies with the *Cp*PDE1 model. Thus, we aimed to conduct a more extensive empirical SAR study that not only profiled substitutions predicted to improve potency but also examined regioisomers of benzoxaborole and modification of the size and constitution of the oxaborole ring while keeping the rest of the molecule fixed. Here, we identified benzoxaboroles with exceptional potency and, for the first time in this series, curative *in vivo* efficacy in the NSG mouse model.

## Results and Discussion

### Synthesis of Benzoxaboroles

We recently developed and reported the synthesis of the pyrazole derivative **3** and lead benzoxaborole **2**.^23,24^ Using the same strategy, bromomethyl ester **4** was synthesized from **3** via an iodine-mediated oxidative cyclization with methyl 2-bromo-5-formylbenzoate (Scheme 1). Next, a BOP/DBU-mediated S_N_Ar-type reaction^27^ with 4-fluorobenzylamine afforded the versatile bromomethyl ester intermediate **5**. Afterward, the ester group of **5** was reduced with DIBAL-H to the corresponding alcohol and then reoxidized to aldehyde with MnO_2_ oxidation to afford bromo benzaldehyde **6**. 3-Substituted benzoxaboroles (**11–14**) were then synthesized from **6** by treating with corresponding Grignard reagents to afford the corresponding alcohols (**7–10**), followed by Pd-catalyzed borylation and cyclization.

**Scheme 1:**
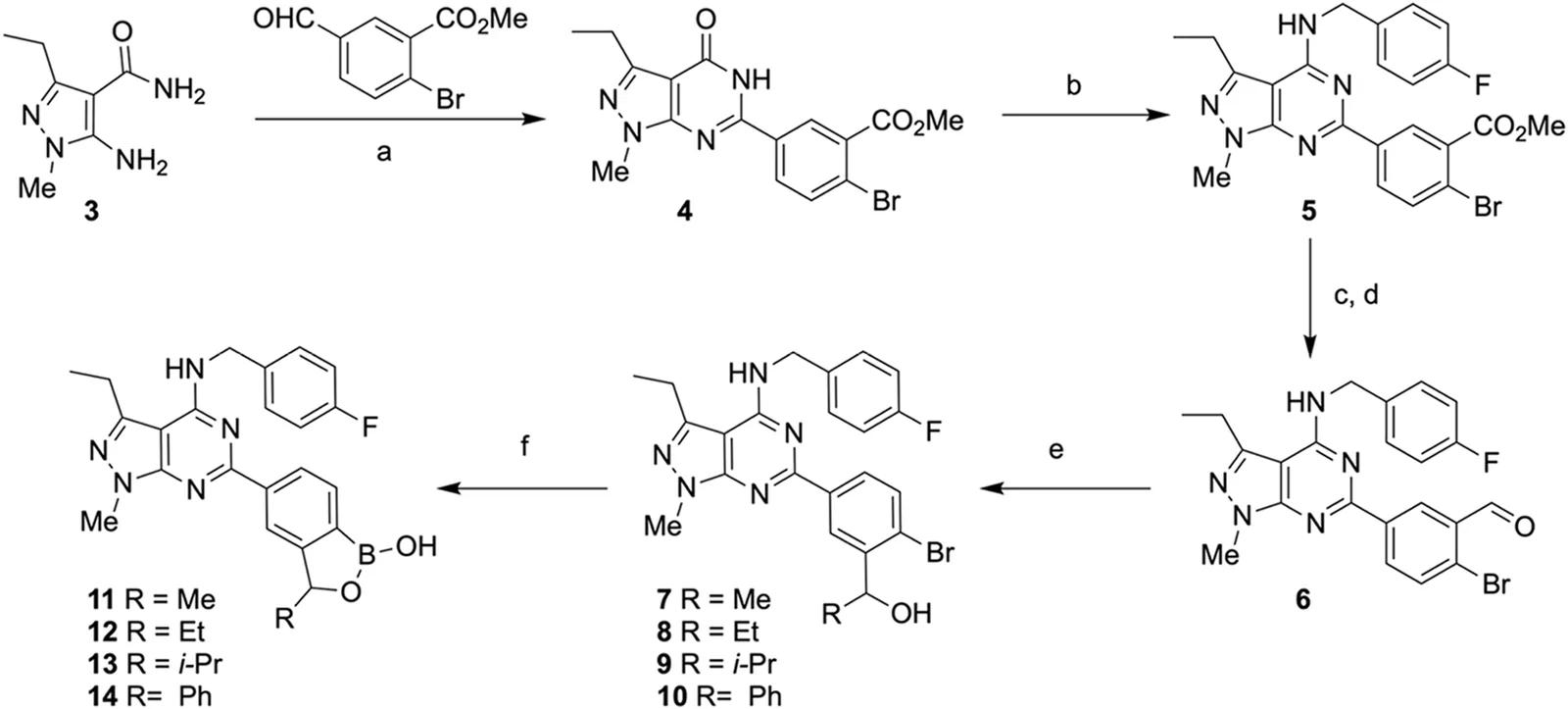
Synthesis of Isomeric Benzoxaborole Compounds[Fn s1fn1]

a
**Reaction conditions:** (a) I_2_, CH_3_CN, reflux, 16 h, 61%; (b) BOP, DBU, 4-fluorobenzylamine, DMF, rt, 16 h, 78%; (c) DIBAL-H, DCM, 0 °C, 2 h, 82%; (d) MnO_2_, DCM, rt, 2 h, 93%; (e) RMgCl, THF, 0 °C-rt, 2–3 h, 48–52%; (f) B_2_(OH)_4_, KOAc, XphosPdG2, EtOH, 80 °C, 1 h, 70–77%.

Next, the intermediate bromo benzoic ester **5** was treated with an excess amount of magnesium chloride to afford the corresponding gem-dimethyl alcohol **15** (Scheme 2). Finally, 3,3-dimethyl derivative **16** was synthesized from the gem-dimethyl alcohol intermediate **15** *via* Pd-catalyzed Miyaura borylation, followed by acidic workup. Additionally, intermediate **5** was transformed into the benzoxaborolone **18** *via* a Pd-catalyzed Miyaura borylation followed by base-mediated hydrolysis of the resultant ester **17** and acid-catalyzed cyclization sequence.

**Scheme 2:**
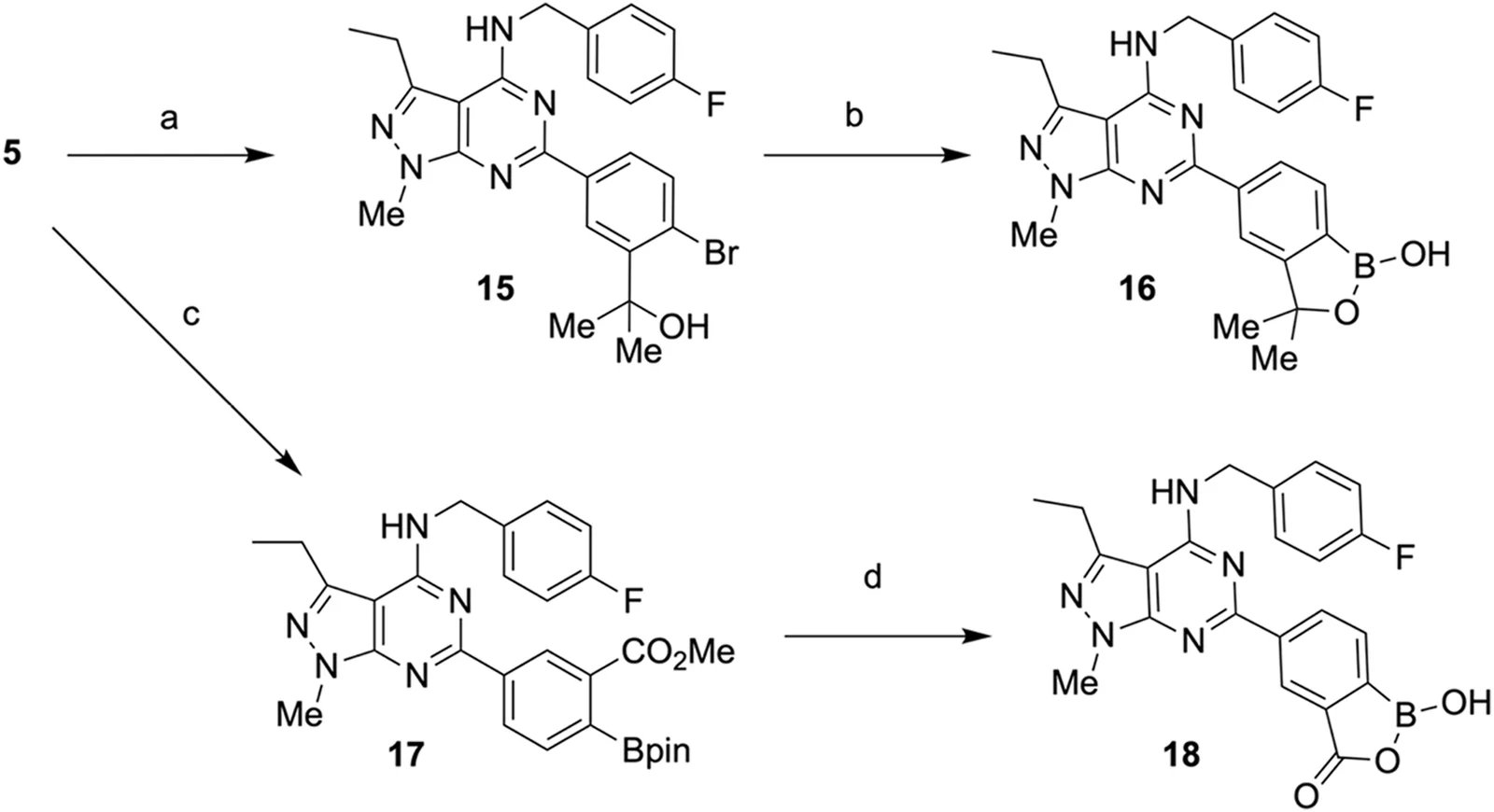
Synthesis of 3,3-Disubstitted Benzoxaborole **16** and Benzoxaborolone **18[Fn s2fn1]**

a
**Reaction conditions:** (a) MeMgCl, 0--60 °C, 4 h, 31%; (b) B_2_(OH)_4_, KOAc, XphosPdG2, EtOH, 80 °C, 1 h, 70–77%; (c) Pd­(dppf)­Cl_2_, KOAc, B_2_pin_2_, 1,4-dioxane, 80 °C; (d) (i) 1.0 M NaOH, MeOH, rt, 2 h; (ii) 1.0 M HCl, rt, 5 min. 15% over 2 steps.

Next, we focused on replacing the methyl group at the 3-position of the benzoxaborole **11** with a trifluoromethyl group. The α-trifluoromethyl alcohol was obtained from bromo benzaldehyde **6** via careful addition of Ruppert-Prakash reagent (TMS-CF_3_) at low temperature (Scheme 3). Later, α-trifluoromethyl alcohol **19** was converted to **20** using Pd-catalyzed borylation, then cyclization.

**Scheme 3:**
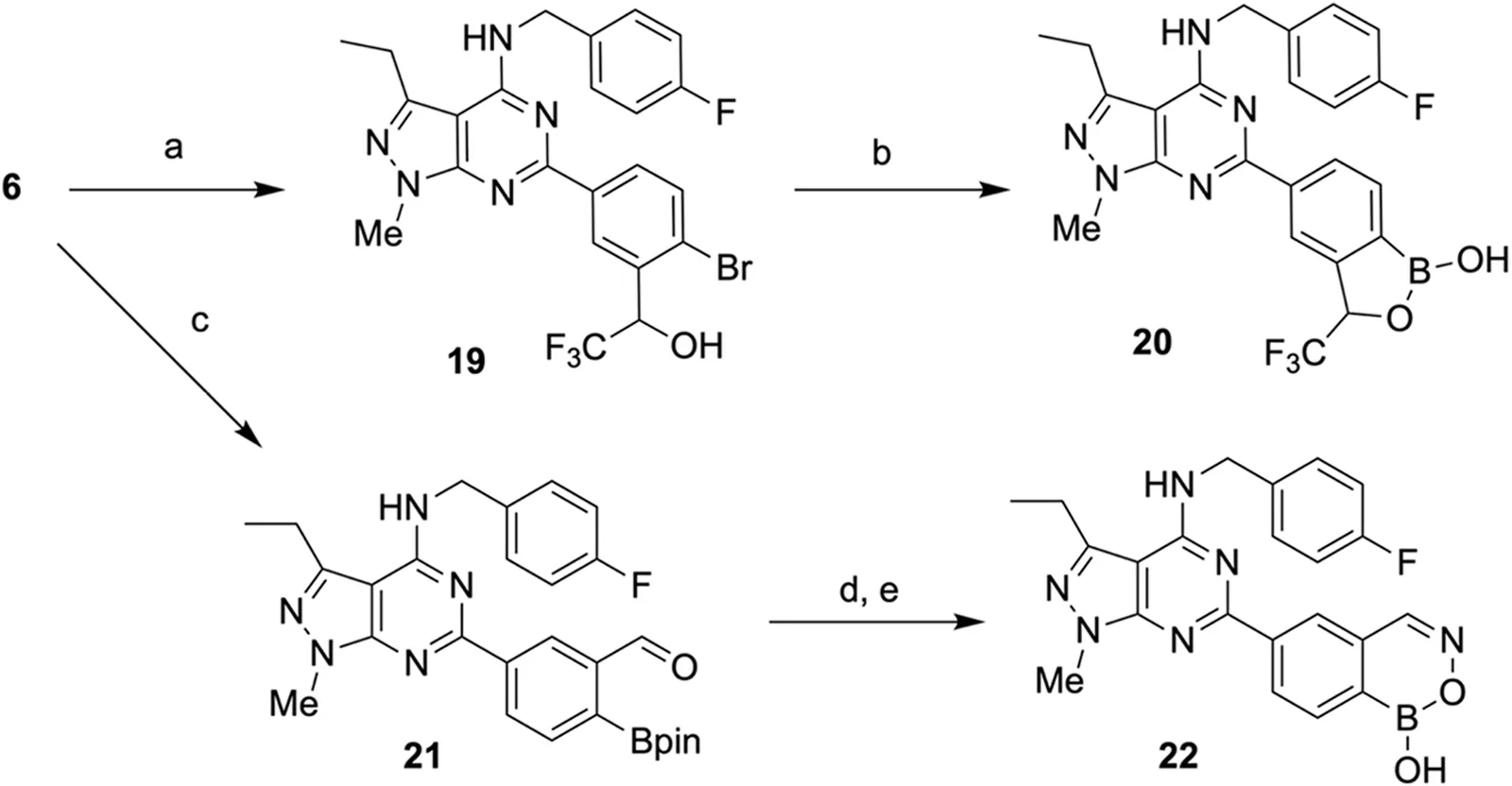
Synthesis of Isomeric Benzoxaborole Compounds[Fn s3fn1]

a
**Reaction conditions:** (a) CF_3_-TMS, CsF, −78 °C, 2-4 h, 37%; (b) B_2_(OH)_4_, XphosPdG2, KOAc, EtOH, 80 °C, 1 h, 72%; (c) Pd­(dppf)­Cl_2_, KOAc, B_2_pin_2_, 1,4-dioxane, 80 °C; (d) NH_2_OH, DCM, rt, 1 h; (e) 5% TFA, DCM, 1 h, 22% over three steps.

Benzoxazaborines have recently emerged as a new drug chemotype.^28^ We have accessed the benzoxazaborine analogue **22** from bromo benzaldehyde intermediate **6** via three steps (Scheme 3). First, the borylation was performed under standard conditions to afford pinacol boronate **21**. Aldehyde **21** was treated with hydroxylamine aq. solution to afford the imine, followed by cleavage of the pinacol group under acidic conditions, allowing the in situ cyclization to afford **22**.

To investigate the regioisomeric benzoxaborole SAR, a similar synthetic route was used (Scheme 4). Bromo ester pyrimidine-4-one derivative **23** was synthesized via oxidative cyclization of **3** and methyl 2-bromo-4-formylbenzoate. The resulting intermediate **23** was then converted to pyrimidinyl amine **24** using BOP/DBU-mediated S_N_Ar with 4-fluorobenzylamine. Treatment of **24** with an excess amount of MeMgCl afforded the corresponding gem-dimethyl alcohol **25**, which was then converted into **26** by a Pd-catalyzed borylation followed by acidic workup. In addition, **24** was converted to aldehyde derivative **27** in two steps (DIBAL-H reduction followed by MnO_2_ oxidation). Aldehyde intermediate **27** was then treated with MeMgCl to afford alcohol **28**. Alcohol **28** was then converted into **29** via Miyaura borylation, followed by acidic workup. On the other hand, **30** was accessed from **27** via borylation, followed by NaBH_4_ reduction of the aldehyde.

**Scheme 4:**
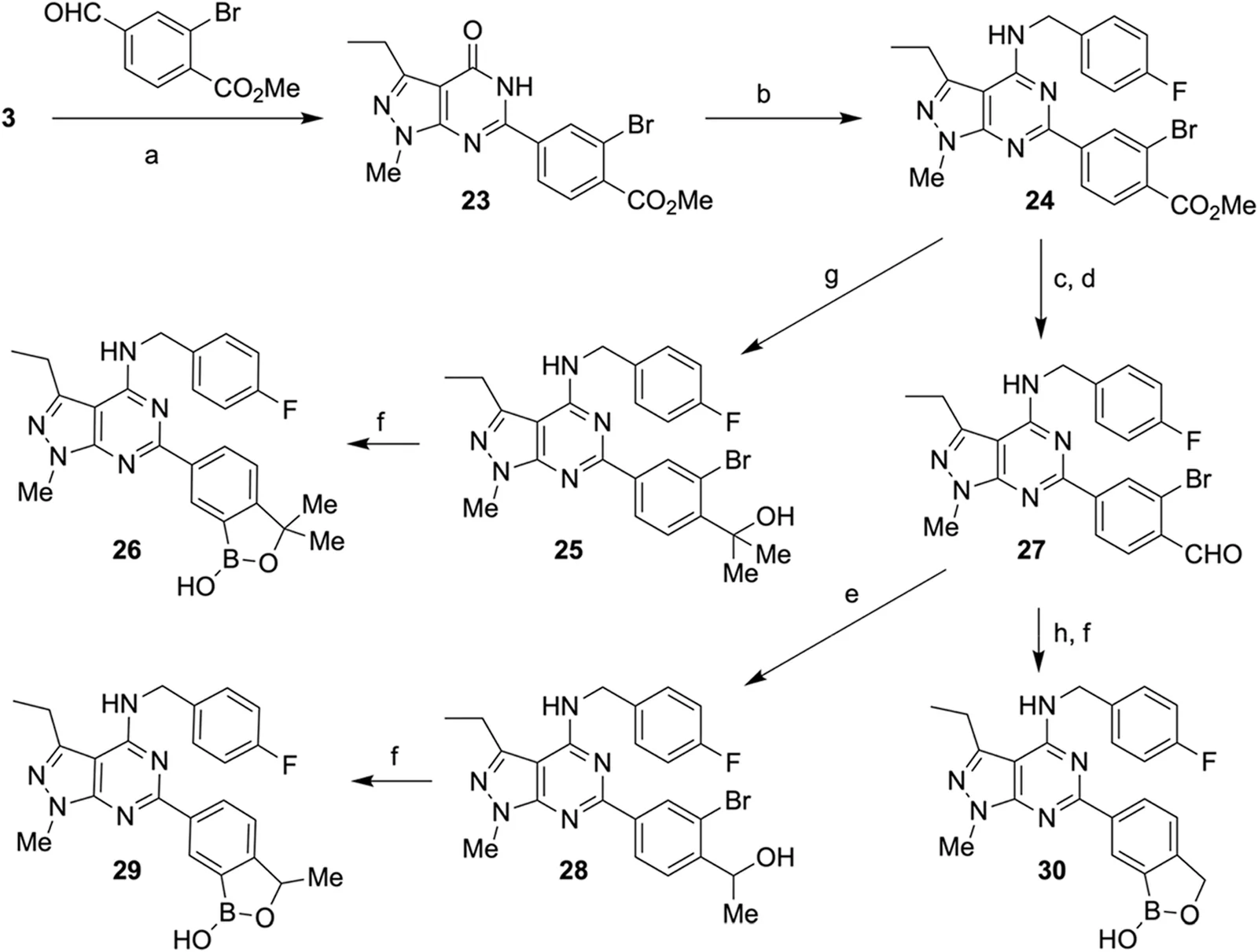
Synthesis of Isomeric Benzoxaborole Compounds[Fn s4fn1]

a
**Reaction conditions:** (a) I_2_, CH_3_CN, reflux, 16 h, 68%; (b) BOP, DBU, 4-fluorobenzylamine, DMF, rt, 16 h, 72%; (c) DIBAL-H, DCM, 0 °C, 2 h, 81%; (d) MnO_2_, DCM, rt, 2 h, 92%; (e) MeMgCl, THF, 0 °C-rt, 2 h, 54%; (f) B_2_(OH)_4_, KOAc, XphosPdG2, EtOH, 80 °C, 1 h, 74–77%; (g) MeMgCl, 0-60 °C, 4 h, 29%; (h) NaBH_4_, MeOH, 0 °C, 89%.

Next, we focused on varying the ring size of the benzoxaborole into the six-membered ring benzoxaborinol derivative **35** (Scheme 5) and seven-membered ring benzoxaboripinol derivative **39** (Scheme 6). Benzoxaborinol **35** was synthesized from aminopyrazole **3** in six steps. Oxidative cyclization of **3** with the 3-bromo-4-chlorobenzaldehyde followed by BOP/DBU-mediated S_N_Ar reaction with benzylamine afforded bromo-chloro-phenyl intermediate **32**. Bromo **32** was then subjected to a Suzuki reaction with commercially available (*E*)-2-(2-ethoxyvinyl)-4,4,5,5-tetramethyl-1,3,2-dioxaborolane to afford vinyl ether **33**. The Suzuki reaction was performed at 80 °C for 4 h to prevent reaction at the chlorine position. Next, **33** was treated with concentrated HCl to afford the aldehyde derivative **34**. Finally, reduction of the aldehyde **34** with NaBH_4_ followed by Pd-catalyzed borylation afforded benzoxaborinol **35**.

**Scheme 5:**
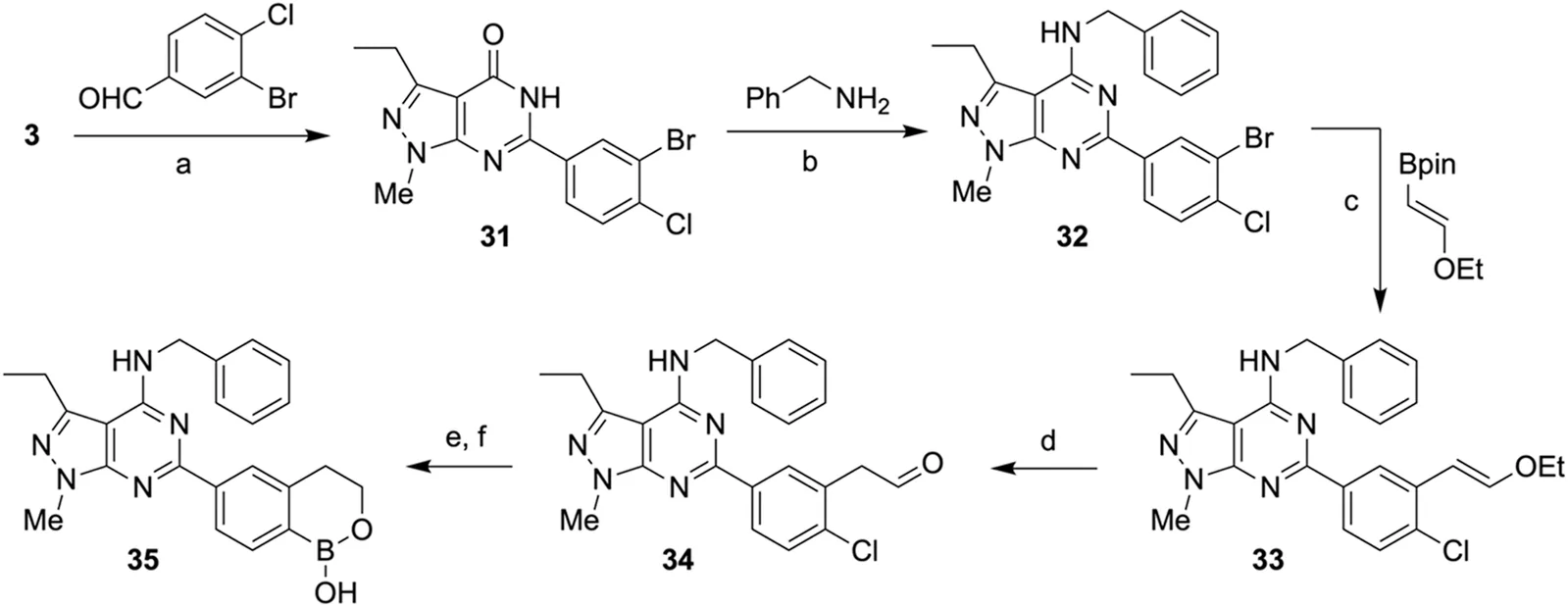
Synthesis of Benzoxaborinol **35** [Fn s5fn1]

a
**Reaction conditions:** (a) I_2_, CH_3_CN, reflux, 16 h, 70%; (b) BOP, DBU, DMF, rt, 16 h, 71%; (c) Pd­(dppf)­Cl_2_, K_2_CO_3_, 1,4-dioxane, 80 °C, 4 h, 52%; (d) Conc. HCl, MeOH, 1 h, 73%; (e) NaBH_4_, 0 °C, 20 min, 87%; (f) B_2_(OH)_4_, XphosPdG2, KOAc EtOH, 80 °C, 1 h, 71%.

**Scheme 6:**
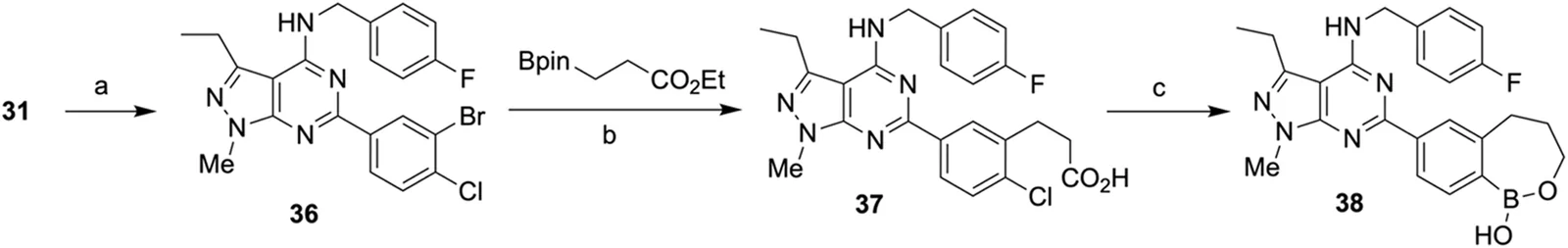
Synthesis of Benzoxaboripinol **38** [Fn s6fn1]

1
**Reaction conditions:** (a) BOP, DBU, 4-fluorobenzylamine, DMF, rt, 16 h, 67%; (b) Pd­(dppf)­Cl_2_, K_2_CO_3_, 1,4-dioxane, 80 °C, 4 h, 48%; (c) i. BH_3_-THF, 0 °C-rt, 4 h; ii. B_2_(OH)_4_, XphosPdG2, KOAc EtOH, 80 °C, 1 h, 22%

As represented in Scheme 6, benzoxaboripinol **38** was synthesized from **31** via S_N_Ar with 4-fluorobenzylamine followed by a Suzuki reaction with ethyl 3-(4,4,5,5-tetramethyl-1,3,2-dioxaborolan-2-yl)­propanoate to afford chlorophenyl propanoic acid **37**. Reduction of the carboxylic acid group with BH_3_-THF complex then afforded the corresponding alcohol. Finally, the Pd-catalyzed borylation at the Cl position followed by acidic workup afforded benzoxaboripinol **38**.

### In Vitro *Cryptosporidium parvum* Growth Assay and Structure–Activity Relationships

EC_50_ values of target analogues were measured in vitro using a previously established high-content microscopy assay that measures *C. parvum* asexual replication within the human colon carcinoma cell line HCT-8.^29^ *C. parvum* (Iowa strain) oocysts treated with sodium taurocholate to induce excystation were added to nearly confluent HCT-8 cell monolayers grown in clear-bottomed microtiter plates, after which emerging parasite sporozoites infected the HCT-8 cells. Test compounds or vehicle (DMSO) were added after allowing 3 h for host cell invasion, followed by incubation for 48 h, and then staining for parasite and host cells and enumeration by automated fluorescence microscopy. Results are shown in Tables 1 and 2.

**Table 1:**
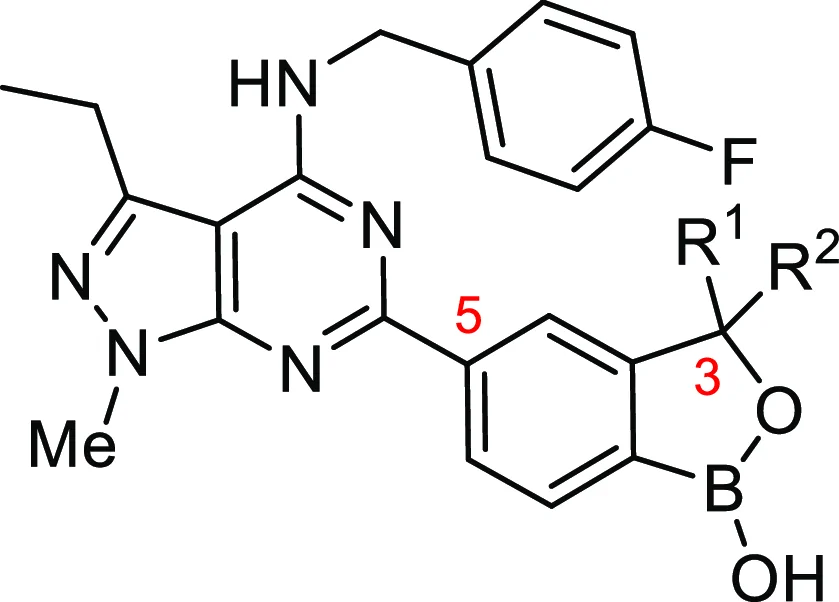
Structure–Activity Relationships of the Benzoxaborole C3 Position

**entry**	**compound**	***R*** ^**1**^	***R*** ^**2**^	***Cp*** **HCT-8 EC**_**50**_, **μM** ^a^	***Cp*** **HCT-8 EC**_**50**_ **95% confidence interval, μM** ^b^
1	**NTZ**			1.6	1.3–2.0
2	**2** (SLU-10906)	H	H	0.19	0.16–0.20
3	*rac-* **11** (SLU-11695)	Me	H	0.025	0.021–0.032
4	**12**	Et	H	0.57	0.45–0.69
5	**13**	*i*-Pr	H	3.8	3.3–4.4
6	**14**	Ph	H	2.1	1.7–2.4
7	**20**	CF_3_	H	2.0	1.8–2.3
8	**16**	Me	Me	3.2	2.7–3.7
9	*ent1-* **11**	Me	H	4.5	3.7–5.4
10	*ent2-* **11** (SLU-12091)	Me	H	0.011	0.0099–0.013

a EC_50_ values.

b 95% confidence intervals are calculated from average potency in the Cp HCT-8 assay from at least two 9-point dose−response experiments (n = 8 technical replicates per dose).

**Table 2:**
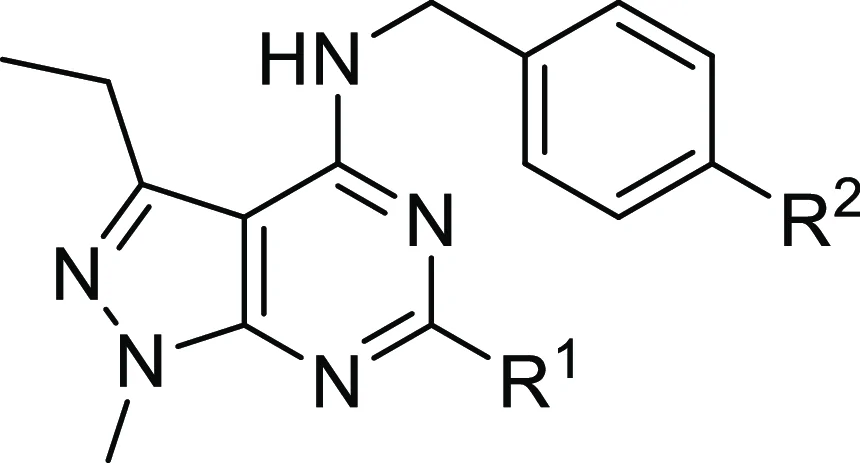

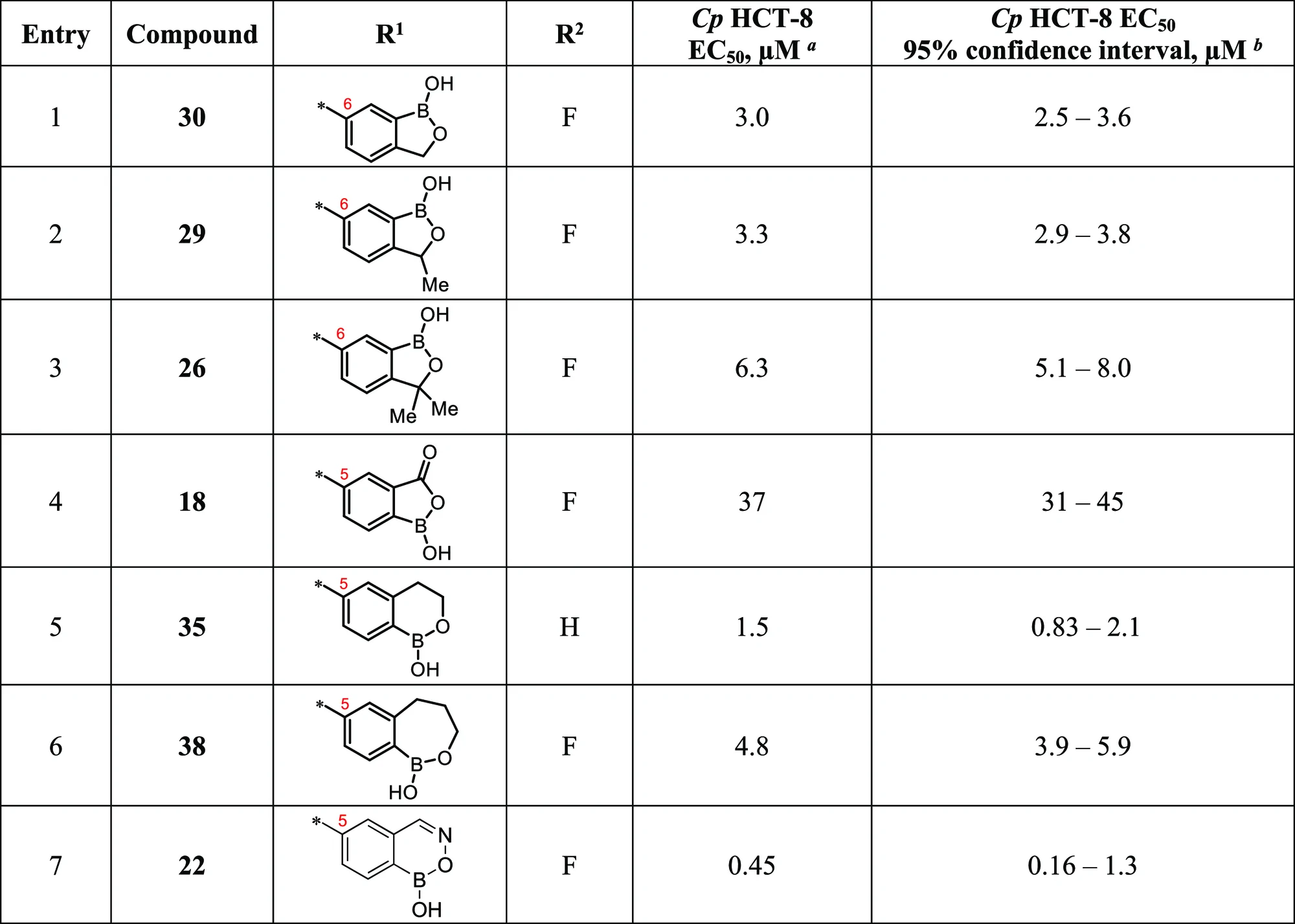
Structure–Activity Relationship Studies on the Regiochemistry and Variation of the Oxaborole Ring

a EC_50_ values and ^b^95% confidence intervals are calculated from average potency in the *Cp* HCT-8 assay from at least two 9-point dose–response experiments (*n* = 8 technical replicates per dose).

Functionalization of the C3 of the benzoxaborole revealed a significant SAR trend (Table 1). An 8-fold improvement was observed by addition of a single methyl group (*rac*-**11**, EC_50_ 0.025 μM). However, increasing the alkyl chain length to ethyl (**12**, EC_50_ 0.57 μM) or bulkier isopropyl (**13**, EC_50_ 3.8 μM) groups reduced potency, correlating with steric bulk. Additionally, replacing the alkyl groups with a phenyl group (**14**, EC_50_ 2.1 μM) resulted in potency similar to isopropyl. Furthermore, replacing the methyl group with CF_3_ group reduced potency 80-fold (**20**, EC_50_ 2.0 μM). In an effort to remove the stereogenic center in **11**, we prepared the gem-dimethyl derivative **16**; however, only moderate potency was observed (EC_50_ 3.2 μM; 128-fold loss in potency compared to *rac-* **11**).

Since benzoxaborole *rac-* **11** was exceptionally potent and gem-dimethyl **16** lost significant potency, the enantiomers of **11** were resolved via chiral HPLC using a chiral stationary phase column to give the individual enantiomers, where the first eluting enantiomer was defined as *ent1-* **11** and the second eluting enantiomer as *ent2-* **11**. *ent2-* **11** is the more active enantiomer (EC_50_ 0.011 μM, *n* = 4; EC_90_ 0.020 μM) and *ent1-* **11** is 400-fold less active (EC_50_ 4.5 μM).

Table 2 presents SAR varying the regiochemistry and the oxaborole ring itself. In the original benzoic acid series, both 3- and 4-COOH substitutions were equipotent.^24^ To test this in the benzoxaboroles, substitution at the C6 position (**30**) instead of C5 ^2^ was tested. This regioisomeric benzoxaborole resulted in >15-fold loss in potency (**30**, EC_50_ 3.0 μM) relative to **2**, which may be due to an improper trajectory for the interaction of the benzoxaborole with the active site metal ions, either in *Cp*PDE1 or another target. Further substitution at the C3 position (**29, 26;** EC _50_ of 3.3 and 6.3 μM, respectively) failed to rescue potency, consistent with an improper trajectory of the benzoxaborole ring.

A recent study revealed that benzoxaborolones can be significantly more biologically stable than benzoxaboroles.^30^ Hence, C5-substituted benzoxaborolone derivative **18** was synthesized and tested but, unfortunately, a ∼1400-fold loss of anti-*Cryptosporidium* activity was observed (EC_50_ = 37 μM) compared to *rac*-**11**.

Next, the ring size of the fused benzoxaborinol was varied. The six-membered benzoxaborinol (**35**, EC_50_ 1.54 μM) was >8-fold less potent compared to benzoxaborole **2**. It is noteworthy that a change in the substitution at the *R* ^2^ position was made in the case of **35**, but based on our observations, variation at *R* ^2^ between H and F has minimal impact on potency (unpublished data, not shown). As compound **35** was moderately active, the impact of the *R* ^2^ on potency was not explored further. On the other hand, enlargement of the six-member ring to the seven-membered benzoxaboripinol (**38**, EC_50_ 4.83 μM) further reduced potency by >25-fold.

Benzoxazaborine derivatives have been identified as promising chemical entities in medicinal chemistry with improved solubilities and stabilities. Hence, we have incorporated the benzoxazaborine onto our pyrazolopyrimidine core. The change was tolerated but led to a modest 2-fold decrease in potency (**22**, EC_50_ 0.45 μM) compared to benzoxaborole **2**.

### *In Vitro* Pharmacokinetic Profiling

In order to compare the physicochemical pharmacokinetic properties of *rac-* **11** to prior lead **2**, *rac-* **11** was profiled for aqueous solubility, membrane permeability, metabolic stability, and plasma protein binding (Jubilant Biosys Limited). Aqueous solubility was determined to be low (<2 μg/mL) in pH 7.4 PBS buffer under thermodynamic conditions, contrasting the moderate solubility of **2** of 11 μM^24^, although the latter had been determined under kinetic conditions. Membrane permeability was assessed as low-medium in a PAMPA assay (P_app_ 15.7 nm/s), significantly lower than **2** (206 nm/s),^24^ possibly underestimated due to poor sample recovery (31.9%) in the assay. Note that in vivo efficacy for *Cryptosporidium* relies on intestinal exposure^31^ and that low solubility and/or permeability may be advantageous for in vivo efficacy.^15^

Metabolic stability was determined to be stable in human liver microsomes (HLM) with a half-life of 40 min and Cl_int_ of 34 μg/min/mg. Prior assessment of **2** in mouse liver microsomes (MLM) indicated similar stability (half-life 57 min).^24^ Finally, assessment of human plasma protein binding indicated that *rac*-**11** is highly protein bound (99.7%) and is stable in human plasma (90% remaining after 4 h).

### Safety Pharmacology Profiling

Previously, we showed that **2** is highly selective for *Cryptosporidium* vs host cells and has no effect on growth of HCT-8 cells at the highest concentration tested (100 μM; >500-fold selectivity, host cell CC_50_/*C. parvum* EC_50_).^24^ Here, we found that *rac-* **11** has a CC_50_ in HCT-8 cells of ∼77 μM, >3000-fold selectivity, and no effect on growth was observed for *ent2-* **11** and all the test analogs in HCT-8 cells up to the highest concentration tested (50 μM).

Short-term use is anticipated for treating cryptosporidiosis,^32^ and numerous FDA-approved PDE inhibitors that are taken semielectively (e.g., sildenafil) or chronically (e.g., apremilast) have proven safe for indications including erectile dysfunction, pulmonary hypertension, heart failure, and inflammatory diseases like psoriatic arthritis.^33^ Nonetheless, to more broadly determine selectivity versus human PDEs, benzoxaborole *ent2-* **11** was profiled against a panel of 13 human PDEs (see Supporting Information; PDE enzyme assay panel at Eurofins; Table 3). *ent2-* **11** significantly inhibits (>50%) some but not all *h*PDEs at 10 μM.

**Table 3:** Inhibition of Human PDEs by Benzoxaborole ***ent2-*** **11**

	**% inhibition at**		
enzyme^a^	**0.1 μM**	**1 μM**	**10 μM**
PDE1B			27
PDE2A1			80
PDE3A			87
PDE3B			78
PDE4A1A			77
PDE4D2			92
PDE5	24	65	96
PDE6			–14
PDE7A1			28
PDE8A1			27
PDE10A2			18
PDE11A	–21	22	64

a PDE enzyme assays were performed as a panel at Eurofins in duplicate.

Human PDEs 5 and 11 were selected for follow-up. At 0.1 μM, potent enantiomer *ent2-* **11** inhibits *h*PDE 5 and 11 by 24 and 0%, respectively, at 0.1 μM, and by 65 and 22% at 1 μM, suggesting that focus on improvement in *h*PDE selectivity is a required but achievable goal given the numerous PDE crystal structures available for structure-based drug design and previous successes by others in designing selective PDE inhibitors.^34–36^ For proper context, it should be noted that the *Cp-*infection HCT-8 assay uses 10% fetal bovine serum, while PDE enzyme assays at Eurofins generally do not include serum (with the exceptions of 0.03% BSA for *h*PDEs 5 and 6 and 0.21% BSA for PDE1B), highlighting the superior anti-*Cryptosporidium* potency of benzoxaboroles versus inhibiting *h*PDE enzymes when taking into differences in free drug concentrations (e.g., *rac*-**11** is only 0.3% unbound in human plasma).

### Benzoxaborole has Curative Efficacy in the NSG Mouse *Cp*-Infection Model

A previously established NOD *scid* gamma (NSG) mouse model of chronic *C. parvum* infection was used to test the in vivo efficacy of the anticryptosporidial benzoxaboroles.^37^ All mouse experiments were performed in compliance with animal care guidelines and were approved by the University of Vermont’s Institutional Animal Care and Use Committee. Male NSG mice (*Mus musculus* NOD.Cg-*Prkdc* ^*scid*^ *Il2rg* ^*tm1Wjl*^/SzJ) were infected with *C. parvum* by oral gavage on experiment day 0, and the infection was allowed to progress for 7 days prior to oral administration of experimental compounds dosed for 4 days (50 mg/kg twice daily in 1% hydroxypropylmethylcellulose (HPMC)). Parasite shedding in feces was measured using a published qPCR assay.^38^ In our prior report, benzoic acid **1** (SLU-2815) and benzoxaborole **2** (SLU-10906) reduced shedding by >98% when dosed for 7 days (D7-D14), but relapse was observed 7 days post-treatment for both compounds (D21) (see publication^24^). In the present study, a shorter 4-day regimen of drug treatment was used, with **1** included for comparison. Under this 4-day regimen, **1** was inactive in the NSG mouse (Figure 5). On the other hand, just 4 days of potent *rac-* **11** (SLU-11695) (50 mg/kg twice daily) treatment eliminated *C. parvum* shedding with no evidence of relapse up to 8 days after stopping therapy (not followed further). Importantly, all mice treated with *rac*-**11** suffered no apparent side effects.

**Figure 5:**
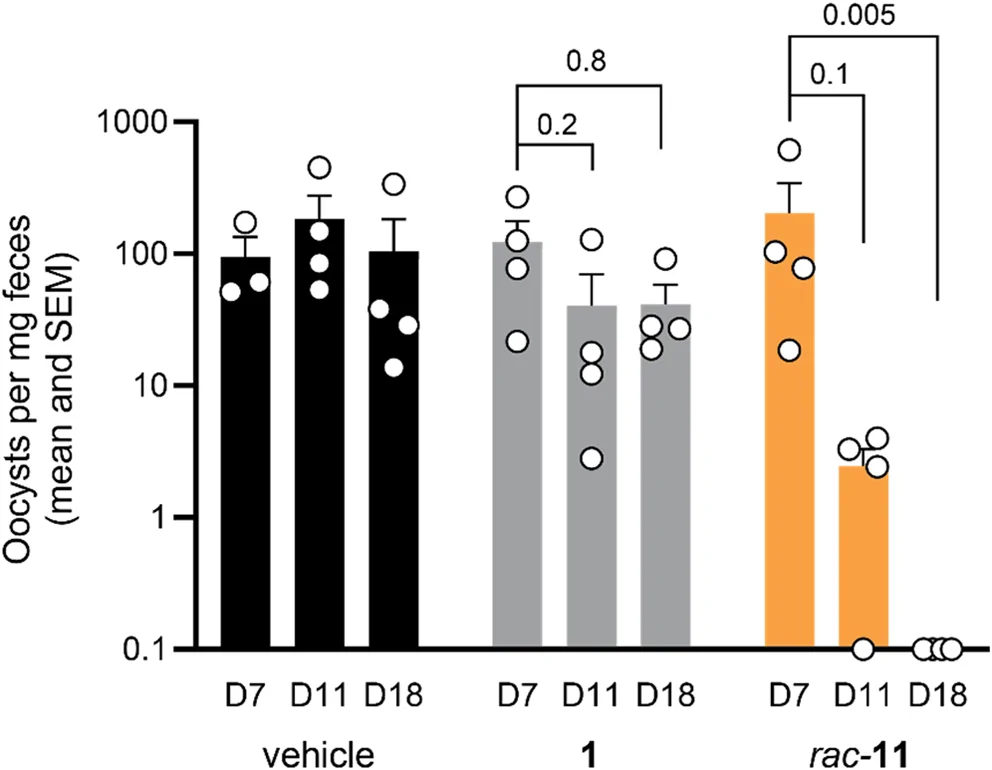
Improved *in vivo* efficacy of potent benzoxaborole inhibitor *rac-*11 (SLU-11695) in immunocompromised mice. A 4-day treatment protocol was used to compare **1** and *rac-* **11** (SLU-11695) with compounds dosed orally at 50 mg/kg twice daily on days 7, 8, 9, and 10 postinfection. Parasite shedding in feces was measured by qPCR on the indicated days postinfection, and data are the mean and SEM for parasite shedding (*n* = 4 mice per group). Data points below the limit of detection are plotted on the *x*-axis. Adjusted *p* values vs pretreatment day 7 value are as shown (nonparametric Kruskal–Wallis test with Dunn’s multiple comparisons test).

## Conclusions

Here we report on the synthesis and SAR studies around the benzoxaborole ring of compound **2** with 15 novel analogs exploring substitution on the C3 position, regioisomers, oxaborole ring expansion, and oxaborole ring replacements. *In vitro* potency was highly sensitive to the substitution and position of the benzoxaborole, with a single C3 methyl group (*rac-* **11**) being preferred by 8-, 23-, and 128-fold over hydrogen, ethyl, and gem-dimethyl, respectively. Resolution of the enantiomers of *rac-* **11** gave a single enantiomer with ∼2-fold more potency and the other enantiomer with ∼400-fold less potency, suggesting a finely tuned positioning of the C3 methyl benzoxaborole in the parasitic target. Furthermore, *ent2-* **11** (SLU-12091) is the most potent anti*-Cryptosporidium* compound we have identified to date in any series.

Unlike most cryptosporidiosis mouse models in which infection resolves spontaneously,^14,37,39,40^ NOD *scid* gamma (NSG) mice infected with *C. parvum* develop chronic intestinal infection that mimics chronic infection in immunosuppressed people and, for drug efficacy studies, enables assessment of relapse following treatment cessation.^14^ In addition to the severe combined immunodeficiency (SCID) defect, NSG mice lack known sources of IFN-γ, including the monocyte/macrophage lineage and natural killer cells.^41^ The *in vivo* efficacy of *rac-* **11** in the highly immunocompromised NSG model is consistent with previously observed rapid *in vitro* parasite killing observed with **2**.^24^ Furthermore, curative efficacy in the NSG model is consistent with the published ideal target product profile that states a goal of efficacy in severely immunocompromised people with chronic cryptosporidiosis.^32^

Further research is needed, however, to definitively determine the parasitic target of the benzoxaborole series. *Cp*PDE1 is a strong candidate, but previously reported genetic modifications of *Cp*PDE1 had no impact on the potency of **2** or *rac-* **11** (unpublished and Ajiboye et al.^23^) against *C. parvum*, raising the potential of a secondary target. As illustrated in Figure 4, a reasonable docking pose in a *Cp*PDE1 model can be obtained. We have also been able to dock **2** into the *C. hominis* CPSF3 X-ray structure 6Q55^17^, the homologous target of another antiparasitic benzoxaborole, acoziborole (unpublished). Resolution of the biological target of this pyrazolopyrimidine benzoxaborole series is needed to accelerate development of a more selective antiparasitic agent over human off-targets such as human phosphodiesterases.

The discovery of pyrazolopyrimidine benzoxaboroles *rac-* **11** and *ent2-* **11** is an important advance in the development of a novel therapeutic for cryptosporidiosis. Efforts to further improve the parasite over host selectivity, *in vivo* efficacy, and target identification will be reported in due course.

## Experimental Section

### Biological Methods

#### Antiparasitic Activity

Anticryptosporidial activity was measured using a previously described high-content microscopy assay, a human ileocecal adenocarcinoma (HCT-8) cell infection model, and *C. parvum* Iowa isolate oocysts (Bunch Grass Farms).^29^ Oocysts were treated to induce excystation (10 mM HCL (10 min, 37 °C) followed by 2 mM sodium taurocholate in PBS (10 min, 16 °C)) and used to infect nearly confluent HCT-8 cell monolayers grown in 384-well clear-bottom plates in RPMI 1640 medium with HEPES, sodium pyruvate (1 mM), and l-glutamine (ATCC) supplemented with 10% fetal bovine serum (Sigma-Aldrich) and 120 U/ml penicillin and 120 μg/mL streptomycin. Compounds were added after 3 h incubation, followed by incubation for an additional 45 h (37 °C, 5% CO_2_). Wells were then washed 3 times with PBS containing 111 mM D-galactose, fixed with 4% formaldehyde, permeabilized with 0.25% Triton X-100, blocked with 4% bovine serum albumin (BSA), and stained for fluorescence microscopy using fluorescein-labeled *Vicia villosa* lectin (Vector Laboratories) to label the parasitophorous vacuoles and Hoechst 33258 (Anaspec) to label the cell nuclei. A Nikon Eclipse Ti2000 epifluorescence microscope with an automated stage was programmed to focus on the center of each well and take a 3 × 3 composite image using an EXi blue fluorescence microscopy camera (QImaging) with a 20X objective (NA = 0.45). Nuclei and parasite images were separately exported as.tif files, and parasites and host cells were counted using published NIH ImageJ macros.^29^ Dose–response curves were plotted, and half-maximal effective concentrations (EC_50_) and 90% effective concentrations (EC_90_) were calculated using GraphPad Prism software version 10.0.2.

#### Host Cell Toxicity

Host cell cytotoxicity was assessed for all compounds with fluorescence microscopy by measuring the number of host cell nuclei simultaneously with measuring the number of parasites (see Antiparasitic activity). For prioritized compounds, host cell toxicity was also measured using HCT-8 Cells and the CellTiter AQ_ueous_ assay kit (Promega) following the manufacturer’s instructions. Increasing concentrations of compounds were added to 95% confluent HCT-8 cell monolayers, followed by incubation for 48 h (37 °C, 5% CO_2_). Cell numbers were expressed as the percent of vehicle control (DMSO). GraphPad Prism software version 10.0.2 was used to plot graphs and calculate the concentration that inhibited 50% of host cell proliferation compared to DMSO control (CC_50_).

#### Thermodynamic Aqueous Solubility

The assay was performed by Jubilant Biosys Limited. Results are an average of two experiments. Seven calibration standards (2, 5, 10, 50, 100, 250, and 500 μg/mL) were prepared in DMSO from the 1 mg/mL primary DMSO stock solution. One mg of the test compound was transferred into each of two separate centrifuge tubes (*n* = 2). To these tubes, 1 mL of PBS was added to get a final concentration of 1 mg/mL and kept for shaking at 300 rpm for 24 h at 37 °C. After completion of the incubation time, the centrifuge tubes were subjected to centrifugation at 14,000 rpm at 25 °C for 10 min to separate undissolved drug from dissolved drug. 200 μL of supernatant was collected from both tubes and filtered through a 0.45 μm syringe filter. For the control compound (CC) and unknown samples, 10 μL of supernatant of each sample was diluted in quenching solution containing the internal standard (IS; dilution factor 10×). Furthermore, 20 μL of 10× diluted sample was further diluted with 180 μL of quenching solution containing IS and submitted to LC-HRMS.

#### PAMPA Membrane Permeability

The assay was performed by Jubilant Biosys Limited. Results are an average of two experiments. A working stock (5 μM final concentration) was prepared by spiking 1.25 μL (20 mM of test compound) in 5 mL transport media (PBS buffer with 5% DMSO). A 2% solution (w/v) of lecithin in dodecane (∼500 μL/plate) was prepared and mixed to ensure complete dissolution. An aliquot of 5 μL of the Lecithin-dodecane mixture was added into each donor plate well, avoiding pipette tip contact with the membrane. Immediately after the application of the artificial membrane (within 10 min maximum), 150 μL of compound-containing donor solutions (dissolved in 5% DMSO, PBS) was added to each well of the donor plate. Subsequently, 300 μL of aqueous buffer was added to each well of the PTFE acceptor plate. The compound-filled donor plate was slowly placed onto the acceptor plate, making sure the underside of the membrane is in contact with the buffer in all wells. The lid was replaced, and the assembly was incubated at room temperature for 16 h. After incubation, the acceptor plate was analyzed for compound concentration by aspirating 100 μL aliquots from both apical and basal compartments and adding an equal volume of acetonitrile containing internal standard. *P*_app_ (cm/s) = (*V*_r_/*C*_0_) (1/*S*) (d*C*/d*t*)

Notes: *P*_app_ = apparent permeability, *V*_r_ = volume of medium in the receiver chamber, *C*_0_ = initial peak area ratio of the test drug, *S* = surface area of monolayer, d*C*/d*t* = drug PAR in the receiver chamber with time. Reference standard (dexamethasone) final concentration used: 5 μM. Both apical and basal pHs are 7.4.

#### Human Microsomal Stability (HLM)

The assay was performed by Jubilant Biosys Limited. The experiment was performed in duplicate. 10 μL Human liver microsomes (Xenotech; 20 mg/mL) were suspended in 346 μL of 66.7 mM potassium phosphate buffer (pH 7.4) to get 0.5 mg/mL of microsomal protein in the wells labeled as incubation mixture. 4 μL of test compound or control from 100 μM intermediate stock (prepared in ACN: Water) was added to the above incubation mixture to give a working concentration of 1 μM. All the samples were preincubated for 10 min at 37 °C in a shaking water bath. 45 μL of the above aliquot was transferred, followed by the addition of 5 μL of buffer from the incubation mixture to wells labeled as controls and incubated for 30 min. At the end of the control incubation period, the samples were quenched with 300 μL of acetonitrile containing internal standard. 35 μL of 10 mM NADPH was added to the remaining incubation mixture (315 μL) to initiate the reaction. At the end of each incubation period (0, 5, 15, and 30 min) of respective samples, 50 μL of the microsomal mixture was quenched with 300 μL of acetonitrile containing internal standard. The resulting samples were centrifuged at 4000 rpm for 20 min, and after centrifugation, the samples were diluted with water in a 1:1 ratio. Diluted samples (200 μL) were taken for LC-MS/MS analysis.

#### Human Plasma Protein Binding (PPB)

The assay was performed by Jubilant Biosys Limited. Results are an average of two experiments. A final concentration of 3 μM was prepared by spiking 1 μL of a 3 mM working stock solution in 999 μL of 100% blank plasma and mixed thoroughly. The rapid equilibrium dialysis base plate with inserts was prepared following the manufacturer’s instructions. Each donor chamber was filled with 200 μL of plasma (in triplicate) and dialyzed against 350 μL of PBS (phosphate-buffered saline) in the receiver chamber. The dialysis plate was sealed and placed in an incubator at 37 ± 1 °C with 5% CO_2_ for 4 h with constant shaking at 450 rpm. After equilibration time, 50 μL of plasma was taken out from the donor chamber and added to 50 μL of blank phosphate buffer and quenched with 300 μL of cold acetonitrile containing internal standard (IS). Similarly, 50 μL of PBS was taken from the receiver chamber, added to 50 μL of blank plasma, and quenched with 300 μL of cold acetonitrile containing IS. For *T*_0_ hr and *T*_4_ hr samples, 50 μL of plasma containing control/test compound (3 μM final concentration) was aliquoted at the respective time point and then 50 μL of blank phosphate buffer was added and quenched with 300 μL of cold acetonitrile containing IS. For the blank sample, 50 μL of blank plasma/buffer was added to 50 μL of blank phosphate buffer/plasma and then quenched with 300 μL of cold acetonitrile. Naltrexone and warfarin were used as positive controls.

Data analysis

*F*_u_ measured = (peak area ratio in the buffer side)/(peak area ratio in the plasma side).

Peak area ratio: compound peak area/internal standard peak area

% Unbound = Fu × 100.

% Bound = 100 – % unbound

% Recovery = ((peak area ratio in buffer side × volume in buffer side) + (peak area ratio in plasma side × volume in plasma side))/(peak area ratio at *T*_0_ sample × volume in plasma side) × 100 where *T*_0_ is the 0 h time point sample; volume in buffer side = 350 μL; volume in plasma side = 200 μL.

% stability = (peak area ratio at *T*_4_ sample)/(peak area ratio at *T*_0_ sample) × 100 where *T*_0_ is the 0 h time point sample and *T*_4_ is the 4 h time point sample.

#### *In Vitro* Human PDE Assays

Human PDE inhibition was assayed by Eurofins according to published methods.^42–44^ Recombinant enzyme expressed in Sf9 cells (PDE1B, PDE2A1, PDE3A, PDE3B, PDE4A1A, PDE4B1, PDE4D2, PDE7A1, PDE8A1, PDE10A2, PDE11A) or purified (PDE5 (human platelets), PDE6 (bovine retina)) was used to measure hydrolysis of [3H]­cGMP or [3H]­cAMP as appropriate for each enzyme, followed by measurement of [3H]­5′GMP or [3H]­5′AMP by scintillation counting. Reference compounds for each enzyme or ***ent2*** **-11** were dissolved in DMSO. Results were expressed as percent of control specific activity ((measured specific activity/control specific activity) × 100) and as a percent inhibition of control specific activity (100-((measured specific activity/control specific activity) × 100)).

#### *In Vivo* NSG Mouse Assay

All mouse efficacy studies were performed at the University of Vermont in compliance with animal care guidelines and were approved by the University of Vermont Institutional Animal Care and Use Committee (Protocol 202000153). Male normal flora NOD SCID γ mice (NOD.Cg-*Prkdc* ^*scid*^ *IL2rg* ^*tm1Wjl*^/SzJ) were purchased from Jackson Laboratory (Maine, USA) and housed for 1 week for acclimatization. Mice were then infected by oral gavage at 4–5 weeks of age with 10^5^ *C. parvum* Iowa isolate oocysts (*n* = 4 mice per experimental group). Mice were treated by oral gavage twice daily (50 mg/kg) with the indicated compounds on days 7, 8, 9, and 10 following infection, and thereafter monitored for relapse until day 18 (8 days after treatment cessation). Previously prepared aliquots of compound in DMSO were thawed on the day of administration, thoroughly mixed, and diluted in 1% hydoxymethylcellulose/PBS to a total volume of 100 μL and final concentration of 5% DMSO. Oocyst shedding in feces was measured using a previously validated qPCR assay.^38^

### Chemical Methods

#### Safety Statement

Although there were no unexpected or new hazards identified for the synthetic methods described herein, a few hazardous reagents were employed that should be handled appropriately. DIBAL-H is a flammable and corrosive liquid (GHS: H225, H250, H314, and H318). Grignard reagents are highly flammable liquids and vapors (GHS: H225, H260, H314, H318, H335, H336, and H351). Trimethyl­(trifluoromethyl)­silane is a highly flammable liquid and vapor and, in contact with water, releases flammable gas (GHS: H225 and H261). Borane tetrahydrofuran complex solution is a highly flammable liquid and vapor and, in contact with water, releases flammable gas (GHS: H225, H260, H302, H318, H335, H336, and H351).

##### General

Commercially available reagents and solvents were used without further purification unless stated otherwise. HPLC and LCMS analyses were performed on an Agilent 1260 HPLC/MSD electrospray mass spectrometer equipped with a DAD detector (210 and 254 nm) in positive ion mode, with a scan range of 100–1000 Da. Preparative normal-phase chromatography was performed on a CombiFlash Rf+ (Teledyne Isco) with SiliaFlash F_60_ (230–400 mesh) silica gel (SiliCycle Inc.). Preparative reversed-phase HPLC was performed on an ACCQPrep HP150 (Teledyne Isco) equipped with a 30 × 250 mm C18 or 20 × 250 mm C18 RediSep Prep HPLC column with water/acetonitrile/0.1% formic acid gradient. All the NMR spectra were recorded on a Bruker 400 MHz spectrometer. The signal of the deuterated solvent was used as the internal reference. Chemical shifts (δ) are given in ppm and are referenced to a residual, not fully deuterated solvent signal. Coupling constants (*J*) are given in Hertz (Hz). HRMS spectra were recorded on an ABSciex 5600+ instrument. All final compounds were purified to ≥ 95% as determined by HPLC-UV absorbance unless noted otherwise.

### Synthesis of Synthetic Intermediates

#### 5-Amino-3-ethyl-1-methyl-1*H*-pyrazole-4-carboxamide (3)

Using a previously reported method,^24^ **3** was synthesized from malononitrile in 2 steps. ^**1**^ **H NMR:** (400 MHz, DMSO) δ 6.45 (s, 2H), 6.11 (s, 2H), 3.44 (s, 3H), 2.62 (t, *J* = 7.5 Hz, 2H), 1.11 (t, *J* = 7.5 Hz, 3H); **LCMS:** *m*/*z* (M + H)^+^ = 169.

#### Methyl 2-Bromo-5-(3-ethyl-4-hydroxy-1-methyl-1*H*-pyrazolo­[3,4-*d*]­pyrimidin-6-yl)­benzoate (4)

To a stirred solution of **3** (1.68 g, 10.0 mmol) and methyl 2-bromo-5-formylbenzoate (2.66 g, 11.0 mmol) in dry DMSO (10 mL) was added iodine (3.79 g, 15.0 mmol), and the resulting mixture was stirred at 100 °C for 16 h. After completion, the reaction mixture was filtered and washed with EtOAc to afford **4** as a white solid (2.37 g, 61%). ^**1**^ **H NMR:** (400 MHz, DMSO) δ 12.44 (s, 1H), 8.50 (s, 1H), 8.25 (d, *J* = 8.2 Hz, 1H), 7.91 (d, *J* = 8.2 Hz, 1H), 3.90 (s, 6H), 2.84 (q, *J* = 7.5 Hz, 2H), 1.27 (t, *J* = 7.5 Hz, 3H); **LCMS:** *m*/*z* (M + H)^+^ = 392.

#### Methyl 2-Bromo-4-(3-ethyl-4-hydroxy-1-methyl-1*H*-pyrazolo­[3,4-*d*]­pyrimidin-6-yl)­benzoate (23)

To a stirred solution of **3** (1.68 g, 10.0 mmol) and methyl 2-bromo-4-formylbenzoate (2.66 g, 11.0 mmol) in dry DMSO (10 mL) was added iodine (3.79 g, 15.0 mmol), and the resulting mixture was stirred at 100 °C for 16 h. After completion, the reaction mixture was filtered and washed with EtOAc to afford **23** as a white solid (2.65 g, 68%). ^**1**^ **H NMR:** (400 MHz, DMSO) δ 12.44 (s, 1H), 8.51 (d, *J* = 1.6 Hz, 1H), 8.25 (dd, *J* = 8.2, 1.7 Hz, 1H), 7.92 (d, *J* = 8.2 Hz, 1H), 3.91 (s, 6H), 2.85 (q, *J* = 7.5 Hz, 2H), 1.28 (t, *J* = 7.5 Hz, 3H); **LCMS:** *m*/*z* (M + H)^+^ = 392.

#### 6-(3-Bromo-4-chlorophenyl)-3-ethyl-1-methyl-1*H*-pyrazolo­[3,4-*d*]­pyrimidin-4-ol (31)

To a stirred solution of **3** (840 mg, 5.0 mmol) and methyl 3-bromo-4-chlorobenzaldehyde (1.2 g, 5.5 mmol) in dry DMSO (5 mL) was added iodine (1.9 g, 7.5 mmol), and the resulting mixture was stirred at 100 °C for 16 h. After completion, the reaction mixture was filtered and washed with EtOAc to afford **23** as a white solid (1.28 g, 70%). ^**1**^ **H NMR:** (400 MHz, DMSO) δ 12.36 (s, 1H), 8.55 (s, 1H), 8.18 (d, *J* = 8.1 Hz, 1H), 7.82 (d, *J* = 8.4 Hz, 1H), 3.89 (s, 3H), 2.84 (q, *J* = 7.4 Hz, 2H), 1.27 (t, *J* = 7.5 Hz, 3H); **LCMS:** *m*/*z* (M + H)^+^ = 368.

#### Methyl 2-Bromo-5-(3-ethyl-4-((4-fluorobenzyl)­amino)-1-methyl-1*H*-pyrazolo­[3,4-*d*]­pyrimidin-6-yl)­benzoate (5)

To a stirred solution of **4** (390 mg, 1.0 mmol), (4-fluorophenyl)­methanamine (138 mg, 1.1 mmol), and BOP (574 mg, 1.3 mmol) in dry DMF (2 mL) was added DBU (0.24 mL, 1.6 mmol), and the resulting mixture was stirred for 16 h at room temperature under an inert atmosphere. After completion, the reaction mixture was diluted with EtOAc (10 mL) and washed with water (2 × 5 mL). The organic layer was then dried over anhydrous Na_2_SO_4_ and concentrated under reduced pressure to afford the crude. The crude was then purified by normal-phase chromatography (10–70% EtOAc/Hexane) to afford the pure product as a light brown solid (388 mg, 78%). ^**1**^ **H NMR:** (400 MHz, DMSO) δ 8.59 (d, *J* = 1.5 Hz, 1H), 8.42 (dd, *J* = 8.1, 1.5 Hz, 1H), 7.97 (s, 1H), 7.91–7.81 (m, 1H), 7.49 (dd, *J* = 8.6, 5.7 Hz, 2H), 7.24–7.06 (m, 2H), 4.82 (d, *J* = 5.0 Hz, 2H), 3.90 (d, *J* = 9.5 Hz, 6H), 3.32 (s, 7H), 3.05 (q, *J* = 7.4 Hz, 2H), 1.29 (t, *J* = 7.5 Hz, 3H)**;** ^**19**^ **F NMR:** (377 MHz, DMSO) δ −116.32 (s); **LCMS:** *m*/*z* (M + H)^+^ = 499.

#### Methyl 2-Bromo-4-(3-ethyl-4-((4-fluorobenzyl)­amino)-1-methyl-1H-pyrazolo­[3,4-*d*]­pyrimidin-6-yl)­benzoate (24)

To a stirred solution of **23** (390 mg, 1.0 mmol), (4-fluorophenyl)­methanamine (138 mg, 1.1 mmol) and BOP (574 mg, 1.3 mmol) in dry DMF (2 mL) was added DBU (0.24 mL, 1.6 mmol), and the resulting mixture was stirred for 16 h at room temperature. After completion, the reaction mixture was diluted with EtOAc (10 mL) and washed with water (2 × 5 mL). The organic layer was then dried over anhydrous Na_2_SO_4_ and concentrated under reduced pressure to afford the crude. The crude was then purified by normal-phase chromatography (10–70% EtOAc/hexane) to afford the pure product as a light brown solid (403.6 mg, 72%). ^**1**^ **H NMR:** (400 MHz, DMSO) δ 8.59 (s, 1H), 8.42 (d, *J* = 8.1 Hz, 1H), 8.04–7.78 (m, 2H), 7.49 (dd, *J* = 8.5, 5.7 Hz, 2H), 7.15 (t, *J* = 8.9 Hz, 2H), 4.82 (d, *J* = 5.7 Hz, 2H), 3.90 (d, *J* = 8.0 Hz, 6H), 3.05 (q, *J* = 7.4 Hz, 2H), 1.29 (t, *J* = 7.5 Hz, 3H); **LCMS:** *m*/*z* (M + H)^+^ = 499.

#### 6-(3-Bromo-4-chlorophenyl)-3-ethyl-N-(4-fluorobenzyl)-1-methyl-1H-pyrazolo­[3,4-*d*]­pyrimidin-4-amine (36)

To a stirred solution of **31** (368 mg, 1.0 mmol), (4-fluorophenyl)­methanamine (138 mg, 1.1 mmol), and BOP (574 mg, 1.3 mmol) in dry DMF (2 mL) was added DBU (0.24 mL, 1.6 mmol), and the resulting mixture was stirred for 16 h at room temperature. After completion, the reaction mixture was diluted with EtOAc (10 mL) and washed with water (2 × 5 mL). The organic layer was then dried over anhydrous Na_2_SO_4_ and concentrated under reduced pressure to afford the crude. The crude was then purified by normal-phase chromatography (10–70% EtOAc/Hexane) to afford the pure product as a light brown solid (318 mg, 67%). ^**1**^ **H NMR:** (400 MHz, DMSO) δ 8.58 (t, *J* = 1.7 Hz, 1H), 8.33 (dt, *J* = 8.4, 1.9 Hz, 1H), 7.91 (t, *J* = 5.9 Hz, 1H), 7.70 (dd, *J* = 8.4, 2.2 Hz, 1H), 7.55–7.40 (m, 2H), 7.20–7.09 (m, 2H), 4.80 (d, *J* = 5.2 Hz, 2H), 3.89 (s, 3H), 3.03 (q, *J* = 7.5 Hz, 2H), 1.27 (t, *J* = 7.5 Hz, 3H); ^**19**^ **F NMR:** (377 MHz, DMSO) δ −116.35 (s); **LCMS:** *m*/*z* (M + H)^+^ = 475.

#### N-Benzyl-6-(3-bromo-4-chlorophenyl)-3-ethyl-1-methyl-1H-pyrazolo­[3,4-*d*]­pyrimidin-4-amine (32)

To a stirred solution of **31** (368 mg, 1.0 mmol), benzylamine (118 mg, 1.1 mmol), and BOP (574 mg, 1.3 mmol) in dry DMF (2 mL) was added DBU (0.24 mL, 1.6 mmol), and the resulting mixture was stirred for 16 h at room temperature. After completion, the reaction mixture was diluted with EtOAc (10 mL) and washed with water (2 × 5 mL). The organic layer was then dried over anhydrous Na_2_SO_4_ and concentrated under reduced pressure to afford the crude. The crude was then purified by normal-phase chromatography (10–70% EtOAc/Hexane) to afford the pure product as a light brown solid (324.3 mg, 71%). ^**1**^ **H NMR:** (400 MHz, DMSO) δ 8.60 (t, *J* = 1.9 Hz, 1H), 8.33 (dt, *J* = 8.5, 2.2 Hz, 1H), 7.93 (t, *J* = 5.3 Hz, 1H), 7.70 (dd, *J* = 8.4, 3.1 Hz, 1H), 7.48–7.40 (m, 2H), 7.33 (dd, *J* = 10.4, 4.8 Hz, 2H), 7.21 (t, *J* = 7.3 Hz, 1H), 4.83 (d, *J* = 5.8 Hz, 2H), 3.89 (d, *J* = 1.3 Hz, 3H), 3.04 (q, *J* = 7.4 Hz, 2H), 1.28 (t, *J* = 7.5 Hz, 3H); **LCMS:** *m*/*z* (M + H)^+^ = 457.

#### 2-Bromo-5-(3-ethyl-4-((4-fluorobenzyl)­amino)-1-methyl-1*H*-pyrazolo­[3,4-*d*]­pyrimidin-6-yl)­benzaldehyde (6)

To a stirred solution of **5** (498 mg, 1.0 mmol) in DCM (5 mL) was added DIBAL-H (2 mL, 1.0 M in DCM, 2.0 mmol) at 0 °C under an inert atmosphere. The resulting mixture was stirred at 0 °C for 2 h and then quenched with the addition of saturated NH_4_Cl solution. The organic layer was diluted with DCM (10 mL) and washed with brine (5 mL). It was then concentrated and dried under vacuum to afford **6** (386 mg, crude yield 82%). It was then redissolved in DCM (5 mL), MnO_2_ (435 mg, 5.0 mmol) was added, and stirred under inert atmosphere for 2 h at room temperature. The solids were then removed by passing through a short Celite pad and washed with DCM. The organic layer was then concentrated under reduced pressure to afford the title product as a white solid. It was taken to the next step without purification (357 mg, 93% crude yield). ^**1**^ **H NMR:** (400 MHz, DMSO) δ 10.27 (s, 1H), 8.78 (dd, *J* = 5.8, 2.1 Hz, 1H), 8.56–8.40 (m, 1H), 7.99–7.77 (m, 2H), 7.51 (dd, *J* = 8.4, 5.7 Hz, 2H), 7.13 (t, *J* = 8.9 Hz, 2H), 4.81 (d, *J* = 5.2 Hz, 2H), 3.89 (s, 3H), 3.02 (q, *J* = 7.4 Hz, 2H), 1.27 (t, *J* = 7.5 Hz, 3H); **LCMS:** *m*/*z* (M + H)^+^ = 469.

#### 2-Bromo-4-(3-ethyl-4-((4-fluorobenzyl)­amino)-1-methyl-1H-pyrazolo­[3,4-*d*]­pyrimidin-6-yl)­benzaldehyde (27)

To a stirred solution of **5** (498 mg, 1.0 mmol) in DCM (5 mL) was added DIBAL-H (2.0 mL, 1 M in DCM, 2.0 mmol) at 0 °C under inert atmosphere. The resulting mixture was stirred at 0 °C for 2 h and then quenched with the addition of saturated NH_4_Cl solution. The organic layer was diluted with DCM (10 mL) and washed with brine (5 mL). It was then concentrated and dried under vacuum to afford the crude **27** (381 mg, crude yield 81%). It was then redissolved in DCM (5 mL), MnO_2_ (435 mg, 5.0 mmol) was added, and stirred under inert atmosphere for 2 h at room temperature. The solids were then removed by passing through a short Celite pad and washed with DCM. The organic layer was then concentrated under reduced pressure to afford the title product as a white solid. It was taken to the next step without purification (361 mg, crude yield 90%). ^**1**^ **H NMR:** (400 MHz, DMSO) δ 10.26 (s, 1H), 8.61 (d, *J* = 1.4 Hz, 1H), 8.48 (d, *J* = 8.8 Hz, 1H), 7.99 (t, *J* = 5.9 Hz, 1H), 7.94 (d, *J* = 8.1 Hz, 1H), 7.49 (dd, *J* = 8.7, 5.6 Hz, 2H), 7.22–7.09 (m, 2H), 4.82 (d, *J* = 5.8 Hz, 2H), 3.92 (s, 3H), 3.05 (q, *J* = 7.5 Hz, 2H), 1.28 (t, *J* = 7.5 Hz, 3H); ^**19**^ **F NMR:** (377 MHz, DMSO) δ −116.32 (s); **LCMS:** *m*/*z* (M + H)^+^ = 469.

#### General Procedure A for the Synthesis of 7–10, 28

To a stirred solution of **6** (0.1 mmol) in dry THF was added the corresponding Grignard reagent (0.12 mmol) at 0 °C under inert atmosphere. The resulting mixture was stirred at 0 °C for 2–3 h and then quenched with the addition of saturated NH_4_Cl solution. It was then extracted with EtOAc (2 × 10 mL) and washed with brine (5 mL). The combined organic layer was then dried over anhydrous MgSO_4_ and concentrated under reduced pressure to afford the crude. The crude was then purified by chromatography (0–60% EtOAc/Hexane) to afford the pure product.

#### 1-(2-Bromo-5-(3-ethyl-4-((4-fluorobenzyl)­amino)-1-methyl-1*H-*pyrazolo­[3,4-*d*]­pyrimidin-6-yl)­phenyl)­ethan-1-ol (7)

Following the general procedure A, **6** (46.8 mg, 0.1 mmol) and MeMgCl (0.12 mL, 1 M in THF, 0.12 mmol) were reacted to afford the title compound (25.2 mg, 52%). ^**1**^ **H NMR:** (400 MHz, DMSO) δ 8.36 (d, *J* = 1.6 Hz, 1H), 8.27 (dd, *J* = 8.1, 1.5 Hz, 1H), 7.81 (t, *J* = 5.9 Hz, 1H), 7.61 (d, *J* = 8.2 Hz, 1H), 7.42 (dd, *J* = 8.7, 5.6 Hz, 2H), 7.08 (ddd, *J* = 8.9, 5.9, 2.6 Hz, 2H), 5.40 (d, *J* = 4.1 Hz, 1H), 4.97–4.84 (m, 1H), 4.74 (d, *J* = 5.8 Hz, 2H), 3.82 (s, 3H), 2.96 (q, *J* = 7.5 Hz, 2H), 1.25 (d, *J* = 6.4 Hz, 3H), 1.21 (t, *J* = 7.5 Hz, 3H); ^**19**^ **F NMR:** (377 MHz, DMSO) δ −116.4 (s); **LCMS:** *m*/*z* (M + H)^+^ = 485.

#### 1-(2-Bromo-4-(3-ethyl-4-((4-fluorobenzyl)­amino)-1-methyl-1H-pyrazolo­[3,4-*d*]­pyrimidin-6-yl)­phenyl)­ethan-1-ol (28)

Following the general procedure A, **27** (47 mg, 0.1 mmol) and MeMgCl (0.12 mL, 1 M in THF, 0.12 mmol) were reacted to afford the title compound (26.2 mg, 54%). ^**1**^ **H NMR:** (400 MHz, DMSO) δ 8.43 (d, *J* = 1.6 Hz, 1H), 8.34 (dd, *J* = 8.1, 1.5 Hz, 1H), 7.87 (t, *J* = 5.9 Hz, 1H), 7.68 (d, *J* = 8.2 Hz, 1H), 7.54–7.43 (m, 2H), 7.19–7.09 (m, 2H), 5.06–4.91 (m, 1H), 4.80 (d, *J* = 5.8 Hz, 2H), 3.89 (s, 3H), 3.03 (q, *J* = 7.4 Hz, 2H), 1.32 (d, *J* = 6.3 Hz, 3H), 1.27 (t, *J* = 7.5 Hz, 3H); ^**19**^ **F NMR:** (377 MHz, DMSO) δ −116.39 (s); **LCMS:** *m*/*z* (M + H)^+^ = 485.

#### 1-(2-Bromo-5-(3-ethyl-4-((4-fluorobenzyl)­amino)-1-methyl-1*H*-pyrazolo­[3,4-*d*]­pyrimidin-6-yl)­phenyl)­propan-1-ol (8)

Following the general procedure A, **6** (47 mg, 0.1 mmol) and EtMgBr (1 M in THF, 1.2 mmol) were reacted to afford the title compound (24.4 mg, 49%). ^**1**^ **H NMR:** (400 MHz, DMSO) δ 8.64 (d, *J* = 1.6 Hz, 1H), 8.14 (dd, *J* = 8.4, 1.8 Hz, 1H), 7.87 (t, *J* = 5.9 Hz, 1H), 7.62 (d, *J* = 8.4 Hz, 1H), 7.53 (dd, *J* = 8.0, 5.8 Hz, 2H), 7.11 (t, *J* = 8.8 Hz, 2H), 5.50 (d, *J* = 3.8 Hz, 1H), 4.80 (d, *J* = 5.7 Hz, 3H), 3.89 (s, 3H), 3.03 (q, *J* = 7.4 Hz, 2H), 1.82–1.62 (m, 1H), 1.64–1.46 (m, 1H), 1.27 (t, *J* = 7.4 Hz, 3H), 0.95 (t, *J* = 7.2 Hz, 3H); ^**19**^ **F NMR:** (377 MHz, DMSO) δ −116.34 (s); **LCMS:** *m*/*z* (M + H)^+^ = 499.

#### 1-(2-Bromo-5-(3-ethyl-4-((4-fluorobenzyl)­amino)-1-methyl-1H-pyrazolo­[3,4-*d*]­pyrimidin-6-yl)­phenyl)-2-methylpropan-1-ol (9)

Following the general procedure A, **6** (47 mg, 0.1 mmol) and *i-*PrMgBr (1 M in THF, 0.12 mmol) were reacted to afford the title compound (24.6 mg, 48%). ^**1**^ **H NMR:** (400 MHz, DMSO) δ 8.58 (dd, *J* = 5.6, 2.2 Hz, 1H), 8.14 (dd, *J* = 8.4, 2.2 Hz, 1H), 7.88 (dd, *J* = 11.6, 5.8 Hz, 1H), 7.63 (d, *J* = 8.4 Hz, 1H), 7.55–7.51 (m, 2H), 7.15–7.07 (m, 2H), 5.44 (d, *J* = 4.2 Hz, 1H), 4.86–4.76 (m, 2H), 3.89 (d, *J* = 2.3 Hz, 3H), 3.07–3.00 (m, 2H), 1.90 (dq, *J* = 13.6, 6.9 Hz, 1H), 1.28 (d, *J* = 7.5 Hz, 3H), 0.90 (dd, *J* = 15.8, 6.8 Hz, 5H); ^**19**^ **F NMR:** (377 MHz, DMSO) δ −116.36 (s); **LCMS:** *m*/*z* (M + H)^+^ = 513.

#### (2-Bromo-5-(3-ethyl-4-((4-fluorobenzyl)­amino)-1-methyl-1H-pyrazolo­[3,4-*d*]­pyrimidin-6-yl)­phenyl)­(phenyl)­methanol (10)

Following the general procedure A, **6** (47 mg, 0.1 mmol) and PhMgBr (1 M in THF, 0.12 mmol) were reacted to afford the title compound (30 mg, 55%). ^**1**^ **H NMR:** (400 MHz, DMSO) δ 8.75 (d, *J* = 2.1 Hz, 1H), 8.18 (dd, *J* = 8.4, 2.2 Hz, 1H), 7.87 (t, *J* = 6.0 Hz, 1H), 7.65 (d, *J* = 8.4 Hz, 1H), 7.50 (dd, *J* = 8.5, 5.7 Hz, 2H), 7.36 (d, *J* = 7.3 Hz, 2H), 7.30 (t, *J* = 7.4 Hz, 2H), 7.23 (dd, *J* = 8.3, 6.0 Hz, 1H), 7.08 (t, *J* = 8.9 Hz, 2H), 6.21 (d, *J* = 4.0 Hz, 1H), 6.02 (d, *J* = 3.6 Hz, 1H), 4.78 (qd, *J* = 14.8, 6.0 Hz, 2H), 3.88 (s, 3H), 3.02 (q, *J* = 7.4 Hz, 2H), 1.27 (t, *J* = 7.5 Hz, 3H); ^**19**^ **F NMR:** (377 MHz, DMSO) δ −116.33 (s); **LCMS:** *m*/*z* (M + H)^+^ = 547.

#### 2-(2-Bromo-5-(3-ethyl-4-((4-fluorobenzyl)­amino)-1-methyl-1H-pyrazolo­[3,4-*d*]­pyrimidin-6-yl)­phenyl)­propan-2-ol (15)

To a stirred solution of **5** (100 mg, 0.2 mmol) in dry THF was added MeMgCl (0.5 mmol) at 0 °C under inert atmosphere. The resulting mixture was stirred at 60 °C for 4 h and then quenched with the addition of saturated NH_4_Cl solution under ice. It was then extracted with EtOAc (2 × 10 mL) and washed with brine (5 mL). The combined organic layer was then dried over anhydrous MgSO_4_ and concentrated under reduced pressure to afford the crude. The crude was then purified by chromatography (0–60% EtOAc/Hexane) to afford the pure product (30.9 mg, 31%). ^**1**^ **H NMR:** (400 MHz, DMSO) δ 8.27 (dd, *J* = 8.3, 1.8 Hz, 1H), 8.07–7.77 (m, 2H), 7.48 (dd, *J* = 8.7, 5.6 Hz, 2H), 7.20–7.06 (m, 2H), 4.80 (d, *J* = 5.3 Hz, 2H), 3.89 (s, 3H), 3.03 (q, *J* = 7.5 Hz, 2H), 1.64 (s, 6H), 1.28 (t, *J* = 7.5 Hz, 3H); ^**19**^ **F NMR:** (377 MHz, DMSO) δ −116.40 (s); **LCMS:** *m*/*z* (M + H)^+^ = 499.

#### 2-(2-Bromo-4-(3-ethyl-4-((4-fluorobenzyl)­amino)-1-methyl-1H-pyrazolo­[3,4-*d*]­pyrimidin-6-yl)­phenyl)­propan-2-ol (25)

To a stirred solution of **24** (100 mg, 0.2 mmol) in dry THF was added MeMgCl (0.5 mmol) at 0 °C under inert atmosphere. The resulting mixture was stirred at 60 °C for 4 h and then quenched with the addition of saturated NH_4_Cl solution under ice. It was then extracted with EtOAc (2 × 10 mL) and washed with brine (5 mL). The combined organic layer was then dried over anhydrous MgSO_4_ and concentrated under reduced pressure to afford the crude. The crude was then purified by chromatography (0–60% EtOAc/Hexane) to afford the pure product (28.9 mg, 29%). ^**1**^ **H NMR:** (400 MHz, DMSO) δ 8.47 (d, *J* = 1.4 Hz, 1H), 8.27 (dd, *J* = 8.3, 1.4 Hz, 1H), 7.92 (d, *J* = 8.3 Hz, 1H), 7.87 (t, *J* = 5.8 Hz, 1H), 7.48 (dd, *J* = 8.3, 5.8 Hz, 2H), 7.14 (t, *J* = 8.8 Hz, 2H), 5.33 (s, 1H), 4.80 (d, *J* = 5.7 Hz, 2H), 3.89 (s, 3H), 3.03 (q, *J* = 7.4 Hz, 2H), 1.64 (s, 6H), 1.28 (t, *J* = 7.4 Hz, 3H); ^**19**^ **F NMR:** (377 MHz, DMSO) δ −116.39 (s); **LCMS:** *m*/*z* (M + H)^+^ = 499.

#### 1-(2-Bromo-5-(3-ethyl-4-((4-fluorobenzyl)­amino)-1-methyl-1H-pyrazolo­[3,4-*d*]­pyrimidin-6-yl)­phenyl)-2,2,2-trifluoroethan-1-ol (19)

A solution of **6** (94 mg, 0.2 mmol) and CsF (61 mg, 0.4 mmol) in dry THF (2 mL) was cooled to −78 °C under inert atmosphere. Then, CF_3_-TMS (34.1 mg, 0.24 mmol) in dry THF (1 mL) was added slowly, and the resulting solution was stirred at −78 °C for 2–4 h. After completion, the reaction was quenched with the addition of ice-cold water and extracted with EtOAc. The crude was purified by chromatography (0–60% EtOAc/Hexane) to afford the title product (39.8 mg, 37%). ^**1**^ **H NMR:** (400 MHz, DMSO) δ 8.75 (d, *J* = 1.7 Hz, 1H), 8.30 (dd, *J* = 8.4, 2.2 Hz, 1H), 7.92 (t, *J* = 6.1 Hz, 1H), 7.77 (d, *J* = 8.4 Hz, 1H), 7.59–7.43 (m, 2H), 7.24 (d, *J* = 5.5 Hz, 1H), 7.17–7.00 (m, 2H), 5.50 (p, *J* = 6.7 Hz, 1H), 4.90–4.61 (m, 2H), 3.89 (s, 3H), 3.03 (q, *J* = 7.5 Hz, 2H), 1.27 (t, *J* = 7.5 Hz, 3H); ^**19**^ **F NMR:** (377 MHz, DMSO) δ −76.33 (s), −116.33 (s); **LCMS:** *m*/*z* (M + H)^+^ = 539.

#### (*E*)-N-Benzyl-6-(4-chloro-3-(2-ethoxyvinyl)­phenyl)-3-ethyl-1-methyl-1H-pyrazolo­[3,4-*d*]­pyrimidin-4-amine (33)

A solution of **32** (137 mg, 0.3 mmol), (*E*)-2-(2-ethoxyvinyl)-4,4,5,5-tetramethyl-1,3,2-dioxaborolane (65.3 mg, 0.33 mmol) and K_2_CO_3_ (83.4 mg, 0.6 mmol) in 1,4-dioxane (4 mL) was degassed for 10 min and then Pd­(dppf)­Cl_2_ (11 mg, 0.015 mmol) was added. The resulting mixture was then stirred at 80 °C for 4 h. After completion, the reaction was cooled to room temperature and then passed through a short Celite pad and washed with EtOAc. Volatiles were removed under reduced pressure to afford the crude, and then the crude was purified by normal-phase chromatography (0–60% EtOAc/Hexane) to afford the title compound (69.9 mg, 52%). ^**1**^ **H NMR:** (400 MHz, DMSO) δ 8.41 (d, *J* = 2.1 Hz, 1H), 8.12 (dd, *J* = 8.4, 2.1 Hz, 1H), 7.86 (t, *J* = 6.0 Hz, 1H), 7.47 (dd, *J* = 12.9, 4.5 Hz, 3H), 7.31 (dd, *J* = 10.3, 4.7 Hz, 2H), 7.22 (d, *J* = 7.3 Hz, 1H), 7.17 (d, *J* = 12.8 Hz, 1H), 6.05 (d, *J* = 12.8 Hz, 1H), 4.85 (d, *J* = 5.9 Hz, 2H), 3.99 (q, *J* = 7.0 Hz, 2H), 3.93–3.87 (m, 3H), 3.04 (q, *J* = 7.5 Hz, 2H), 1.29 (q, *J* = 7.3 Hz, 6H); **LCMS:** *m*/*z* (M + H)^+^ = 449.

#### 2-(5-(4-(Benzylamino)-3-ethyl-1-methyl-1H-pyrazolo­[3,4-*d*]­pyrimidin-6-yl)-2-chlorophenyl)­acetaldehyde (34)

To a stirred solution of **33** (69.9 mg, 0.15 mmol) in MeOH (1 mL) were added a few drops of conc. HCl and stirred for 1 h. Volatiles were then removed, and the crude was washed with NaHCO_3_ solution. It was then extracted with EtOAc and concentrated under reduced pressure and purified by normal-phase chromatography (0–80% EtOAc/Hexane) to afford the title compound (46 mg, 73%). ^**1**^ **H NMR:** (400 MHz, DMSO) δ 9.75 (s, 1H), 8.40–8.21 (m, 2H), 7.86 (t, *J* = 5.9 Hz, 1H), 7.56 (d, *J* = 8.4 Hz, 1H), 7.45 (d, *J* = 7.5 Hz, 2H), 7.31 (t, *J* = 7.5 Hz, 2H), 7.20 (t, *J* = 7.3 Hz, 1H), 4.85 (d, *J* = 5.9 Hz, 2H), 3.89 (s, 3H), 3.07–3.01 (m, 2H), 1.28 (t, *J* = 7.5 Hz, 3H); **LCMS:** *m*/*z* (M + H)^+^ = 421.

#### 3-(2-Chloro-5-(3-ethyl-4-((4-fluorobenzyl)­amino)-1-methyl-1H-pyrazolo­[3,4-*d*]­pyrimidin-6-yl)­phenyl)­propanoic acid (37)

A stirred solution of **36** (95 mg, 0.2 mmol), ethyl 3-(4,4,5,5-tetramethyl-1,3,2-dioxaborolan-2-yl)­propanoate (55 mg, 0.24 mmol) and K_2_CO_3_ (55.6 mg, 0.4 mmol) in 1,4-dioxane (4 mL) was degassed for 10 min, and then Pd­(dppf)­Cl_2_ (7.3 mg, 0.001 mmol) was added. The resulting mixture was then stirred at 80 °C for 4 h. After completion, the reaction was cooled to room temperature and then passed through a short Celite pad and washed with EtOAc. Volatiles were removed under reduced pressure to afford the crude and then the crude was purified by normal-phase chromatography (0–10% MeOH/DCM) to afford the title compound (44.9 mg, 48%). ^**1**^ **H NMR:** (400 MHz, DMSO) δ 12.28 (s, 1H), 8.32 (d, *J* = 2.0 Hz, 1H), 8.21 (dd, *J* = 8.4, 2.1 Hz, 1H), 7.85 (t, *J* = 5.9 Hz, 1H), 7.51 (dd, *J* = 11.3, 4.7 Hz, 3H), 7.18–7.08 (m, 2H), 4.81 (d, *J* = 5.8 Hz, 2H), 3.89 (s, 3H), 3.09–2.88 (m, 4H), 2.59 (t, *J* = 7.7 Hz, 2H), 1.28 (t, *J* = 7.5 Hz, 3H); ^**19**^ **F NMR:** (377 MHz, DMSO) δ −116.38 (s) **LCMS:** *m*/*z* (M + H)^+^ = 469.

### Synthesis of Final Compounds

#### General Procedure B for the Synthesis of 11–14, 16, 20, 26, 29, and 30

A solution of the corresponding alcohol (1.0 equiv), B_2_(OH)_4_ (2.0 equiv) and KOAc (3.0 equiv) in EtOH (2–3 mL) was degassed for 10 min. Then, XphosPdG2 (5.0 mol %) was added, and the resulting mixture was heated to 80 °C for 1 h. After completion, the reaction mixture was passed through a short Celite pad and washed with EtOAc. Volatiles were removed under reduced pressure to afford the crude. The crude was then purified by reversed-phase chromatography (5–100% CH_3_CN/H_2_O/0.1% formic acid gradient) to afford the pure product.

#### 5-(3-Ethyl-4-((4-fluorobenzyl)­amino)-1-methyl-1H-pyrazolo­[3,4-*d*]­pyrimidin-6-yl)-3-methylbenzo­[*c*]­[1,2]­oxaborol-1­(3H)-ol (11)

Using general procedure B, **7** (19.3 mg, 0.04 mmol) was converted to **11** (13.1 mg, 74%) as a white solid; ^**1**^ **H NMR:** (400 MHz, DMSO) δ 9.16 (s, 1H), 8.36 (d, *J* = 7.8 Hz, 1H), 8.28 (s, 1H), 7.84 (t, *J* = 5.8 Hz, 1H), 7.76 (d, *J* = 7.6 Hz, 1H), 7.50 (dd, *J* = 8.4, 5.7 Hz, 2H), 7.13 (t, *J* = 8.9 Hz, 2H), 5.30 (q, *J* = 6.6 Hz, 1H), 4.93–4.74 (m, 2H), 3.91 (s, 3H), 3.04 (q, *J* = 7.4 Hz, 2H), 1.44 (d, *J* = 6.6 Hz, 3H), 1.28 (t, *J* = 7.5 Hz, 3H); ^**19**^ **F NMR:** (376 MHz, DMSO) δ −116.46 (s) ^**13**^ **C NMR:** (101 MHz, DMSO) δ 161.5 (d, *J* = 242.8 Hz), 160.6, 158.9, 156.7, 155.4, 145.7, 141.0, 137.0 (d, *J* = 2.9 Hz), 130.7, 129.6 (d, *J* = 8.1 Hz), 127.3, 121.0, 115.5, 115.3, 97.7, 77.0, 43.7, 33.6, 23.0, 21.9, 14.0; **HRMS: (M + H)** ^**+**^ Calcd for C_23_H_24_BFN_5_O_2_ 432.2001 found 432.1997.

#### 3-Ethyl-5-(3-ethyl-4-((4-fluorobenzyl)­amino)-1-methyl-1H-pyrazolo­[3,4-*d*]­pyrimidin-6-yl)­benzo­[*c*]­[1,2]­oxaborol-1­(3H)-ol (12)

Using general procedure B, **8** (19.9 mg, 0.04 mmol) was converted to **12** (13.3 mg, 75%) as a white solid; ^**1**^ **H NMR:** (400 MHz, DMSO) δ 9.17 (s, 1H), 8.36 (d, *J* = 7.7 Hz, 1H), 8.27 (s, 1H), 7.83 (t, *J* = 5.9 Hz, 1H), 7.75 (d, *J* = 7.7 Hz, 1H), 7.49 (dd, *J* = 8.6, 5.6 Hz, 2H), 7.18–7.06 (m, 2H), 5.17 (dd, *J* = 7.2, 3.8 Hz, 1H), 4.83 (qd, *J* = 15.1, 5.9 Hz, 2H), 3.90 (d, *J* = 5.0 Hz, 3H), 3.04 (q, *J* = 7.4 Hz, 2H), 2.00 (ddd, *J* = 14.0, 7.3, 3.9 Hz, 1H), 1.57 (dt, *J* = 14.2, 7.3 Hz, 1H), 1.28 (t, *J* = 7.5 Hz, 3H), 0.88 (q, *J* = 7.2 Hz, 3H); ^**19**^ **F NMR: (**376 MHz, DMSO) δ −116.50 (s); **HRMS: (M + H)** ^**+**^ Calcd for C_24_H_26_BFN_5_O_2_ 446.2157 found 446.2151.

#### 5-(3-Ethyl-4-((4-fluorobenzyl)­amino)-1-methyl-1H-pyrazolo­[3,4-*d*]­pyrimidin-6-yl)-3-isopropylbenzo­[*c*]­[1,2]­oxaborol-1­(3H)-ol (13)

Using general procedure B, **9** (20.5 mg, 0.04 mmol) was converted to **13** (12.8 mg, 70%) as a white solid; ^**1**^ **H NMR: (**400 MHz, DMSO) δ 9.10 (s, 1H), 8.29 (d, *J* = 7.8 Hz, 1H), 8.21 (s, 1H), 7.77 (t, *J* = 5.8 Hz, 1H), 7.69 (d, *J* = 7.7 Hz, 1H), 7.42 (dd, *J* = 8.5, 5.7 Hz, 2H), 7.05 (t, *J* = 8.9 Hz, 2H), 5.06 (d, *J* = 2.9 Hz, 1H), 4.77 (ddd, *J* = 31.7, 15.3, 6.0 Hz, 2H), 3.84 (s, 3H), 2.97 (q, *J* = 7.4 Hz, 2H), 2.14–2.04 (m, 1H), 1.22 (t, *J* = 7.5 Hz, 3H), 1.02 (d, *J* = 6.8 Hz, 3H), 0.49 (d, *J* = 6.8 Hz, 3H); ^**19**^ **F NMR:** (376 MHz, DMSO) δ −116.53 (s); **HRMS: (M + H)** ^**+**^ Calcd for C_25_H_28_BFN_5_O_2_ 460.2314 found 460.2305.

#### 5-(3-Ethyl-4-((4-fluorobenzyl)­amino)-1-methyl-1H-pyrazolo­[3,4-*d*]­pyrimidin-6-yl)-3-phenylbenzo­[*c*]­[1,2]­oxaborol-1­(3H)-ol (14)

Using general procedure B, **10** (21.9 mg, 0.04 mmol) was converted to **14** (15.2 mg, 77%) as a white solid; ^**1**^ **H NMR: (**400 MHz, DMSO) δ 9.47 (s, 1H), 8.40 (d, *J* = 7.7 Hz, 1H), 8.13 (s, 1H), 7.83 (dd, *J* = 14.4, 6.9 Hz, 2H), 7.48–7.35 (m, 4H), 7.33 (d, *J* = 7.4 Hz, 3H), 7.03 (t, *J* = 8.8 Hz, 2H), 6.28 (s, 1H), 4.75 (ddd, *J* = 60.5, 15.0, 6.0 Hz, 2H), 3.86 (s, 3H), 3.01 (q, *J* = 7.4 Hz, 2H), 1.27 (t, *J* = 7.4 Hz, 3H); ^**19**^ **F NMR**: (376 MHz, DMSO) δ −116.34 (s); **HRMS: (M + H)** ^**+**^ Calcd for C_28_H_26_BFN_5_O_2_ 494.2157 found 494.2149.

#### 5-(3-Ethyl-4-((4-fluorobenzyl)­amino)-1-methyl-1H-pyrazolo­[3,4-*d*]­pyrimidin-6-yl)-3,3-dimethylbenzo­[*c*]­[1,2]­oxaborol-1­(3H)-ol (16)

Using general procedure B, **15** (19.9 mg, 0.04 mmol) was converted to **16** (13 mg, 73%) as an off-white solid; ^**1**^ **H NMR:** (400 MHz, DMSO) δ 9.08 (s, 1H), 8.33 (d, *J* = 7.7 Hz, 1H), 8.19 (s, 1H), 7.86 (t, *J* = 5.8 Hz, 1H), 7.72 (d, *J* = 7.7 Hz, 1H), 7.50 (dd, *J* = 8.4, 5.6 Hz, 2H), 7.13 (t, *J* = 8.9 Hz, 2H), 4.82 (d, *J* = 5.7 Hz, 2H), 3.90 (d, *J* = 6.6 Hz, 3H), 3.05 (q, *J* = 7.4 Hz, 2H), 1.49 (s, 6H), 1.29 (dd, *J* = 9.8, 5.1 Hz, 3H); **HRMS: (M + H)** ^**+**^ Calcd for C_24_H_26_BFN_5_O_2_ 446.2157 found 446.2156.

#### 5-(3-Ethyl-4-((4-fluorobenzyl)­amino)-1-methyl-1H-pyrazolo­[3,4-*d*]­pyrimidin-6-yl)-3-(trifluoromethyl)­benzo­[*c*]­[1,2]­oxaborol-1­(3H)-ol (20)

Using general procedure B, **19** (21.5 mg, 0.04 mmol) was converted to **20** (13.9 mg, 72%) as a light gray solid; ^**1**^ **H NMR:** (400 MHz, DMSO) δ 9.99 (s, 1H), 8.53 (d, *J* = 7.8 Hz, 1H), 8.49 (s, 1H), 7.89 (d, *J* = 7.3 Hz, 2H), 7.49 (dd, *J* = 8.3, 5.9 Hz, 2H), 7.12 (t, *J* = 8.9 Hz, 2H), 5.98 (d, *J* = 6.2 Hz, 1H), 4.83 (ddd, *J* = 32.9, 15.2, 5.9 Hz, 2H), 3.90 (d, *J* = 3.7 Hz, 3H), 3.04 (q, *J* = 7.4 Hz, 2H), 1.28 (t, *J* = 7.5 Hz, 3H); ^**19**^ **F NMR:** (376 MHz, DMSO) δ −76.58 (s), −116.32––116.45 (m); **HRMS: (M + H)** ^**+**^ Calcd for C_23_H_21_BF_4_N_5_O_2_ 486.1718 found 486.1715.

#### 6-(3-Ethyl-4-((4-fluorobenzyl)­amino)-1-methyl-1H-pyrazolo­[3,4-*d*]­pyrimidin-6-yl)-3,3-dimethylbenzo­[*c*]­[1,2]­oxaborol-1­(3*H*)-ol (26)

Using general procedure B, **25** (19.9 mg, 0.04 mmol) was converted to **26** (13.2 mg, 74%) as an off-white solid; ^**1**^ **H NMR:** (400 MHz, DMSO) δ 9.13 (s, 1H), 8.72 (s, 1H), 8.49–8.40 (m, 1H), 7.77 (t, *J* = 5.9 Hz, 1H), 7.58–7.40 (m, 3H), 7.13 (t, *J* = 8.8 Hz, 2H), 4.88 (d, *J* = 5.8 Hz, 2H), 3.90 (s, 3H), 3.02 (q, *J* = 7.4 Hz, 2H), 1.48 (s, 6H), 1.27 (t, *J* = 7.4 Hz, 3H); ^**19**^ **F NMR:** (376 MHz, DMSO) δ −116.34 (s); **HRMS: (M + H)** ^**+**^ C_24_H_26_BFN_5_O_2_ 446.2157 found 446.2157.

#### 6-(3-Ethyl-4-((4-fluorobenzyl)­amino)-1-methyl-1H-pyrazolo­[3,4-*d*]­pyrimidin-6-yl)-3-methylbenzo­[*c*]­[1,2]­oxaborol-1­(3H)-ol (29)

Using general procedure B, **28** (19.4 mg, 0.04 mmol) was converted to **29** (12.6 mg, 74%) as a white solid; ^**1**^ **H NMR:** (400 MHz, DMSO) δ 9.14 (s, 1H), 8.71 (s, 1H), 8.42 (dd, *J* = 8.0, 1.1 Hz, 1H), 7.70 (t, *J* = 6.0 Hz, 1H), 7.48–7.36 (m, 3H), 7.06 (t, *J* = 8.9 Hz, 2H), 5.20 (q, *J* = 6.5 Hz, 1H), 4.82 (d, *J* = 5.9 Hz, 2H), 3.84 (s, 3H), 2.96 (q, *J* = 7.4 Hz, 2H), 1.36 (d, *J* = 6.6 Hz, 3H), 1.20 (t, *J* = 7.5 Hz, 3H); ^19^F NMR (376 MHz, DMSO) δ −116.33 (s); **HRMS: (M + H)** ^**+**^ Calcd for C_23_H_24_BFN_5_O_2_ 432.2001found 432.1998.

#### 6-(3-Ethyl-4-((4-fluorobenzyl)­amino)-1-methyl-1H-pyrazolo­[3,4-*d*]­pyrimidin-6-yl)­benzo­[*c*]­[1,2]­oxaborol-1­(3H)-ol (30)

To a stirred solution of **27** (93.6 mg, 0.2 mmol) in MeOH was added NaBH_4_ (9.1 mg, 0.24 mmol) at 0 °C under inert atmosphere, and the resulting mixture was stirred for 20 min. After completion, a saturated solution of NH_4_Cl was added and extracted with EtOAc (10 mL). The organic layer was then concentrated under reduced pressure to afford the crude (83.7 mg, crude yield 89%). The crude was taken to the next step without purification; **LCMS:** *m*/*z* (M + H)^+^ = 471. Next, using general procedure B, the crude (80 mg, 0.17 mmol) was converted to **30** (55.3 mg, 78%) as a white solid; ^**1**^ **H NMR:** (400 MHz, DMSO) δ 9.23 (s, 1H), 8.76 (s, 1H), 8.44 (d, *J* = 7.7 Hz, 1H), 7.70 (t, *J* = 5.9 Hz, 1H), 7.51–7.33 (m, 3H), 7.06 (t, *J* = 8.8 Hz, 2H), 4.98 (s, 2H), 4.82 (d, *J* = 5.7 Hz, 2H), 3.84 (s, 3H), 2.96 (q, *J* = 7.4 Hz, 2H), 1.20 (t, *J* = 7.4 Hz, 3H) ppm; ^**19**^ **F NMR:** (376 MHz, DMSO) δ −116.32; ^**13**^ **C NMR:** (101 MHz, DMSO) δ 160.94, 160.94, 160.36, 156.78, 156.78, 156.42, 156.42, 155.52, 155.52, 145.64, 145.64, 137.53, 136.95, 136.92, 131.09, 130.82, 129.80, 129.80, 129.72, 129.72, 121.62, 115.51, 115.51, 115.30, 115.30, 97.57, 70.40, 43.36, 33.53, 21.94, 14.00.; **LCMS:** *m*/*z* (M + H)^+^ = 418; **HRMS: (M + H)** ^**+**^ Calcd for C_22_H_22_BFN_5_O_2_ 418.1844 found 419.1837.

#### 5-(3-Ethyl-4-{[(*p*-fluorophenyl)­methyl]­amino}-1-methyl-1*H*-1,2,5,7-tetraazainden-6-yl)-1-hydroxy-2,1-benzoxaborol-3­(1*H*)-one (18)

To a stirred solution of **5** (49.8 mg, 0.1 mmol) in 1,4-dioxane were added KOAc (19.6 mg, 0.2 mmol) and B_2_pin_2_ (50.8 mg, 0.2 mmol) and degassed for 10 min. Then, Pd­(dppf)­Cl_2_ (7.31 mg, 0.01 mmol) was added, and the reaction mixture was heated to 80 °C for 6 h. After completion, the reaction mixture was passed through a short Celite pad and washed with EtOAc. Volatiles were removed under reduced pressure to afford the crude. The crude was then dissolved in MeOH and cooled to 0 °C, then 1 M NaOH (0.2 mmol) solution was added. After completion, 1 M HCl was added, and the volatiles were removed under reduced pressure, and the crude was purified by reversed-phase chromatography (5–100% Acetonitrile/Water/0.1% formic acid). Pure fractions were then lyophilized to afford the pure product as a white solid (6.46 mg, 15% over two steps), ^**1**^ **H NMR** (400 MHz, DMSO) δ 8.47 (s, 1H), 8.36 (d, *J* = 7.6 Hz, 1H), 7.72 (s, 1H), 7.52 (s, 2H), 7.39 (d, *J* = 7.7 Hz, 1H), 7.14 (t, *J* = 8.3 Hz, 2H), 4.87 (s, 2H), 3.90 (s, 3H), 3.02 (d, *J* = 6.5 Hz, 2H), 1.27 (t, *J* = 6.3 Hz, 3H) ppm; **LCMS:** *m*/*z* (M + H)^+^ = 432; **HRMS: (M + H)** ^**+**^ Calcd for C_22_H_20_BFN_5_O_3_ 432.1637 found 432.1649.

#### 6-(3-Ethyl-4-((4-fluorobenzyl)­amino)-1-methyl-1H-pyrazolo­[3,4-*d*]­pyrimidin-6-yl)-1H-benzo­[*d*]­[1,2,6]­oxazaborinin-1-ol (22)

A stirred solution of **6** (46.8 mg, 0.1 mmol), B_2_pin_2_ (31 mg, 0.12 mmol), and KOAc (19.6 mg, 0.2 mmol) in 1,4-dioxane was degassed for 10 min, and Pd­(dppf)­Cl_2_ (7.31 mg, 0.01 mmol) was added. The resulting solution was then heated to 80 °C for 6 h. After completion, the reaction mixture was passed through a short Celite pad and washed with EtOAc. Volatiles were removed under reduced pressure to afford the crude. The crude was then dissolved in DCM (1–2 mL) and was added aq. NH_2_OH (0.12 mmol). The resulting mixture was stirred vigorously for 1 h. After that, 5% TFA in DCM was added (0.4 mL), and the resulting mixture was stirred for another 1 h. Volatiles were then removed under reduced pressure to afford the crude. The crude was then purified by reversed-phase chromatography (5–100% acetonitrile/water/0.1% formic acid). Pure fractions were then lyophilized to afford the pure product as a white solid (9.5 mg, 22% over 3 steps). ^**1**^ **H NMR**: (400 MHz, CDCl_3_) δ 8.85 (d, *J* = 7.9 Hz, 1H), 8.69 (s, 1H), 8.53 (s, 1H), 8.16 (d, *J* = 7.8 Hz, 1H), 7.46 (d, *J* = 7.3 Hz, 2H), 7.40 (t, *J* = 7.3 Hz, 2H), 7.33 (t, *J* = 6.7 Hz, 1H), 5.52 (s, 1H), 5.04 (d, *J* = 5.6 Hz, 2H), 4.70 (s, 1H), 4.06 (s, 3H), 2.95 (q, *J* = 7.6 Hz, 2H), 1.40 (t, *J* = 7.5 Hz, 3H); ^**19**^ **F NMR:** δ −116.32; **LCMS:** *m*/*z* (M + H)^+^ = 431; **HRMS: (M + H)** ^**+**^ Calcd for C_22_H_21_BFN_6_O_2_ 431.1798 found 431.1790.

#### 6-(4-(Benzylamino)-3-ethyl-1-methyl-1H-pyrazolo­[3,4-*d*]­pyrimidin-6-yl)-3,4-dihydro-1*H*-benzo­[*c*]­[1,2]­oxaborinin-1-ol (35)

To a stirred solution of **34** (21 mg, 0.05 mmol) in MeOH was added NaBH_4_ (2.2 mg, 0.06 mmol) at 0 °C under inert atmosphere and stirred for 20 min. Volatiles were then removed under reduced pressure to afford the crude. The crude was then dried and redissolved in EtOH (2 mL). KOAc (9.8 mg, 0.1 mmol) and B_2_(OH)_4_ (13.4 mg, 0.15 mmol) were added to the reaction mixture and degassed for 10 min. Then, XphosPdG2 (1.96 mg, 0.0025 mmol) was added and heated to 80 °C for 1 h. After completion, the reaction mixture was passed through a short Celite pad and washed with EtOAc. Volatiles were removed under reduced pressure to afford the crude. The crude was then purified by reversed-phase chromatography (5–100% CH_3_CN/H_2_O/0.1% formic acid gradient) to afford the pure product as an off-white solid (14.7 mg, 71% over 2 steps); ^**1**^ **H NMR**: (400 MHz, DMSO) δ 8.49 (s, 1H), 8.23 (dd, *J* = 7.7, 1.4 Hz, 1H), 8.16 (s, 1H), 7.81 (t, *J* = 6.0 Hz, 1H), 7.74 (d, *J* = 7.7 Hz, 1H), 7.45 (d, *J* = 7.2 Hz, 2H), 7.32 (t, *J* = 7.6 Hz, 2H), 7.21 (t, *J* = 7.3 Hz, 1H), 4.86 (d, *J* = 5.9 Hz, 2H), 4.10 (t, *J* = 5.9 Hz, 2H), 3.90 (s, 3H), 3.04 (q, *J* = 7.5 Hz, 2H), 2.94 (t, *J* = 5.8 Hz, 2H), 1.28 (t, *J* = 7.5 Hz, 3H). **LCMS:** *m*/*z* (M + H)^+^ = 414; **HRMS: (M + H)** ^**+**^ Calcd for C_23_H_25_BFN_5_O_2_ 414.2095 found 414.2095.

#### 7-(3-Ethyl-4-((4-fluorobenzyl)­amino)-1-methyl-1H-pyrazolo­[3,4-*d*]­pyrimidin-6-yl)-2,3,4,5-tetrahydro-1H-benzo­[*b*]­borepin-1-ol (38)

To a stirred solution of **37** (46.7 mg, 0.1 mmol) in dry THF (2 mL) was added BH_3_ (1 M in THF, 0.2 mmol) at 0 °C, and the resulting mixture was warmed to room temperature and stirred for 4 h. After completion, the reaction mixture was cooled again to 0 °C and a saturated solution of NH_4_Cl was added. It was then extracted with EtOAc (10 mL) and concentrated under reduced pressure to afford the crude. The crude was then dried under vacuum and redissolved in EtOH (2 mL). KOAc (29.5 mg, 0.3 mmol) and B_2_(OH)_4_ (26.9 mg, 0.3 mmol) were added to the reaction mixture and degassed for 10 min. Then, XphosPdG2 (3.9 mg, 0.005 mmol) was added and heated to 80 °C for 1 h. After completion, the reaction mixture was passed through a short Celite pad and washed with EtOAc. Volatiles were removed under reduced pressure to afford the crude. The crude was then purified by reversed-phase chromatography (5–100% CH_3_CN/H_2_O/0.1% formic acid gradient) to afford the pure product as a white solid (9.8 mg, 22% over 2 steps); ^**1**^ **H NMR**: (400 MHz, DMSO) δ 8.20 (dd, *J* = 7.7, 1.5 Hz, 1H), 8.16–8.07 (m, 2H), 7.81 (t, *J* = 5.9 Hz, 1H), 7.60 (d, *J* = 7.7 Hz, 1H), 7.43–7.34 (m, 1H), 7.15 (ddd, *J* = 8.9, 5.6, 2.3 Hz, 3H), 4.84 (d, *J* = 5.8 Hz, 2H), 3.90 (s, 3H), 3.78 (t, *J* = 5.9 Hz, 2H), 3.04 (q, *J* = 7.5 Hz, 2H), 2.86 (t, *J* = 6.8 Hz, 2H), 1.99 (p, *J* = 6.5 Hz, 2H), 1.32–1.25 (m, 3H); ^**19**^ **F NMR:** (376 MHz, DMSO) δ −116.48; **LCMS:** *m*/*z* (M + H)^+^ = 446; **HRMS: (M + H)** ^**+**^ Calcd for C_24_H_26_BFN_5_O_2_ 446.2157 found 446.2157.

## Supplementary Material







## Notes

Induced fit docking model of CpPDE1 with **2** (PDB)
Molecular formula strings (CSV)
^1^H, ^13^C, and ^19^F NMR spectra, HRMS and LC-MS spectra, and chiral resolution of compound *rac-* **11**; SFC spectra of *ent1*-**11** and *ent2*-**11**; and ^1^H NMR spectra of all final compounds (PDF)

## Abbreviations Used

CC_50_: 50% cytotoxic concentration
Cp: *Cryptosporidium parvum*
EC_50_: 50% efficacious concentration
NTZ: nitazoxanide
