# Supplementary material for: Structure–Activity Relationship Study of Anti-Cryptosporidium Benzoxaboroles Yields Enhanced Potency and Curative Efficacy
Source: J Med Chem. 2026 Jul 27;69(15):19355–70. doi: 10.1021/acs.jmedchem.6c01707 (PMC13492363; doi:10.1021/acs.jmedchem.6c01707)
Supplement: Supplementary file 1 [file jm6c01707_si_003.pdf]

Supporting Information

Structure-Activity Relationship Study of Anti-*Cryptosporidium* Benzoxaboroles Yields Enhanced Potency and Curative Efficacy

Soumitra Guin,[a] José E. Teixeira,[b] Peter Miller,[b] Ankita Sarkar,[a] Christopher D. Huston,[b]* and Marvin J. Meyers[a,c]*

[a] Department of Chemistry, Saint Louis University, Saint Louis, MO 63103
[b] Department of Medicine, University of Vermont Larner College of Medicine, Burlington, VT 05401
[c] Institute for Drug and Biotherapeutic Innovation, Saint Louis University, St. Louis, MO 63103

Corresponding Authors

* E-mail: MJM: marvin.j.meyers@slu.edu. 3501 Laclede Ave, Monsanto Hall, Saint Louis, MO 63103, USA. Phone: 1-314-977-5197; CDH: christopher.huston@uvm.edu. 95 Carrigan Drive, Stafford Hall, Burlington, VT 05405, USA. Phone: 1-802-656-9115.

Table of Contents

1. **Figs. S1-5:** ^{1}H, ^{13}C, ^{19}F NMR, HRMS and LCMS of compound *rac*-**11** S2

2. **Figs. S6-8:** Chiral resolution *rac*-**11** and characterization of enantiomers S5

3. **Figs. S9-20:** ^{1}H NMR of compounds **12-14, 16, 18, 20, 22, 26, 29-30, 35, 38** S7

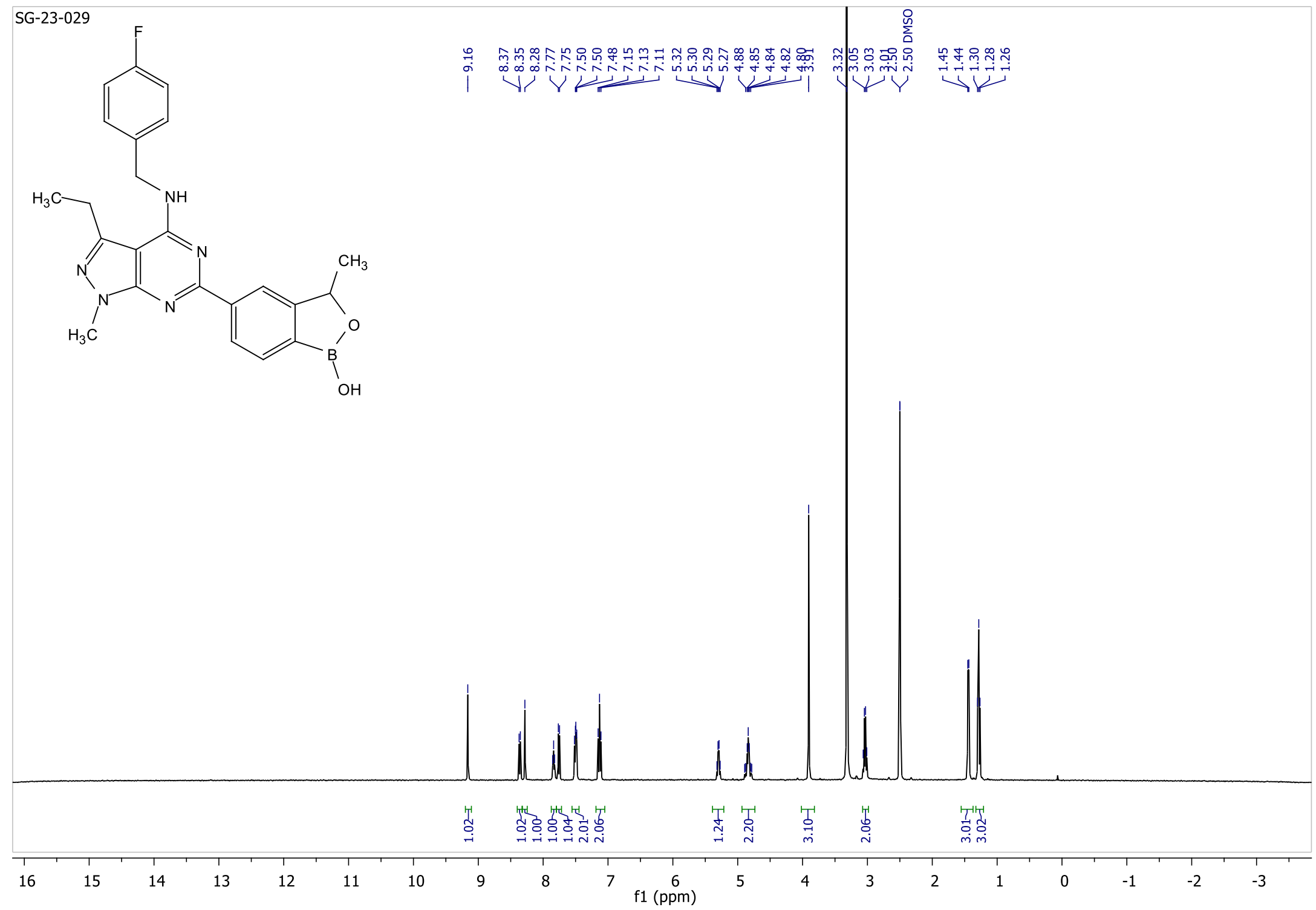

Figure S1: ^{1}H NMR of *rac*-**11** (400 MHz, DMSO).

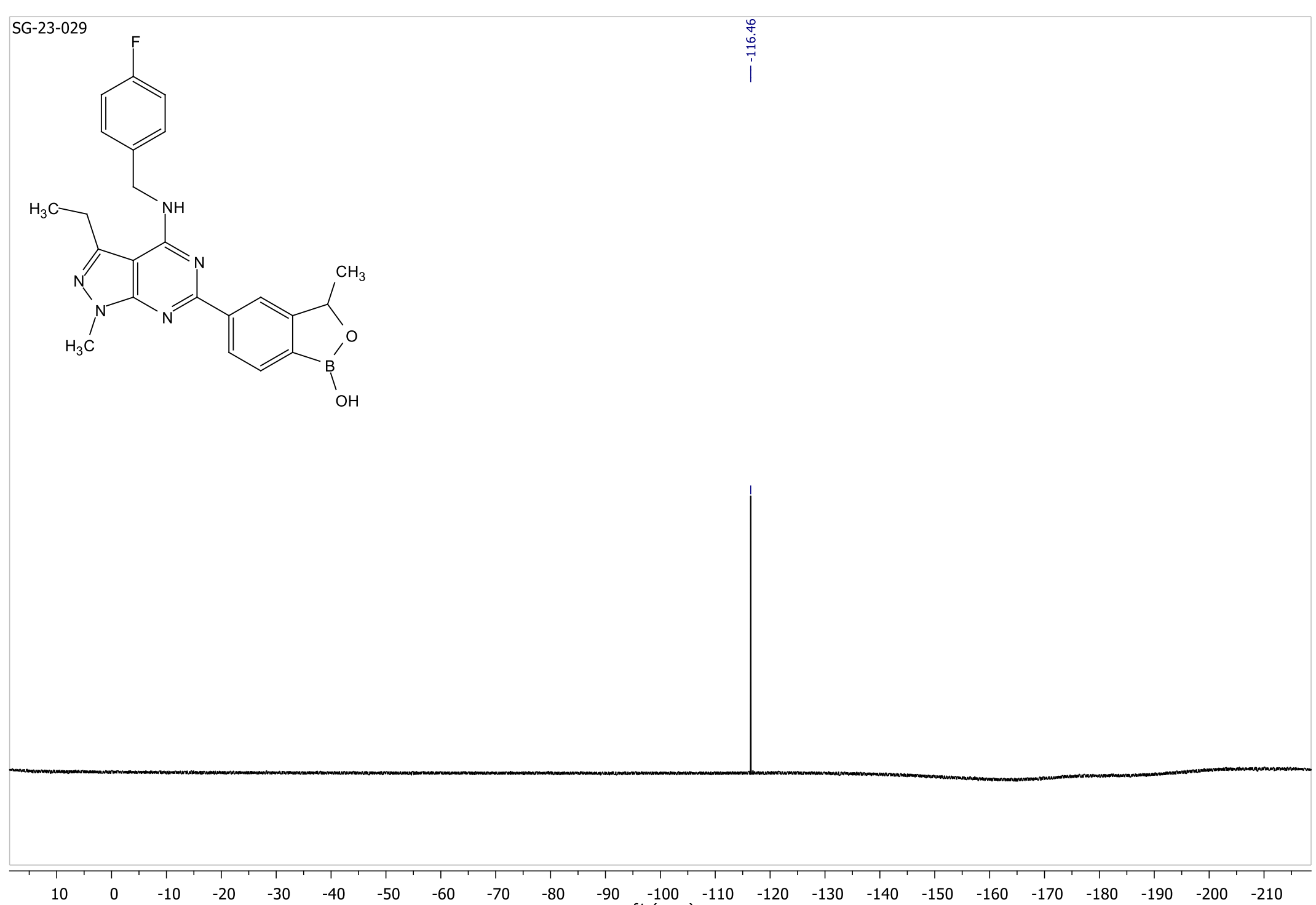

Figure S2: ^{19}F NMR of *rac*-**11** (376 MHz, DMSO).

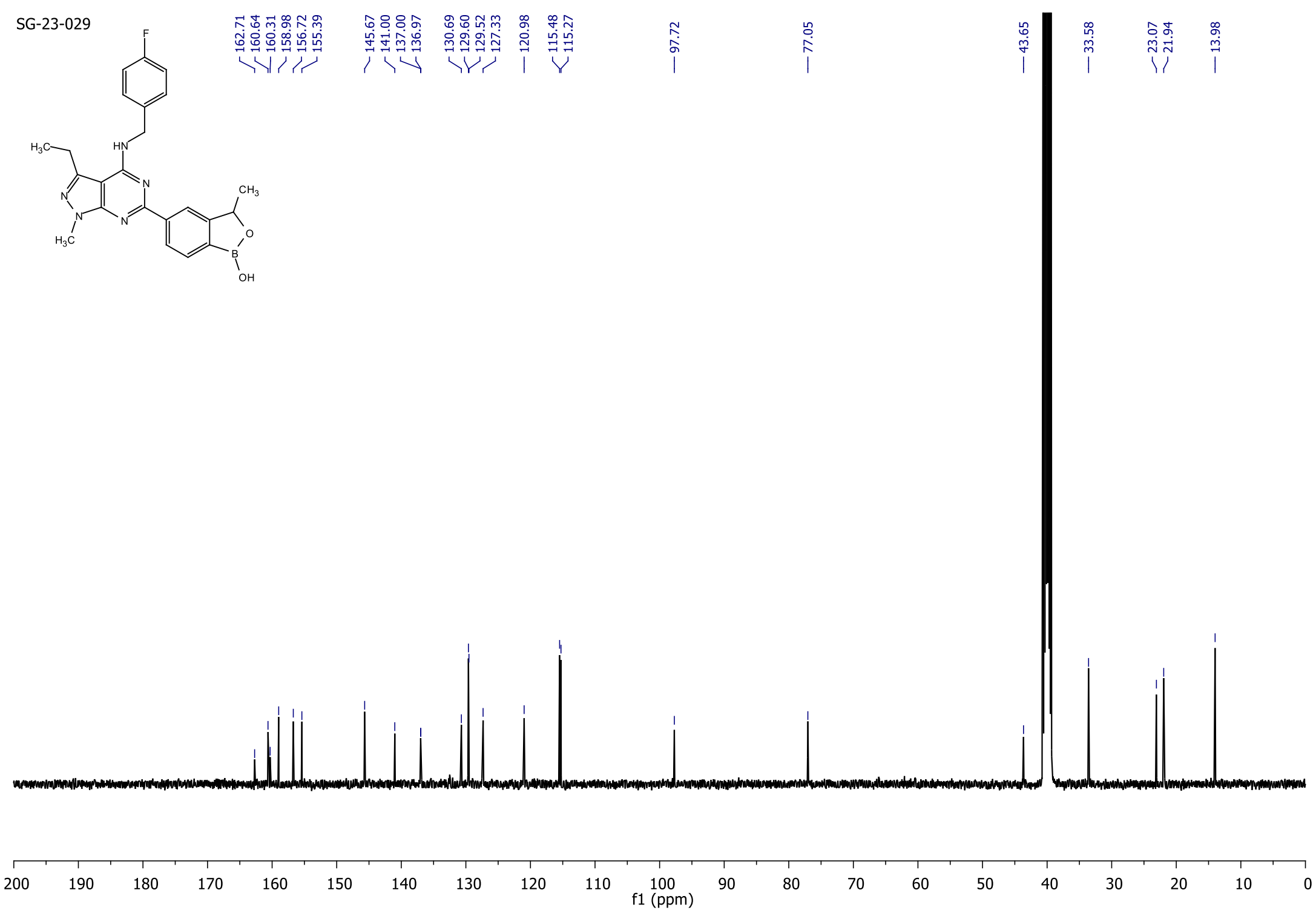

Figure S3: ^{13}C NMR of *rac*-**11** (101 MHz, DMSO).

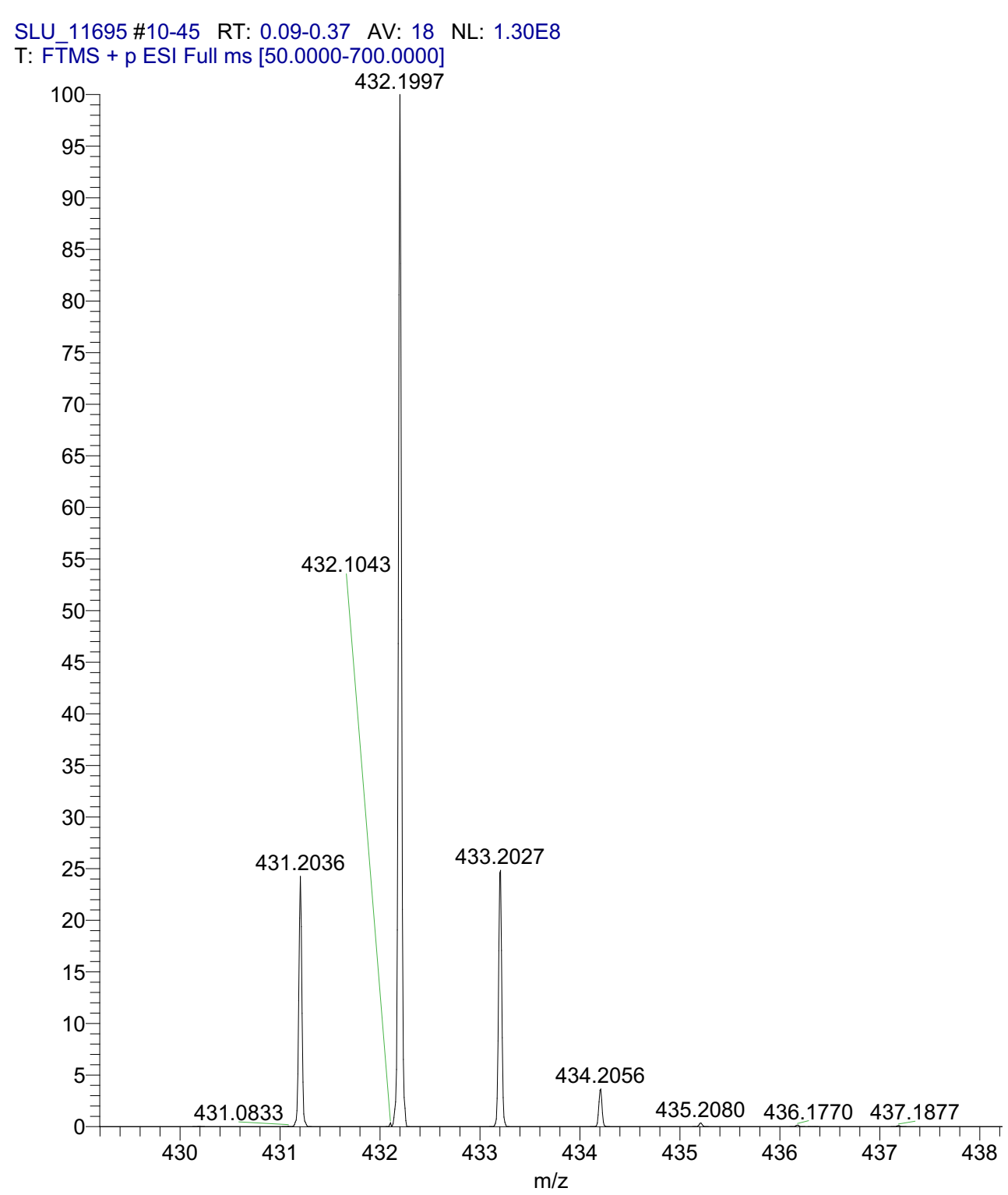

Figure S4: HRMS of *rac*-**11**.

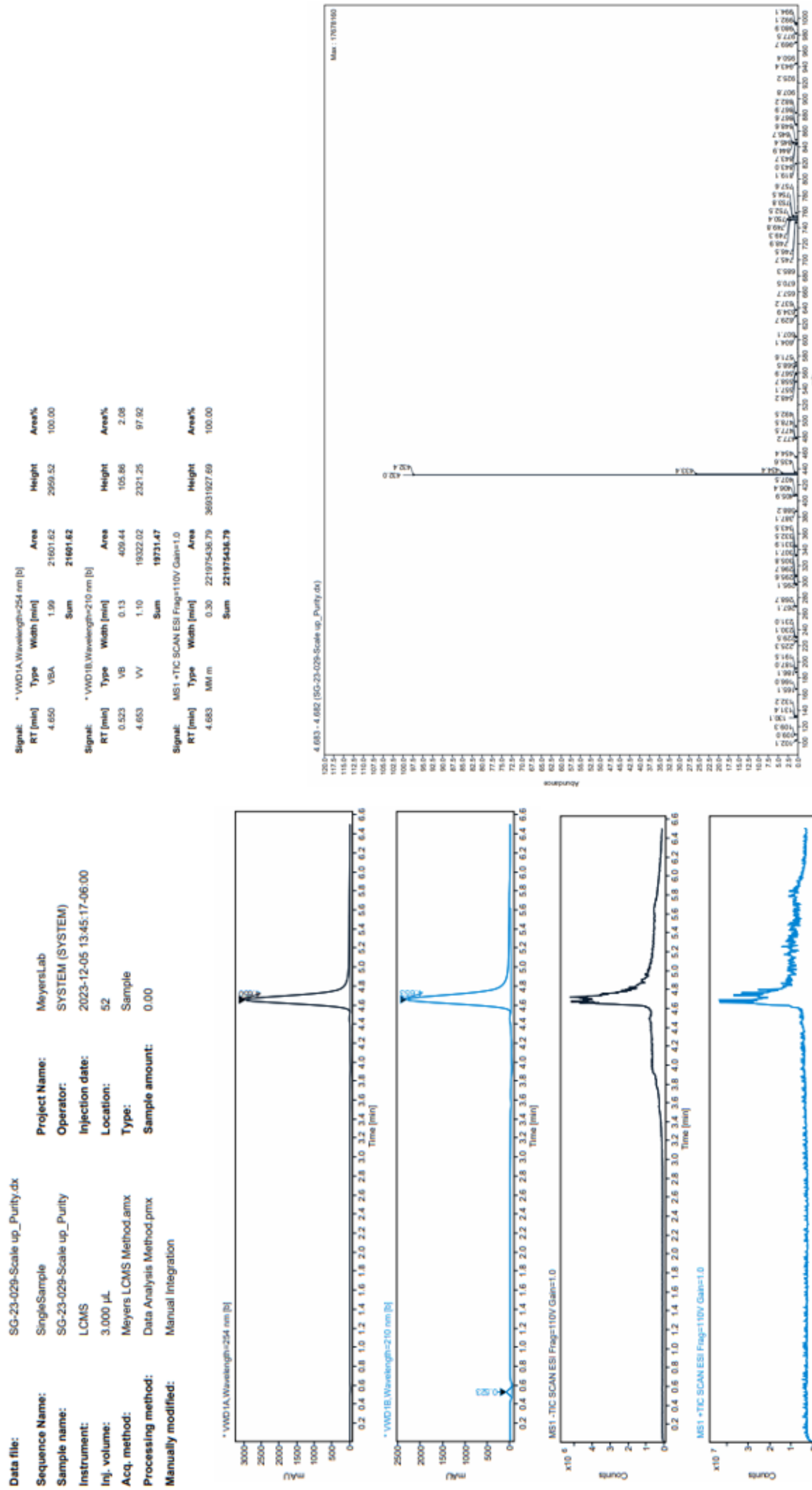

Figure S5: LCMS of *rac*-**11**.

Chiral Resolution of *rac*-11 to give *ent1*-11 and *ent2*-11. A 20 mg sample was submitted to Lotus Separations (Princeton University) for chiral separation. The enantiomers were resolved under the conditions described in the Experimental Details below, and the collected peak fractions were dried *in vacuo* at ~30°C without a co-solvent followed by a high vacuum drying at room temperature. Peak-2 (*ent2*-**11**) was reworked using the same method. The separation yielded 8 mg of peak-1 (*ent1*-**11**) and 6 mg of peak-2 (*ent2*-**11**). Both samples were obtained as clear oils which were lyophilized from acetonitrile/water to give white amorphous solids.

Experimental Details

Analytical SFC Conditions

Instrument: Berger Analytical SFC
Column: Daicel Chiralpak© AD-H, 250 (L) x 4.6 (ID) mm
Oven Temperature: 35 °C
Mobile Phase CO_2 Modifier: 15% ethanol
Flow rate: 3 mL/min
Back Pressure: 100 bar
UV Wavelength: 220(black), 254(red) and 280(blue) nm

Preparative SFC Conditions

Instrument: Berger MGII SFC
Column: Daicel Chiralpak © AD-H, 25 (L) x 2 (ID) cm
Temperature: 35 °C
Mobile Phase CO_2 Modifier: 13% ethanol
Flow rate: 60 mL/min
Back Pressure: 100 bar
UV Wavelength: 220 nm
Inj. Vol: 0.5 mL
Inj. Conc: 2 mg/mL ethanol

Analytical Traces of original material and purified products

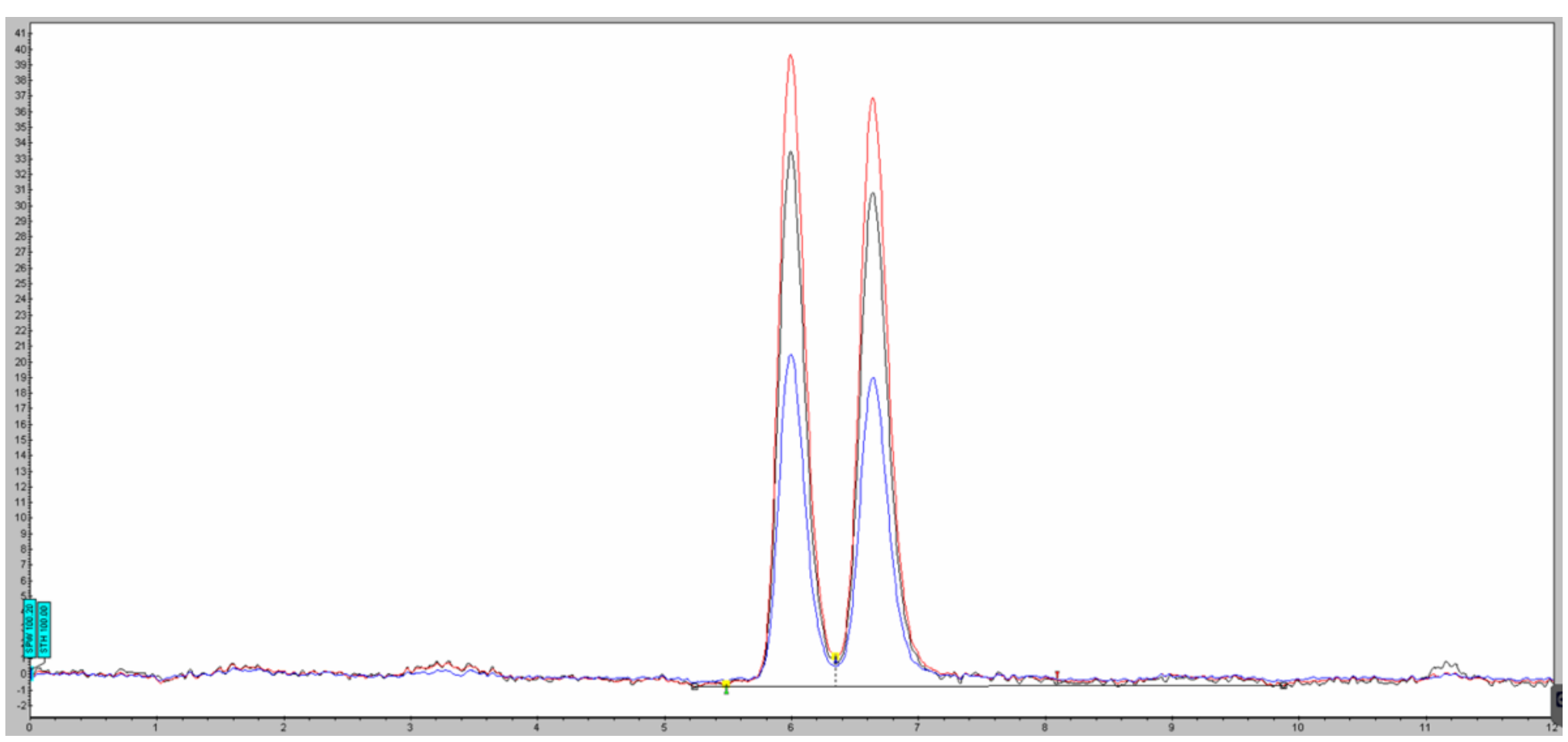

Figure S6. Analytical SFC of SLU-11695 (*rac*-11)

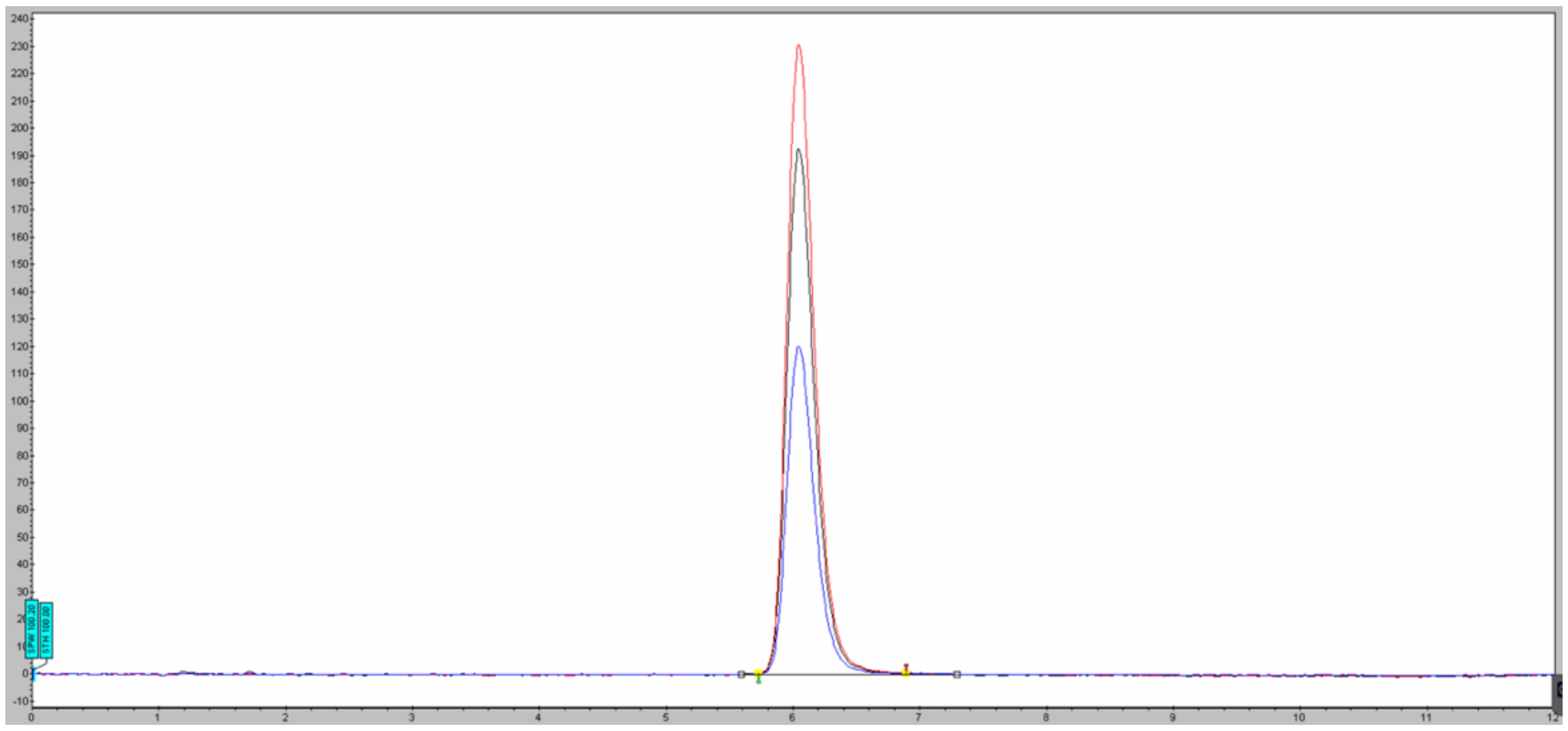

Index	Time (min)	Area (%)
Peak-1	6.05	100.00
Peak-2		
Total		100.00

Figure S7. Analytical SFC of SLU-11695 Peak-1 (*ent1*-11)

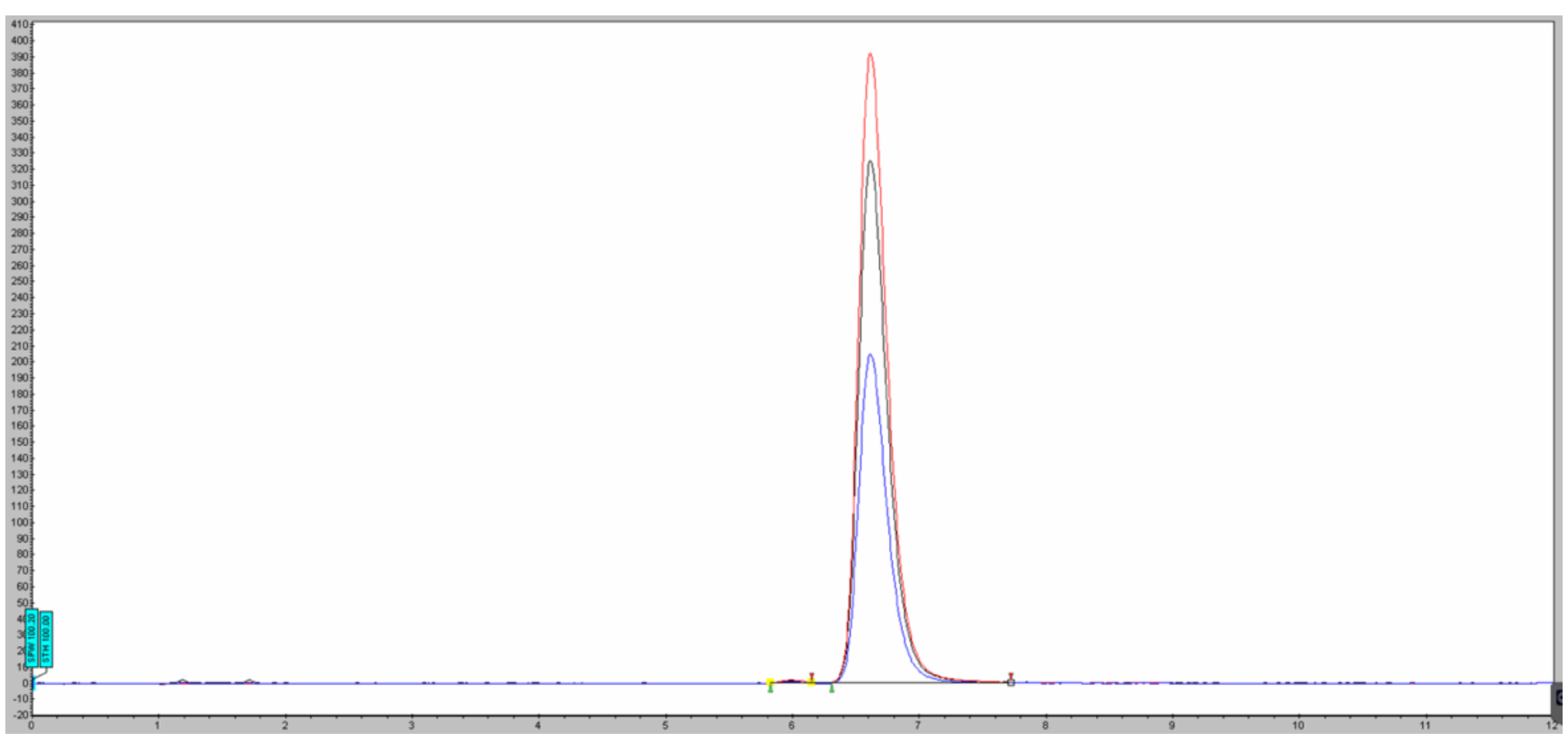

Index	Time (min)	Area (%)
Peak-1	5.99	0.288
Peak-2	6.61	99.712
Total		100.00

Figure S8. Analytical SFC of SLU-11695 Peak-2 (*ent2*-11)

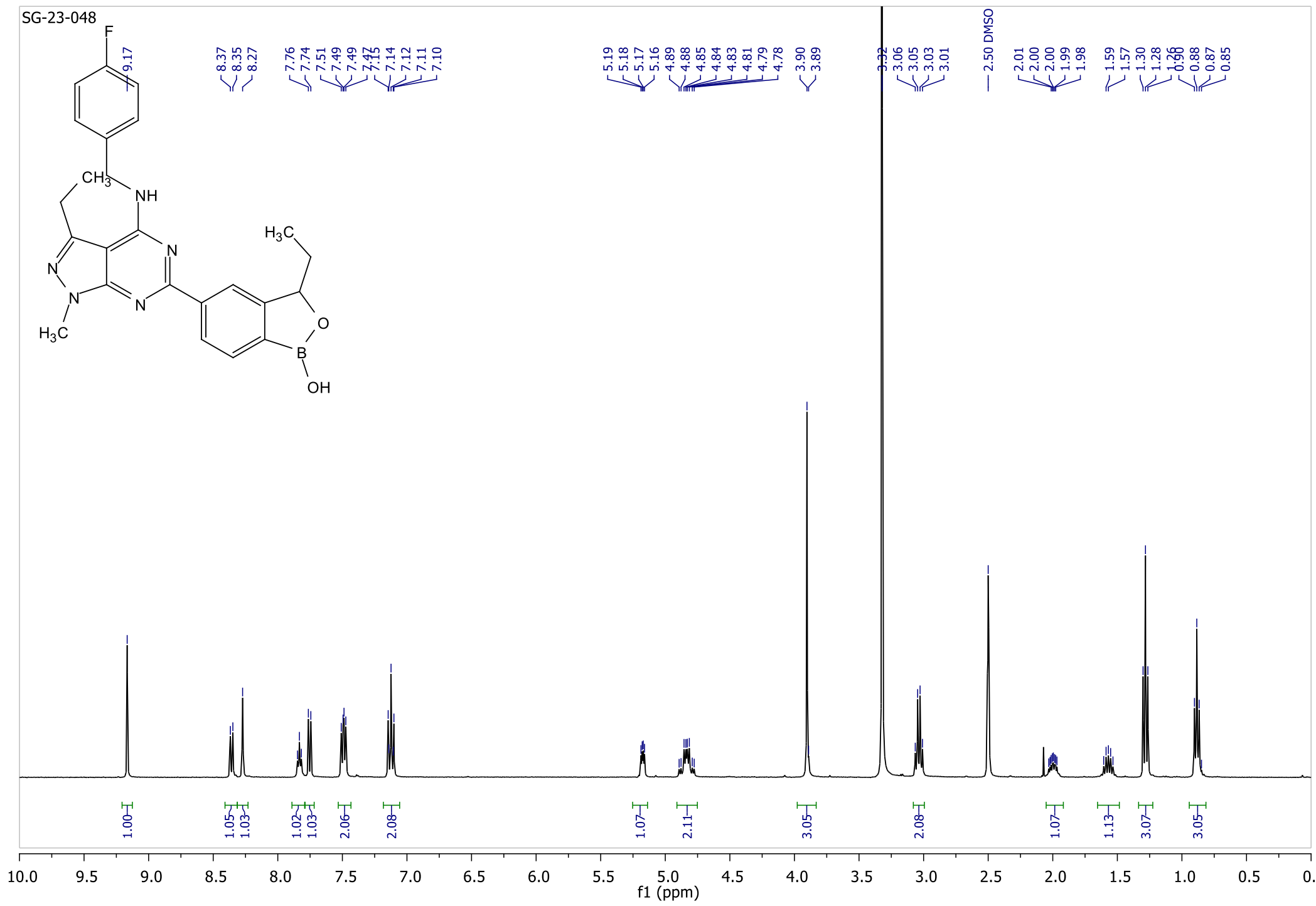

Figure S9: ^{1}H NMR of **12** (400 MHz, DMSO).

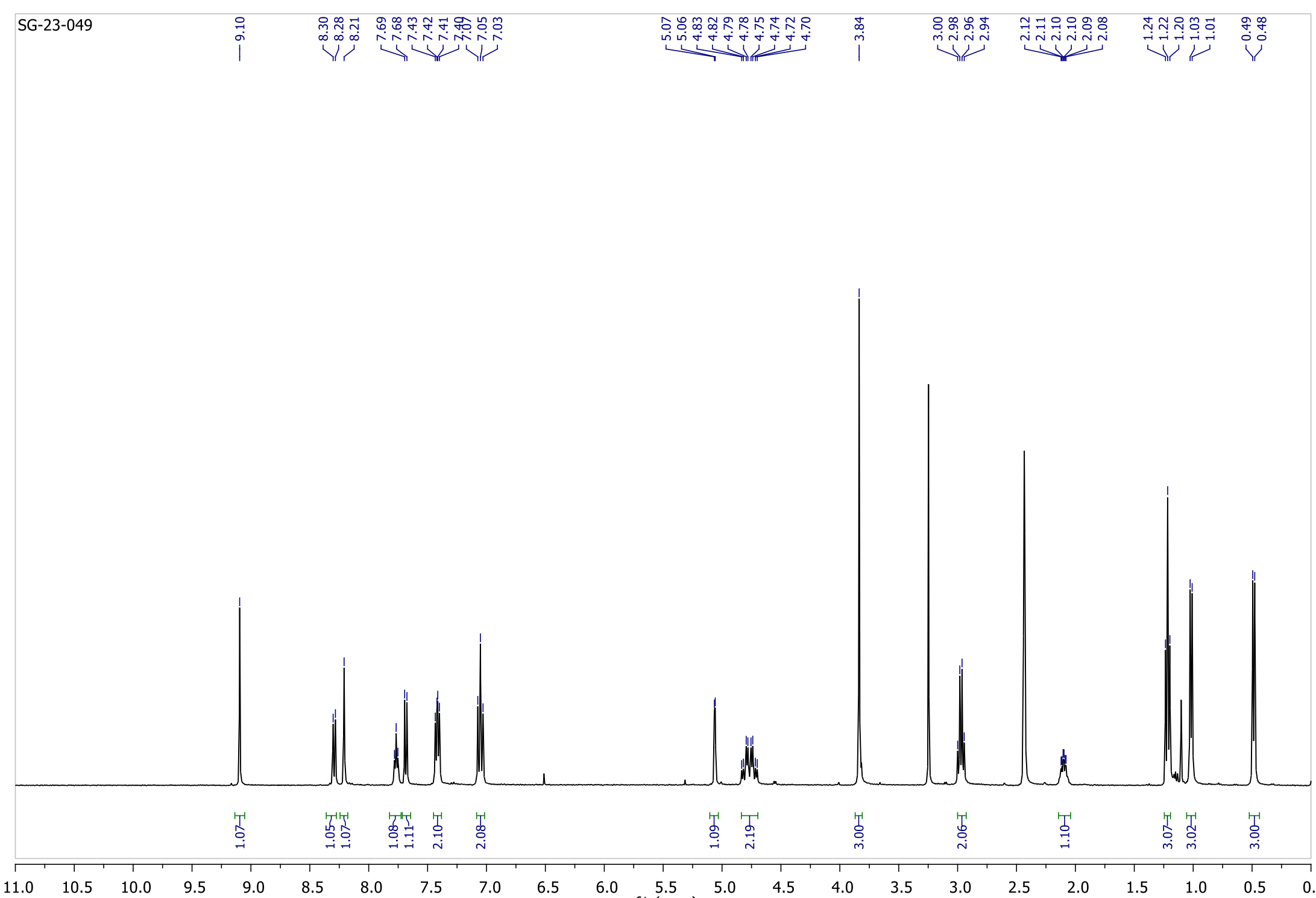

Figure S10: ^{1}H NMR of **13** (400 MHz, DMSO).

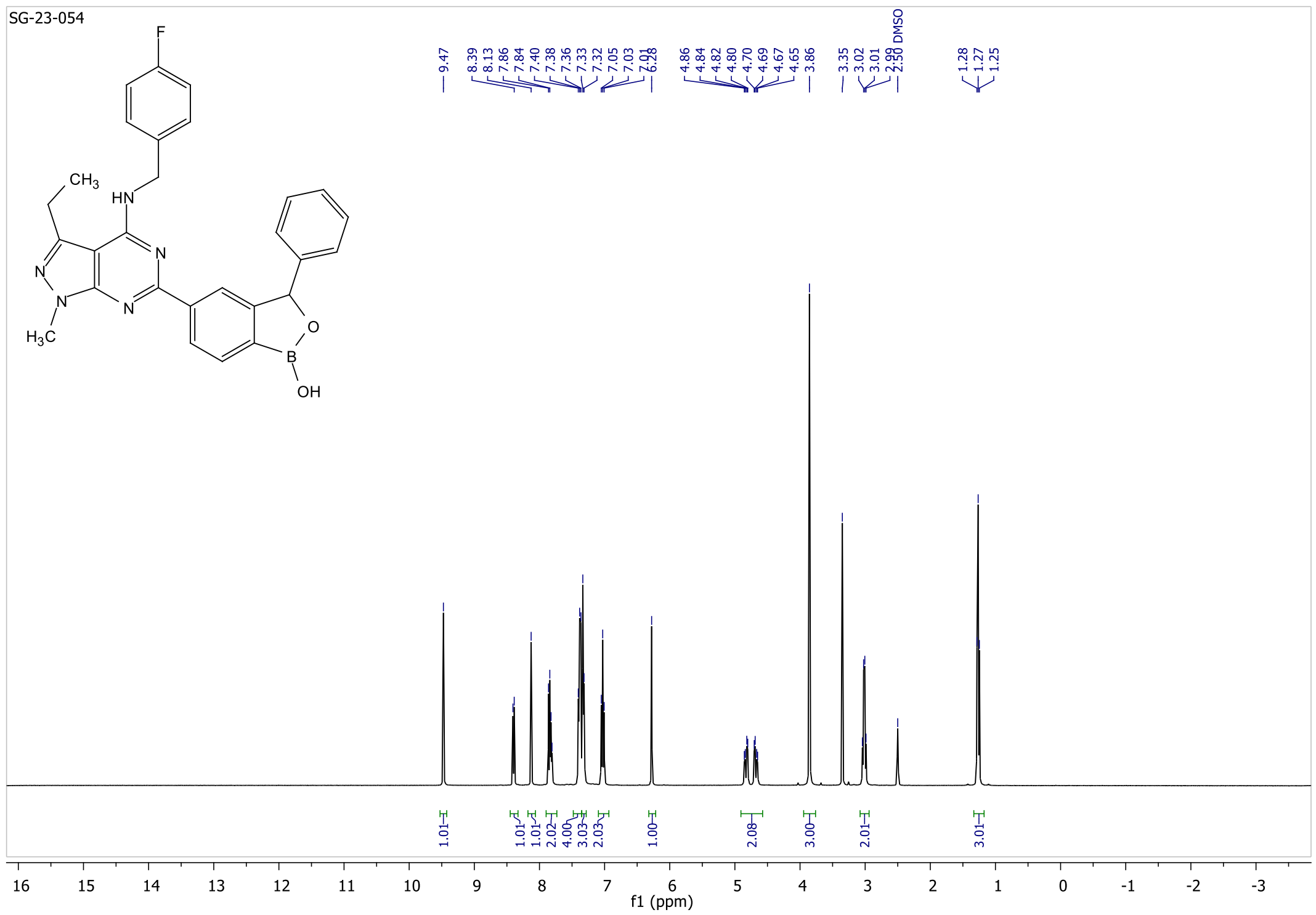

Figure S11: ^{1}H NMR of **14** (400 MHz, DMSO).

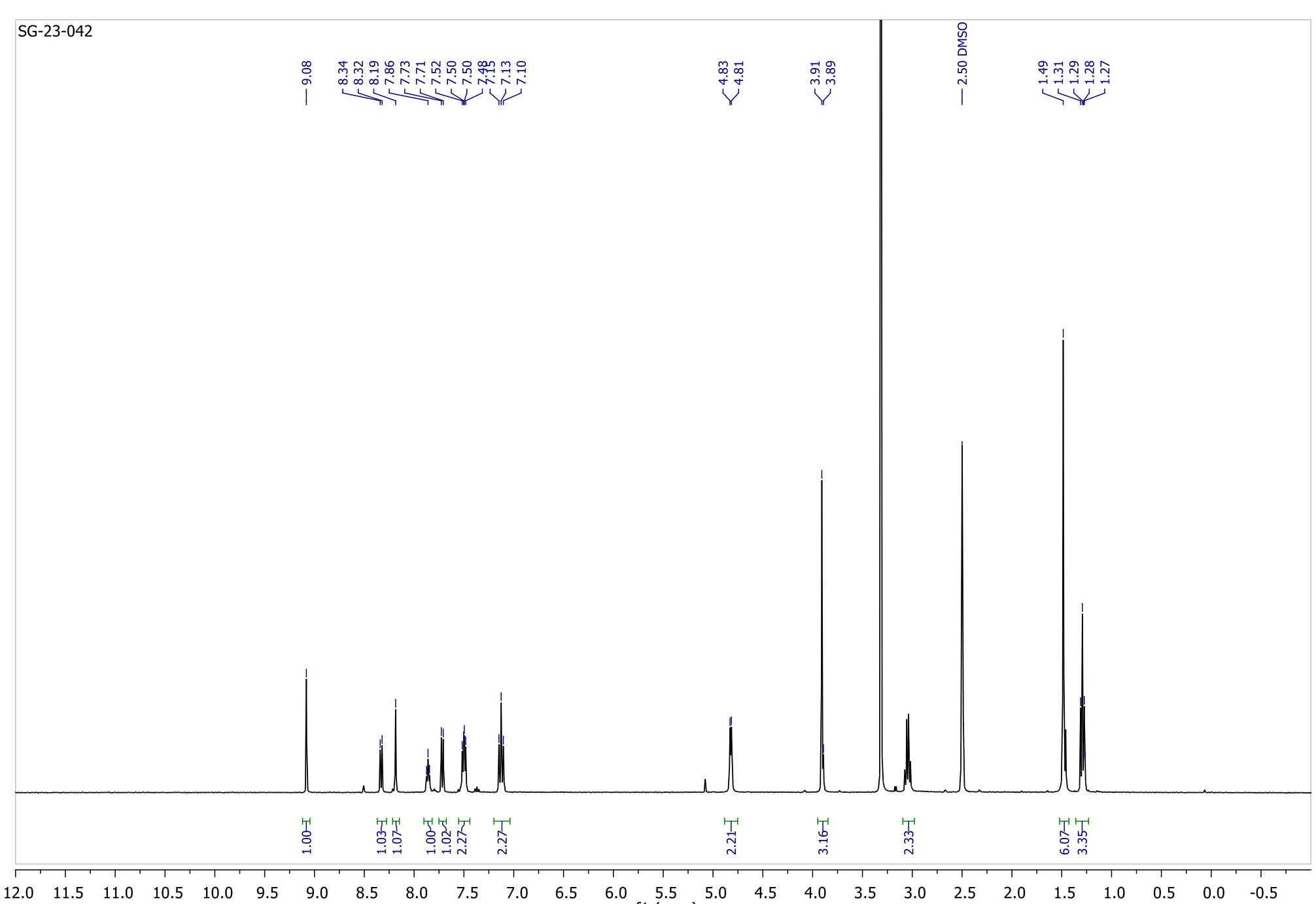

Figure S12: ^{1}H NMR of **16** (400 MHz, DMSO).

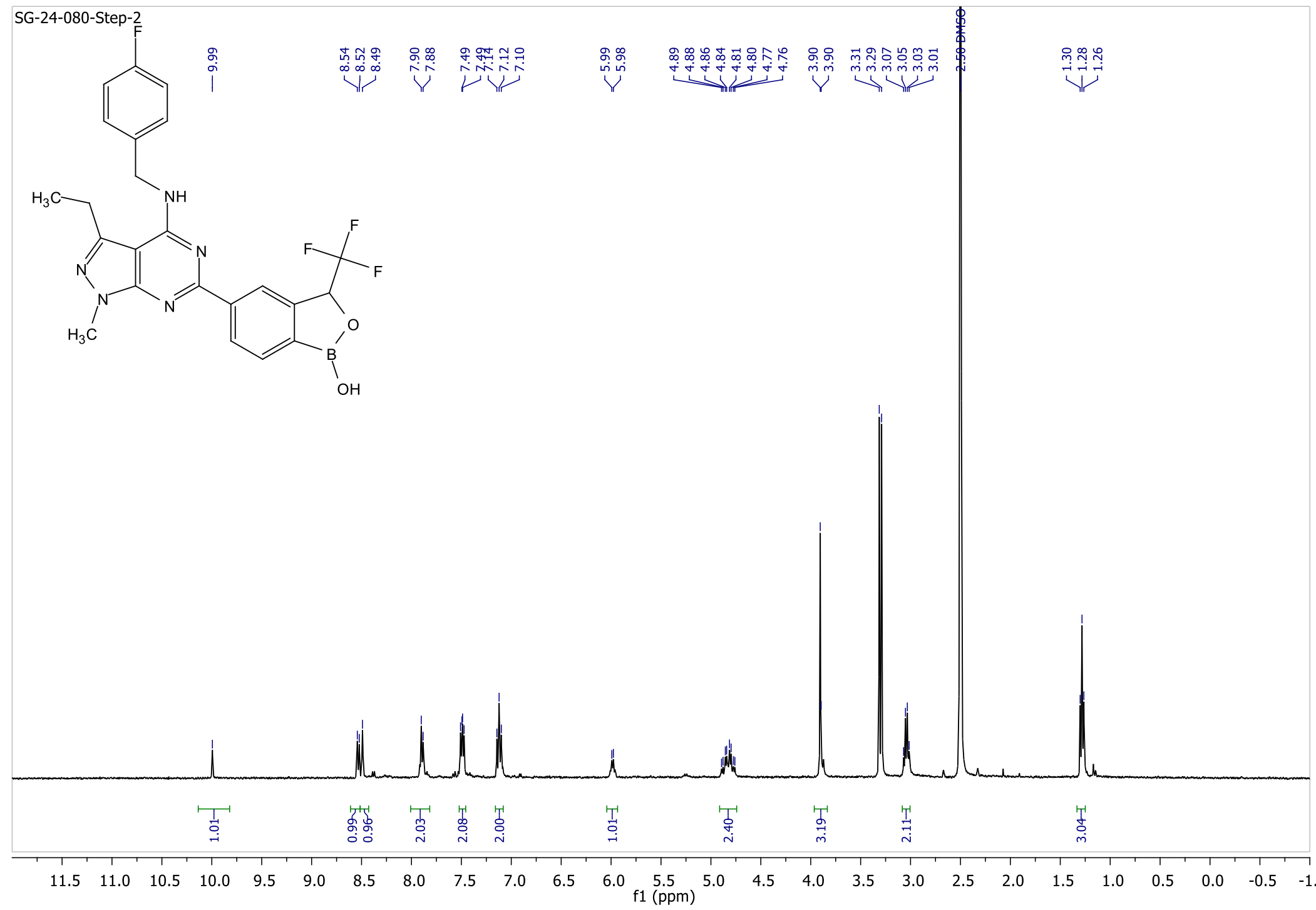

Figure S13: ^{1}H NMR of **20** (400 MHz, DMSO).

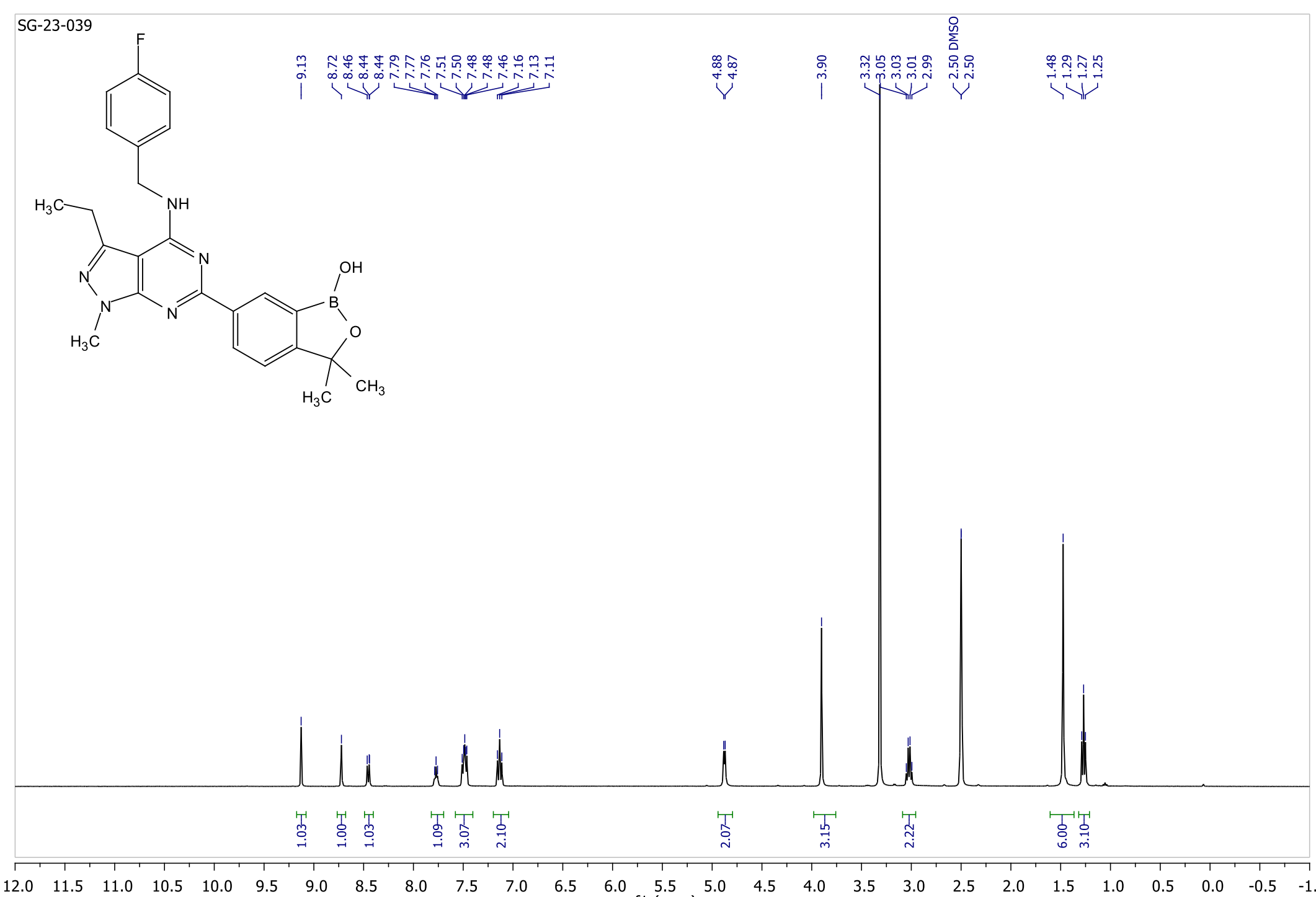

Figure S14: ^{1}H NMR of **26** (400 MHz, DMSO).

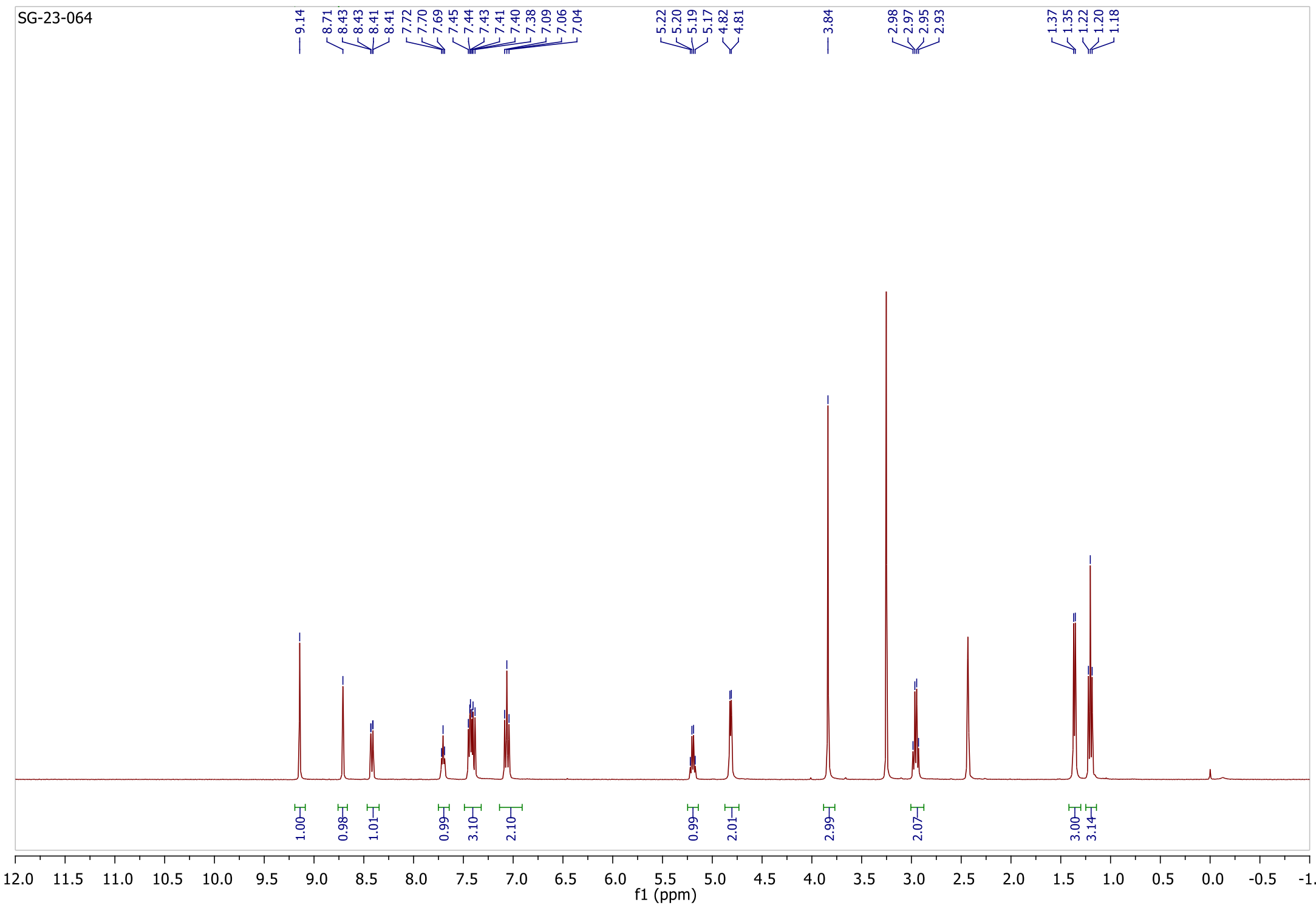

Figure S15: ^{1}H NMR of **29** (400 MHz, DMSO).

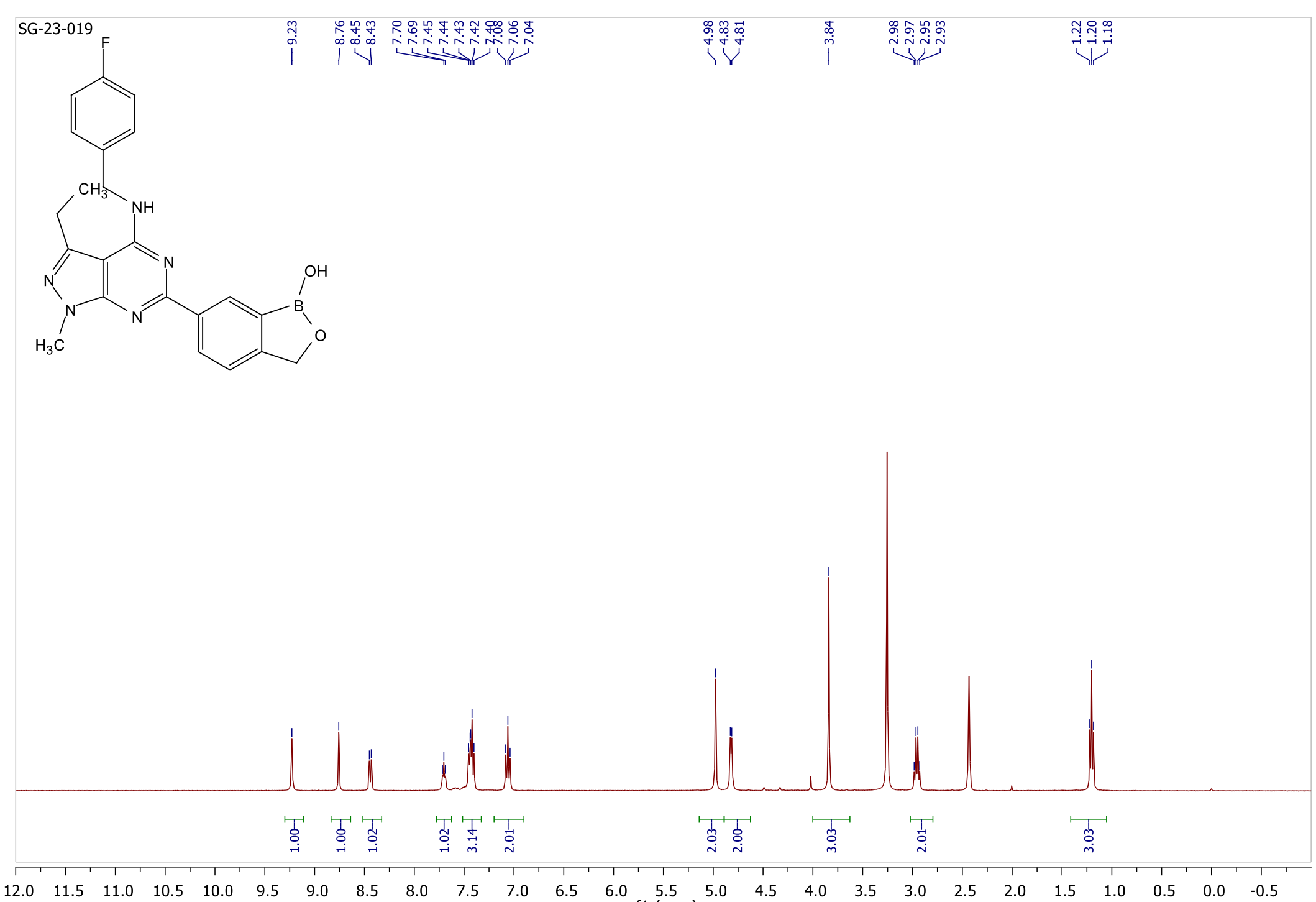

Figure S16: ^{1}H NMR of **30** (400 MHz, DMSO).

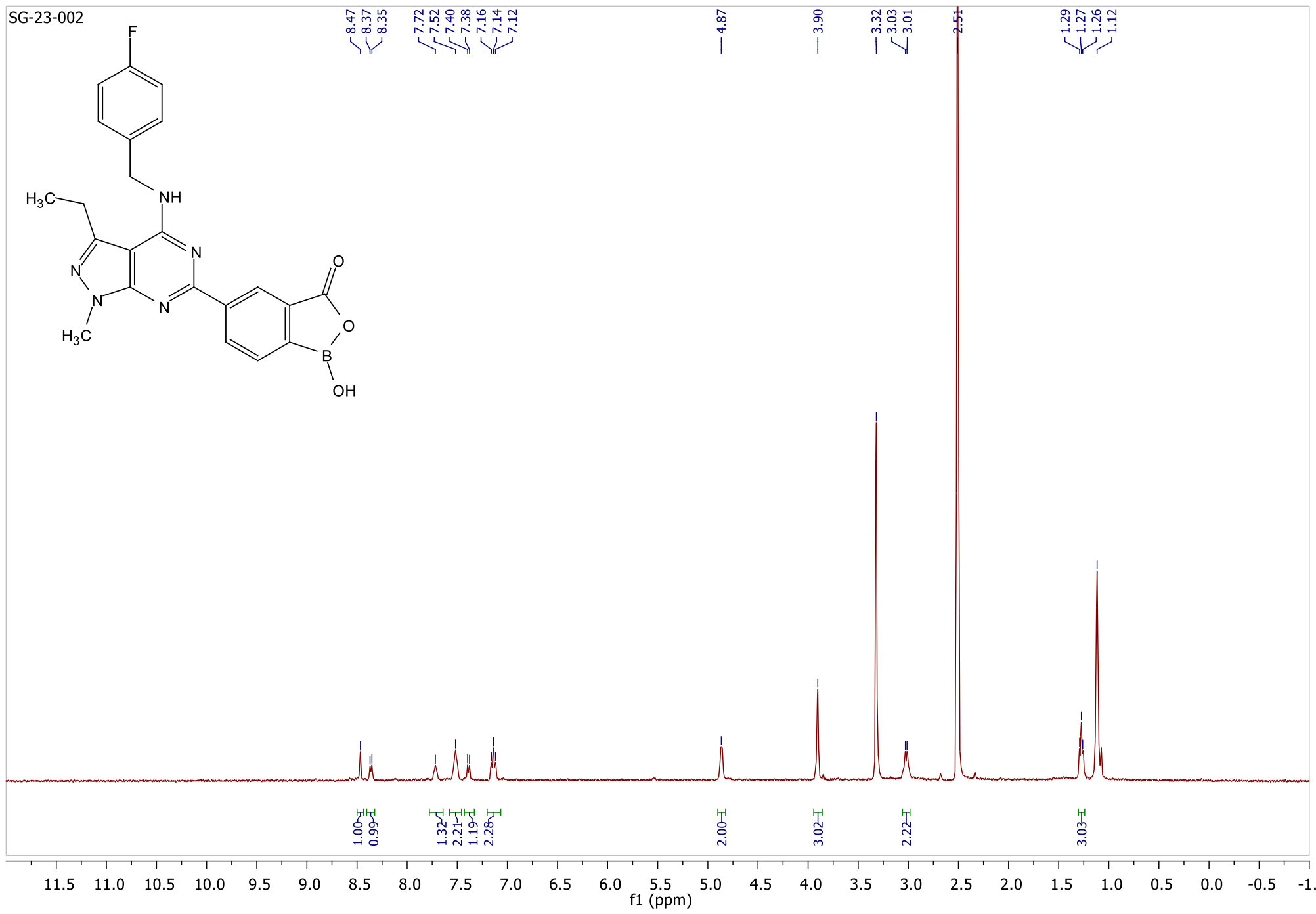

Figure S17: ^{1}H NMR of **18** (400 MHz, DMSO).

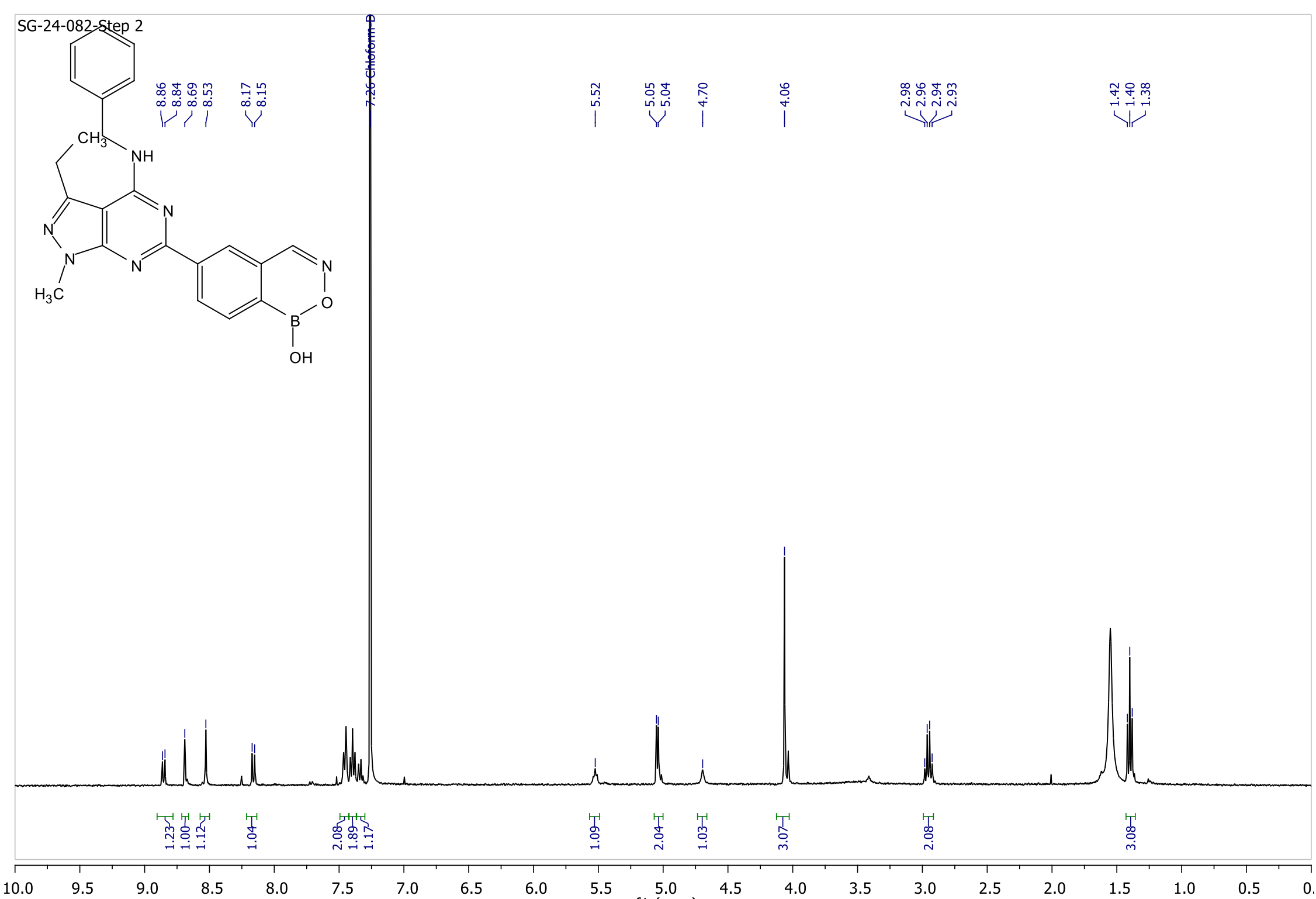

Figure S18: ^{1}H NMR of **22** (400 MHz, $CDCl_3$).

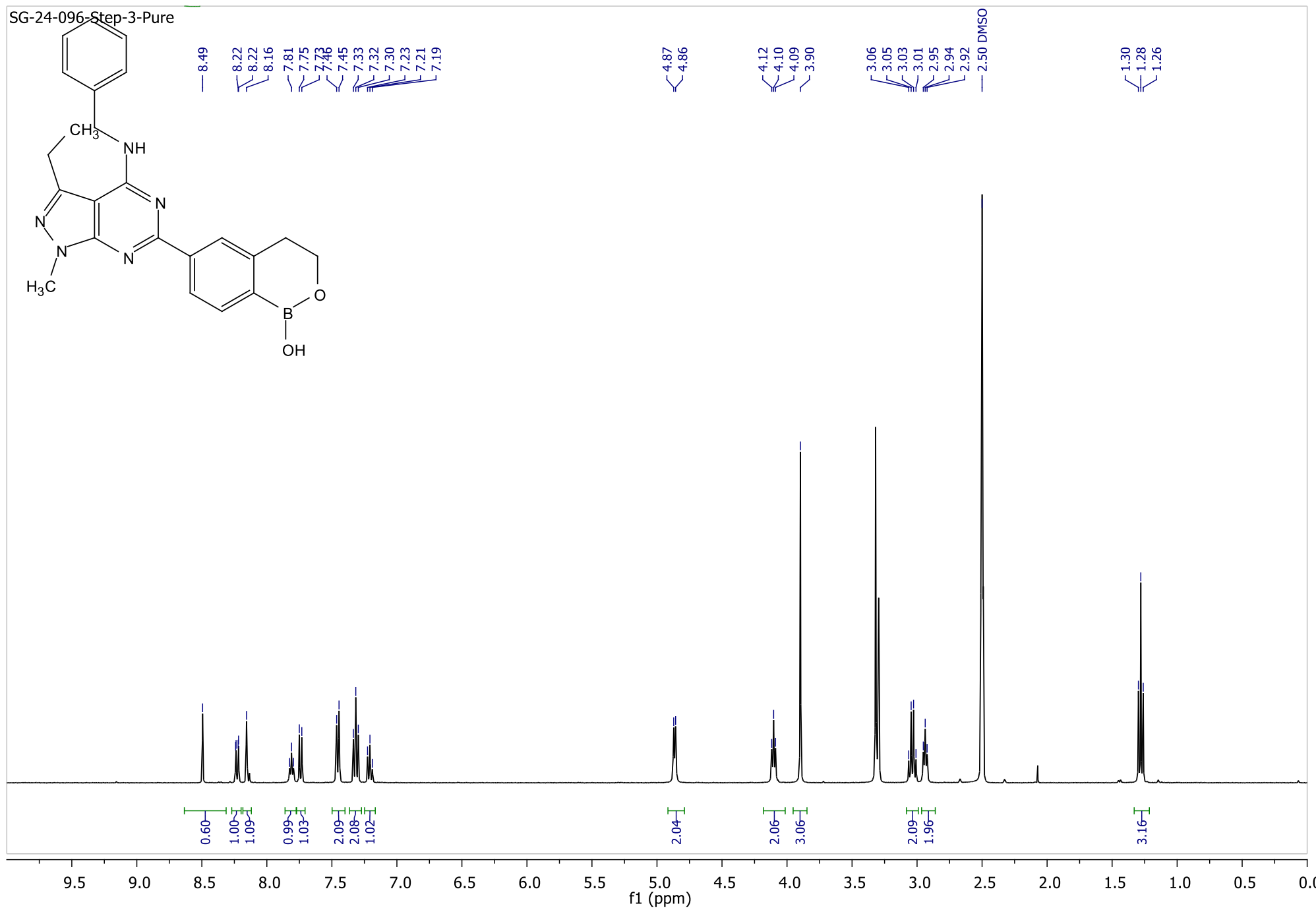

Figure S19: ^{1}H NMR of **35** (400 MHz, DMSO).

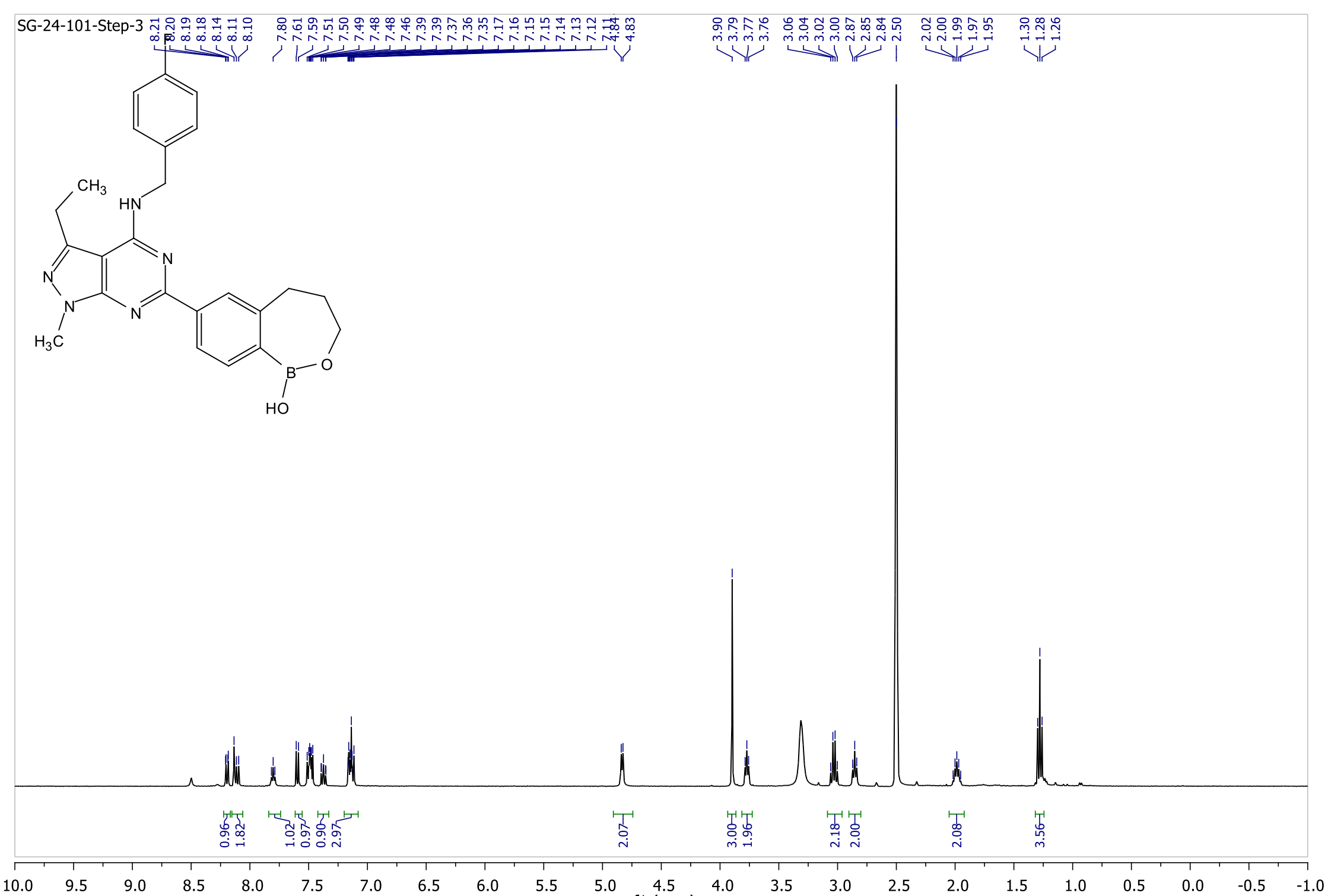

Figure S20: ^{1}H NMR of **38** (400 MHz, DMSO).
